# Synthesis and Anti-*Trypanosoma cruzi* Activity of New Pyrazole-Thiadiazole Scaffolds

**DOI:** 10.3390/molecules29153544

**Published:** 2024-07-27

**Authors:** Thamyris Perez de Souza, Lorraine Martins Rocha Orlando, Leonardo da Silva Lara, Vitoria Barbosa Paes, Lucas Penha Dutra, Mauricio Silva dos Santos, Mirian Claudia de Souza Pereira

**Affiliations:** 1 Laboratório de Ultraestrutura Celular, Instituto Oswaldo Cruz, Fiocruz. Av. Brasil 4365, Rio de Janeiro 21040-900, RJ, Brazil; thamy-perez@hotmail.com (T.P.d.S.); lorrainemartins07@hotmail.com (L.M.R.O.); leonardosilva.lara@hotmail.com (L.d.S.L.); vitpaes@gmail.com (V.B.P.); 2 Laboratório de Síntese de Sistemas Heterocíclicos (LaSSH), Instituto de Física e Química (IFQ), Universidade Federal de Itajubá, Av. BPS 1303, Pinheirinho, Itajubá 37500-903, MG, Brazil; lucaspenhad@gmail.com (L.P.D.); mauriciosantos@unifei.edu.br (M.S.d.S.)

**Keywords:** *Trypanosoma cruzi*, pyrazole, thiadiazole, 3D cardiac microtissue, Chagas disease, parasite recrudescence

## Abstract

Chagas disease, a silent but widespread disease that mainly affects a socioeconomically vulnerable population, lacks innovative safe drug therapy. The available drugs, benznidazole and nifurtimox, are more than fifty years old, have limited efficacy, and carry harmful side effects, highlighting the need for new therapeutics. This study presents two new series of pyrazole-thiadiazole compounds evaluated for trypanocidal activity using cellular models predictive of efficacy. Derivatives **1c** (2,4-diCl) and **2k** (4-NO_2_) were the most active against intracellular amastigotes. Derivative **1c** also showed activity against trypomastigotes, with the detachment of the flagellum from the parasite body being a predominant effect at the ultrastructural level. Analogs have favorable physicochemical parameters and are predicted to be orally available. Drug efficacy was also evaluated in 3D cardiac microtissue, an important target tissue of *Trypanosoma cruzi*, with derivative **2k** showing potent antiparasitic activity and a significant reduction in parasite load. Although **2k** potentially reduced parasite load in the washout assay, it did not prevent parasite recrudescence. Drug combination analysis revealed an additive profile, which may lead to favorable clinical outcomes. Our data demonstrate the antiparasitic activity of pyrazole-thiadiazole derivatives and support the development of these compounds using new optimization strategies.

## 1. Introduction

Chagas disease, a vector-borne zoonotic disease caused by *Trypanosoma cruzi*, is a silent disease recognized as a neglected tropical disease (NTD) by the World Health Organization (WHO). *T. cruzi* is naturally transmitted by more than 150 species of triatomine insects, known as kissing bugs, distributed mainly in the Americas but also found in Asia and Africa [1,2]. Vector control programs suppressed *T. cruzi* domestic transmission by *Triatoma infestans* in Southern Cone countries but oral outbreaks and congenital transmission are currently responsible for the re-emergence of Chagas disease in these endemic countries [3]. This debilitating and neglected disease affects 6–7 million people worldwide and is responsible for high morbidity and mortality rates [4]. Responding to this challenge, the WHO has launched global strategies for 2021–2030; the road map for neglected tropical diseases is 2021–2030, namely to eliminate Chagas disease as a public health problem by establishing effective prevention, control, care, and surveillance in all affected territories [5].

The disease is characterized by an acute phase, which is mostly asymptomatic and typically undiagnosed, and a lifelong chronic phase that can be asymptomatic (indeterminate form) or evolve into a form with cardiac, digestive (megacolon and megaesophagus), neurological, or mixed manifestations. Most people are unaware that they have the disease, as less than 10% of carriers have been diagnosed [6]. Years or decades after infection, about 30–40% of infected individuals develop clinical manifestations, mainly myocardial damage (20–30%), that often results in heart failure [7,8]. Sudden cardiac death (SCD) is the major cause of death in Chagas disease (55–65%) and is mostly associated with ventricular arrhythmia but can also occur in individuals without previous symptoms [9].

Chagas disease chemotherapy is based on the administration of two old nitroheterocyclic drugs, benznidazole (Bz) and nifurtimox (Nif). Despite the well-documented benefits of drug treatment, including 60 to 80% cure in the acute phase, the prevention of congenital transmission in treated mothers, serological cure in infants and children, and reduction in the progression of megasyndromes in people treated in the acute phase, no clinical benefits for patients in the late stage of chronic disease have been reported [10]. The BENEFIT study showed that Bz treatment does not prevent the progression of Chagas cardiomyopathy in symptomatic chronic patients [11]. Drug tolerability is also a major limitation of treatment, with a high incidence of serious adverse effects such as skin lesions, gastrointestinal symptoms, and nervous system disorders, leading to a dropout rate of 10–25% [12]. Thus, a dire need exists for new drugs to fight Chagas disease.

The intensive search for new, effective, and safe drugs has yet to lead to the discovery of any approved innovative drugs. Efforts have been concentrated in the search for drug repurposing, combined therapy, and the discovery of new drugs with potent antiparasitic activity. Findings from clinical trials conducted with potential candidates have been unexpected, resulting in therapeutic failure or the poor tolerability of candidate drugs [13,14,15,16,17,18]. Thus, alternative therapeutic strategies for the management of Chagas disease are essential to benefit this neglected population. Only a few promising candidates are in the clinical development pipeline [19,20]. Hit-to-lead projects have been encouraged to identify small molecules (hits) that can be optimized for lead compounds and progress through the drug discovery pipeline. Heterocyclic compounds, mainly nitro-heterocyclic and azole heterocyclic compounds, have aroused great interest in medicinal chemistry due to their high antiparasitic effect [21]. Pyrazole and thiadiazole scaffolds have attracted attention due to their pharmacological potential. Both chemical moieties have been highlighted as potent antimicrobial [22,23], antiprotozoal [24,25], anti-cancer [26,27], and anti-inflammatory agents [28,29], among others. The high potency and selectivity of *N*-ethylurea pyrazole derivatives were demonstrated against *Trypanosoma brucei* and *Trypanosoma cruzi*. In bioluminescence assays using a murine model infected with *T. cruzi* Brazil-luciferase strain, the most effective *N*-ethylurea pyrazole derivative showed parasite clearance after 6 days of treatment, indicating a potent action in controlling *T. cruzi* infection [30]. In addition, 1*H*-pyrazolo [3,4-*b*]pyridine analogs exhibited high selectivity against intracellular amastigote forms [31]. Thiadiazole-based derivatives have demonstrated high potency against trypanosomatids with versatile mechanisms of action, such as pteridine reductase [32] and C14-α-sterol demethylase (TcCYP51) inhibitors [33], and the oxidative stress inducer [34]. Moreover, pyrazole 2-amino-1,3,4-thiadiazole hybrids showed antileishmanial and antitrypanosomal activity [35]. The pyridinyl-1,3,4-thiadiazole derivatives showed remarkable activity against *T. cruzi*, leading to significant phenotypic changes without generating reactive oxidative species [33].

Our research group has invested in optimizing pyrazole-based scaffolds to identify potentially active compounds against *T. cruzi*. The 5-amino-pyrazole-imidazoline structure was identified as a hit compound, whose most effective 3,4- and 3,5-dichlorides derivatives showed the property of binding the catalytic site of cruzain by molecular docking and inhibiting cysteine protease activity [36]. Reducing the polarity of the hit compound by removing the amino group (-NH_2_) improved the antiparasitic activity with the 4-Br and 3-Cl-4-CH_3_ substituents, showing efficacy against intracellular amastigotes but low activity against trypomastigotes [37]. Also, the replacement of the imidazoline ring by thiazoline, by exploiting the ring bioisosterism strategy, potentiated the activity against trypomastigotes, with 2,4-dichloro pyrazole-thiazoline being more effective against intracellular amastigotes [38]. Herein, we report our design of two new pyrazole-thiadiazole series, **1(a-l)** and **2(a-l)**, and evaluate their anti-*T. cruzi* potential. The structural planning was based on medicinal chemistry tools. Firstly, we decided to keep 1-aryl-1*H*-pyrazole moiety, with or without the amino group (NH_2_), which is found in several compounds previously published by our research group. By exploiting the bioisosterism concept, the imidazoline/thiazoline rings were replaced with a 1,3,4-thiadiazole nucleus, another remarkable pharmacophoric scaffold. The most active derivatives, **1c** and **2k**, showed oral bioavailability prediction and high efficacy in 3D cardiac microtissue, with **2k** delaying parasite recrudescence. 

## 2. Results and Discussion

### 2.1. Chemistry

**Scheme 1:**
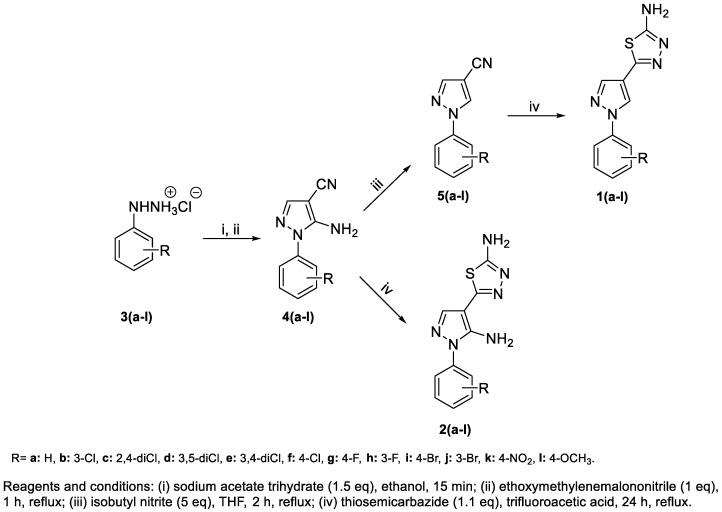
Synthetic route to obtain **1(a-l)** and **2(a-l)**.

The synthesis of the new 5-amino-2-(1-aryl-1*H*-pyrazol-4-yl)-1,3,4-thiadiazoles **1(a-l)** and 5-amino-2-(5-amino-1-aryl-1*H*-pyrazol-4-yl)-1,3,4-thiadiazoles **2(a-l)** are summarized in Scheme 1. The synthetic route started with commercially available arylhydrazine hydrochlorides **3(a-l)**, which first reacted with sodium acetate trihydrate and then with ethoxymethylenemalononitrile through an addition–elimination Michael-type reaction, followed by cyclization, to furnish the key intermediates 5-amino-1-aryl-1*H*-pyrazole-4-carbonitriles **4(a-l) [39]**. In the next step, these compounds were submitted to an aprotic deamination reaction, with tetrahydrofuran (THF) and isobutyl nitrite, to afford 1-aryl-1*H*-pyrazole-4-carbonitriles **5(a-l) [40]**. Finally, **5(a-l)** and **4(a-l)** were converted into the planned compounds **1(a-l)** and **2(a-l)**, respectively, using thiosemicarbazide and trifluoroacetic acid through a cyclization reaction in 20–91% yields [41].

### 2.2. Prediction of Physicochemical Properties

**Figure 1:**
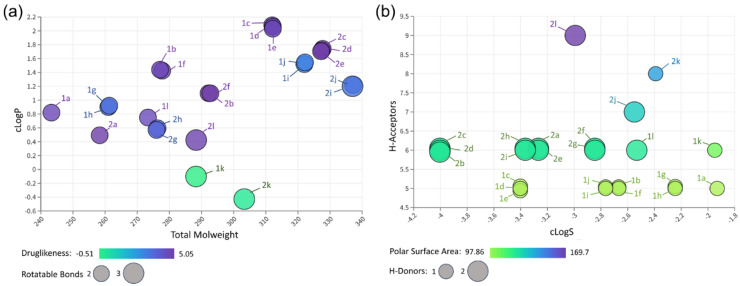
Physicochemical properties of the pyrazole-thiadiazole derivatives. (**a**) Data on lipophilicity (cLogP), molecular weight, rotatable bonds, and druglikeness. (**b**) Water solubility (cLogS), polar surface area, hydrogen bond donors (H-donors), and acceptors (H-acceptors) values for all investigated molecules. The values of each physicochemical parameter were calculated based on the SMILES codes for each compound using the DataWarrior program. The graphs were generated by plotting the values on the online data visualization platform Flourish.

The selection of drug candidates with optimal drug-likeness properties has reduced drug development failures over the past 20 years [42]. Thus, the physicochemical characterization of the new pyrazole-thiadiazole derivatives (series 1 and 2) was performed to identify substances with predicted oral bioavailability. Lipinski’s rule, one of the most acceptable drug-likeness filters, defines four rules (molecular weight (MW) ≤ 500, lipophilicity (clogP) ≤ 5, number of hydrogen bond donors (HBD) ≤ 5, and number of hydrogen bond acceptors (HBA) ≤ 10) to predict drugs’ oral bioavailability. The violation of more than two rules may result in poor oral bioavailability. Our in silico analysis, using DataWarrior software (version 5.5.0), revealed that the small molecules have a low molecular weight (MW), ranging from 243.29 to 337.20 g/mol (Figure 1). Most derivatives have an optimal lipophilicity (cLogP) range between 1.09 and 2.03, which may favor gastrointestinal absorption. Furthermore, HBD and HBA values ranged from 1 to 4 and 5 to 9, respectively. The topological polar surface area (tPSA), which plays an important role in passive molecular transport across membranes, reached values ≤ 140 Å^2^, except for derivatives **1k** (143.68 Å^2^) and **2k** (169.7 Å^2^). Comparing series 1 and 2, the derivatives containing nitro group (-NO_2_) are less lipophilic, reaching cLogP values of −0.10 (**1k**) and -0.42 (**2k**). The addition of the amino group (-NH_2_) to series 2 also reduced the cLogP values, showing more hydrophilic characteristics, mainly derivatives **2a** (0.49), **2l** (0.42), **2g** (0.59), **2h** (0.59), and **2k** (−0.42) (Figure 1). Another evaluated parameter refers to the flexibility of the compounds, determined by rotational bonds (RB). Most derivatives (83%) had RB values equal to 2, except derivatives containing -NO_2_ (**1k** e **2k**) and methoxy (CH_3_O; **1l** e **2l**) groups.

### 2.3. Cytotoxicity and Antiparasitic Effect of Pyrazole-Thiadiazole Derivatives

The Vero cell model was employed to evaluate the cytotoxicity of derivatives **1(a-l)** and **2(a-l)** in mammalian cells. Cell viability, measured by quantifying ATP levels with the CellTiter Glo kit, was assessed after 72 h of treatment of cell monolayers with increasing concentrations of pyrazole-thiadiazole derivatives (15.62–500 µM). The results revealed low toxicity of the pyrazole-thiadiazole derivatives. All derivatives analyzed showed a CC_50_ value greater than 500 µM (Table 1).

The antiparasitic effect of pyrazole-thiadiazole derivatives was evaluated against trypomastigotes and intracellular amastigotes. *T. cruzi*, clone Dm28c-Luc, genetically modified to express luciferase, allowed rapid evaluation of anti-*T. cruzi* activity with high sensitivity and reproducibility. Pyrazole-thiadiazole derivatives, **1(a-l)** and **2(a-l)**, showed low biological activity against trypomastigotes. Derivative **1c** (IC_50_ = 21.71 ± 2.94 µM) stands out with better activity than **1l** (IC_50_ = 53.03 ± 4.44 µM) and **2e** (IC_50_ = 72.62 ± 6 .76 µM) against trypomastigotes (Table 1), with a selectivity index (SI) greater than 23. Analog **1c** has lower efficacy than Bz (IC_50_ = 12.82 ± 2.66 µM) (Table 1).

Activity against intracellular amastigotes was assessed in Vero cells infected with Dm28c-Luc (24 h). The effect of pyrazole-thiadiazole derivatives was determined in *T. cruzi*-infected cell monolayers after 72 h of treatment with a concentration–response curve ranging from 0.86 to 70 µM. Three derivatives showed activity with IC_50_ < 20 µM. Derivatives **1c** (IC_50_ = 13.54 ± 4.47 µM) and **1e** (IC_50_ = 18.75 ± 2.28 µM) of series 1, consisting of the substituents 2,4- and 3,4-dichloro, respectively, and **2k** (IC_50_ = 10.37 ± 1.21 µM) from series 2, with 4-NO_2_ substituent, were the most active derivatives (Table 1). The reference drug (Bz) showed IC_50_ values of 3.61 ± 1.25 µM. Derivatives **1c** and **2k** have SI > 30, with **2k** showing the highest potency (pIC50 = 4.98) (Table 1). 

Regarding physicochemical properties, **1c**, **1e**, and **2k** showed distinct cLogP, tPSA, and HBA parameters. Derivatives **1c** and **1e** have optimal cLogP (2.03), tPSA (97.86 Å^2^), and HBA (5) values, with a prediction of membrane permeability and intestinal absorption. The **2k** derivative has a lower cLogP value (−0.42) but with higher values of tPSA (169.7 Å^2^) and HBA (9), conferring a more hydrophilic profile. 

### 2.4. Structure–Activity Relationship by Similarity 

**Figure 2:**
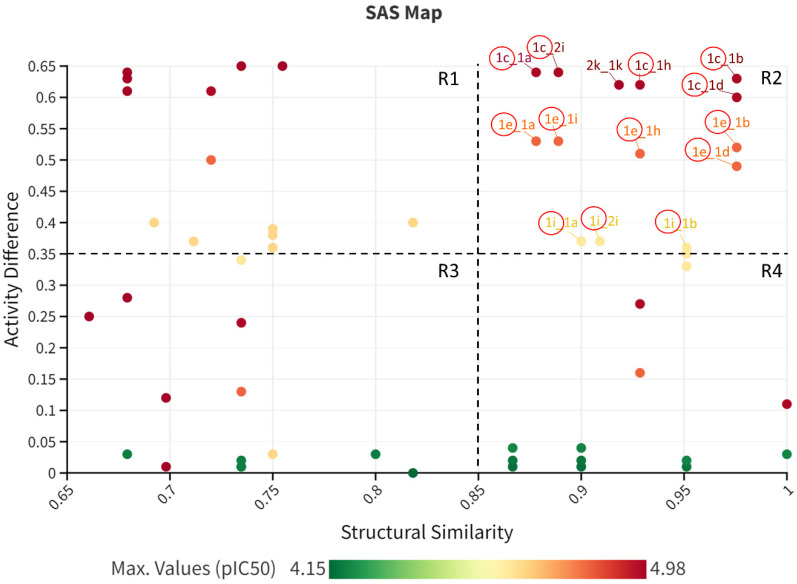
Structure–activity similarity map (SAS map) based on the potency of pyrazole-thiadiazole derivatives against intracellular amastigote forms of *T. cruzi*. Each point represents a comparison of biological activity between structurally similar pairs of pyrazole-thiadiazole derivatives. Colors represent the maximum pIC50 value of the analyzed pair using a low (green) to high (red) power scale. The pairs are distributed in four quadrants classified as R1 = not descriptive; R2 = similar structure and different activity (Cliff activity); R3 = Different structure and similar activity (Similarity cliff); and R4 = Similar structure and activity (Smooth SAR).

The structure–activity and similarity map (SAS map) was a computational tool used to identify the structure–activity relationship (SAR). The SAS map, based on the potency (pIC50) of the compounds against intracellular amastigotes (color gradient), allowed the identification of compounds that exhibit structural similarity and distinct biological activity (Figure 2). In this analysis, the twenty-four pyrazole-thiadiazole derivatives (series 1 and 2) were grouped in pairs on the activity landscape graph, which integrates the relationship between compound structure and potency (pIC50). The graph regions (quadrants) are represented as R1, which corresponds to low structural similarity and different biological activity; R2, which refers to high structural similarity and different biological activity; R3, has low structural similarity and similar biological activity; and R4, characterized by high structural similarity and similar biological activity. Importantly, we designated chemicals that form at least five activity cliff pairs as activity cliff generators, defined as a pair of structurally similar molecules, often associated with cliffs, that show significant changes in potency (R2 region). Based on this criterion, **1c** (pIC50 = 4.87) and **1e** (pIC50 = 4.73) were identified as Cliff generators (Figure 2). It should be noted that **2k** (pIC50 = 4.98) does not appear multiple times in R2, pairing only with **1k** (pIC50 = 4.23), which has more remarkable structural similarity (>85%) with the presence of -NO_2_ as a substituent; but, it is the most potent derivative. The addition of dichlorine, 2,4-diCl (**1c**), 3,4-diCl (**1e**), and 4-NO_2_ (**2k**) substituents on the phenyl ring showed a positive effect on biological activity. However, **1c** and **2k** better fit the recommended cut-off profile for preclinical trials [43,44] so they were selected to proceed with more robust cell models for efficacy analysis. Structural changes in active compounds can interfere with their biological activity, increasing or reducing their potency. Dichlorinated 5-amino pyrazole-imidazoline derivatives (3,4-diCl (pIC50 = 4.86) and 3,5-diCl (pIC50 = 4.78), identified as cysteine protease inhibitors of *T. cruzi*, highlighted the chlorine atom in the *meta* position of the phenyl ring, influencing biological activity [36]. However, the removal of its amine group (4-NH_2_) leads to greater antiparasitic activity in derivatives containing 4-Cl (pIC50 = 5.22), 4-Br (pIC50 = 5.56), and 3-Cl, 4-CH_3_ (pIC50 = 5.45) [37]. The replacement of imidazoline by the thiazoline ring improved the potency of the dichlorinated derivative without an amine group (2,4-diCl; pIC50 = 5.85), with a high selectivity index (SI = 76.2) [38]. Most of the compounds synthesized by our research group with promising anti-*T. cruzi* activity has the chlorine atom as a substituent, an electron-withdrawing group (EWG) with hydrophobic properties (π coefficient = 0.71). The effect of chlorine in medicinal chemistry and its impact on the biological activity of different drugs, including those with antimicrobial and antiparasitic effects, has been widely discussed [45,46].

### 2.5. Ultrastructural Analysis

**Figure 3:**
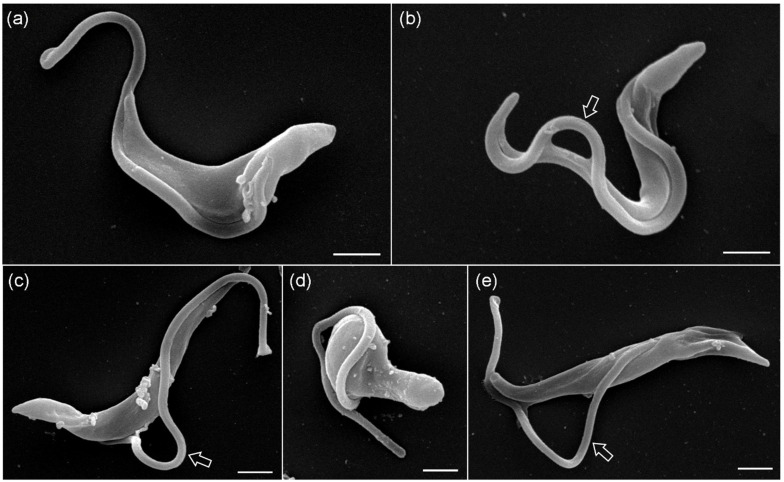
Effect of **1c** on trypomastigotes of *T. cruzi* examined at the ultrastructural level. The parasites were treated for 24 h at concentrations of 21.7 µM (IC_50_) and 54.6 µM (IC_75_) and processed for scanning electron microscopy (SEM). Trypomastigote without drug exposure (**a**). Treatment with **1c** at concentrations of 21.7 µM (**c**) and 54.6 µM (**b**,**d**,**e**) reveals the detachment of the flagellum from the body and changes in parasite morphology. Bars = 1 µm.

Among the selected derivatives, **1c** showed activity against trypomastigote forms of *T. cruzi*. Thus, the parasites were incubated for 24 h with IC_50_ (21.71 µM) and IC_75_ (54.66 µM) concentrations of **1c** and analyzed by scanning electron microscopy (SEM). Ultrastructural analysis demonstrated morphological changes induced by **1c** treatment (Figure 3). The most prominent effect was the detachment of the flagellum from the parasite body, observed in both analyzed concentrations (Figure 3). The effect was evidenced as a partial or total detachment of the flagellum. In addition, some parasites also exhibited changes in morphology, with body rounding, for example (Figure 3). Interestingly, the flagellum detachment was also demonstrated after *T. cruzi* trypanin (*Tc*Trypanin) disruption [47]. Trypanin (54 kDa), first described in *T. brucei* (*Tb*Trypanin) [48], is a component of the nexin–dynein regulatory complex involved in parasite motility [49]. In addition to its role in motility, *Tb*Trypanin also interferes with cytokinesis, suggesting that enzymatic activities, such as ATPases, kinases, and phosphatases, involved in the flagellum beating may be a potential drug target [50]. Deletion of *Tc*Trypanin by the CRISPR/Cas9 system disturbs epimastigote motility and growth. Additionally, the *Tc*Trypanin^−/−^ mutants drastically impair parasite differentiation; the protein knockdown impacts *T. cruzi* infectivity, which was attributed to a change in the parasite motility. Despite trypomastigote release reduction, *Tc*Trypanin deletion does not seem to interfere with the parasite’s life cycle [47,51]. Likewise, *T. cruzi* GP72, a membrane glycoprotein concentrated in the flagellar attachment zone (FAZ region), regulates flagellum adhesion to the parasite body [52,53]. FAZ region has been reported to be responsible for cell organization and cytokinesis [54]. The Knockdown of Fla-1, FAZ1, FAZ5, or FS179/TbCaCh, proteins that constitute the FAZ region in *T. brucei* by RNA interference (RNAi) leads to the detachment of the flagellum from the body and failure in cytokinesis [54,55]. Although structurally well characterized, the constituents of the FAZ region are poorly elucidated. As in *Tc*Trypanin knockouts, GP72 null mutants also provoke the flagellum detachment. Furthermore, H49 proteins, classified as calpain-like (CALP), are another FAZ component in the flagellum attachment to the cell body [56]. Thus, a structural role has also been suggested for H49/calpain, promoting the connection of subpellicular microtubules with FAZ cytoplasmic filaments. Curiously, suramin, an anti-parasitic drug that inhibits several enzymes, endocytic process, and LDL binding and interferes with cell division in trypanosomes [57,58], has been reported to alter trypomastigotes shape, leading to the partial or total detachment of the flagella [59]. Our findings demonstrate changes in flagellum attachment and loss of parasite viability, suggesting that the pyrazole-thiadiazole derivatives may also act on FAZ region-related structural proteins or regulatory enzymes.

### 2.6. The Toxicity of Pyrazole-Thiadiazole Selected Candidates on Cardiac Muscle Cells

The cardiotoxicity-inducing effect is an important cause of drug withdrawal required by pharmaceutical companies due to safety concerns [60]. Adverse effects can cause cardiac muscle dysfunction and lead to heart failure. Thus, 2D and 3D models of a primary culture of cardiac muscle cells were used to assess the toxicity profile of selected pyrazole-thiadiazole candidates. Cell monolayers (2D) and cardiac spheroids (3D) were incubated for 72 h with **1c** and **2k** at concentrations ranging from 15.62 to 500 µM, with cell viability measured by ATP levels. The results revealed the low cardiotoxicity of these derivatives. The **1c** exhibited CC_50_ values greater than 500 µM in both cell models (2D and 3D) (Table 2). However, **2k** showed different CC_50_ values, reaching CC_50_ = 191.20 ± 21.0 and CC_50_ > 500 µM in the cell monolayers and 3D cardiac microtissue, respectively (Table 2). Different platforms and cellular models have been exploited to evaluate the risk of cardiotoxicity of newly developed drugs. Standard cell cultures, mainly from cardiomyocyte cell line (H9C2 cell) monolayers, are a widely used strategy for analyzing the drugs’ cardiotoxic effect [61,62]. However, more robust cellular systems, such as 3D microtissues, organ-on-a-chip, and bioprinting technologies, potentially generate more reliable data and improve the prediction of efficacy and adverse effects [63,64]. The 3D technology using rodent or human primary or even human induced pluripotent stem cell-derived cardiomyocytes (hiPSC-CMs), which better represent cardiac physiology and microenvironment, has shown increasing application in the drug cardiac safety assessment, contributing to fewer drug adverse effects and reducing drug failures in late-stages of development [65,66].

### 2.7. Pyrazole-Thiadiazole Efficacy in T. cruzi-Infected 3D Cardiac Microtissue

Preclinical trials for new drug development have highlighted differential drug responses between 2D and 3D culture models. Oncology drug screening, for example, has demonstrated low drug susceptibility of three-dimensionally organized tumor cells, which may be related to the difficulty some molecules have in permeating the 3D culture of multicellular spheroids [67,68]. 

**Figure 4:**
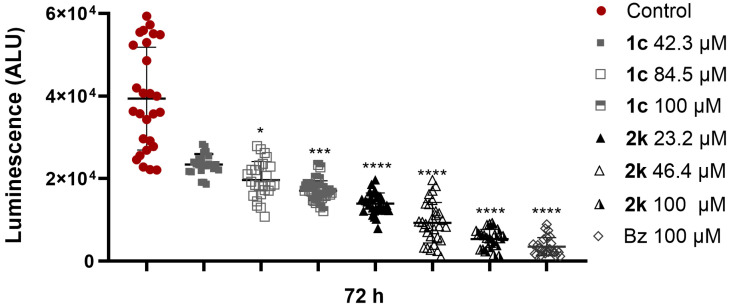
Anti-*T. cruzi* activity of **1c** and **2k** in 3D cardiac microtissue. The viability of intracellular parasites was assessed by measuring the luminescence signal arising from luciferase activity (ALU–Arbitrary Luminescence Unit). (*) The statistical significance compared to the untreated group was assessed using the one-way ANOVA test followed by the Kruskal–Wallis test, with significance levels expressed as (*) *p* < 0.05, (***) *p* < 0.001, and (****) *p* < 0.0001.

**Figure 5:**
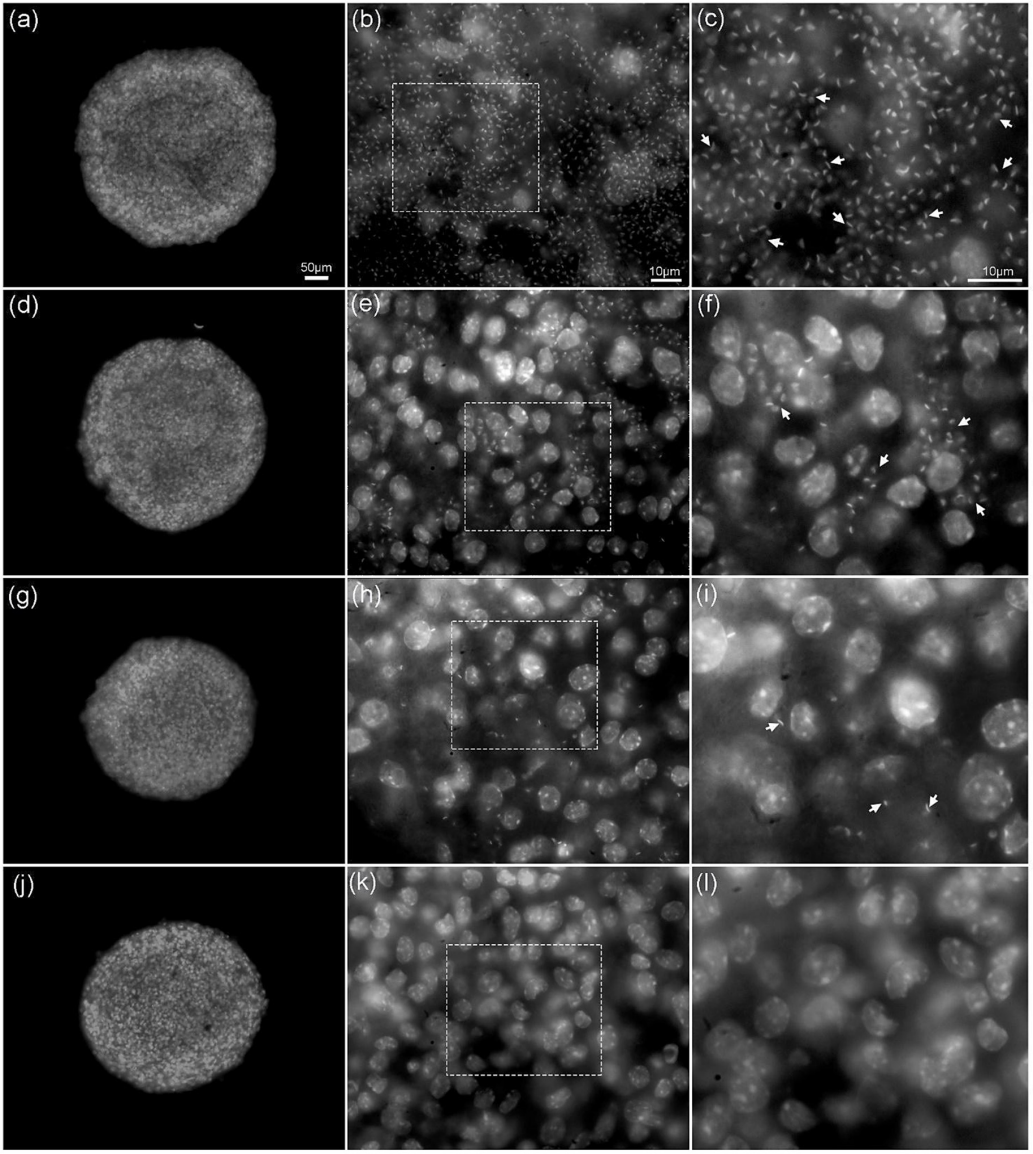
Effect of **1c** and **2k** in the 3D cardiac microtissue infection model. Fluorescence microscopy images stained with DAPI of untreated *T. cruzi* (Dm28c-Luc)-infected cardiac spheroids (**a**–**c**) and treated for 72 h with **1c** (**d**–**f**), **2k** (**g**–**i**) and Bz (**j**–**l**). Untreated 3D cardiac spheroids (**a**–**c**) display an infection profile with large numbers of intracellular amastigotes (white arrow). Treatment with compounds **1c** (**d**–**f**) and **2k** (**g**–**i**) reduced the infection profile, with **2k** drastically reducing the intracellular parasites (white arrows). Few intracellular amastigotes were observed in microtissues treated with Bz (**j**–**l**). Bar = 10 µm and 50 µm.

Since drug tissue permeability is crucial in developing new compounds, the imperfect penetration of antimicrobial drugs into tissues and organs limits their distribution and effectiveness. Therefore, the ability of **1c** and **2k** to eliminate intracellular parasites was also assessed in *T. cruzi*-infected scaffold-free 3D cardiac spheroids. The infected spheroids (24 h; Dm28c-Luc) were incubated for 72 h with concentrations of the selected derivatives, **1c** and **2k**, corresponding to IC_90_ and two times the IC_90_ and 100 µM. The effect of the derivatives against intracellular parasites was determined after adding luciferin, a luciferase enzyme substrate, and the luminescent signal reading expressed in arbitrary luminescence units (ALU). The results demonstrated that **1c** and **2k** can reduce parasite load in 3D cardiac microtissue (Figure 4). A high luminescent signal, representing a high infection profile, was revealed in drug-free infected spheroids (96 h) (ALU_mean_ = 39,402 ± 12,437). Parasite load was significantly reduced in 3D cardiac spheroids treated with different concentrations of **1c**, with a maximum inhibition (57%) achieved with 100 µM (ALU_mean_ = 17,078 ± 2407) when compared to *T. cruzi*-infected and -untreated 3D cardiac microtissue (Figure 5). Derivative **2k** was more effective in clearing intracellular amastigotes than **1c**, achieving an 86% reduction in the viability of intracellular parasites at a 100 µM concentration (ALU_mean_ = 5366 ± 2348). At the highest concentration (100 µM), **2k** showed an effect like Bz (ALU_mean_ = 3520 ± 2198), which revealed 91% inhibition of 3D cardiac microtissue infection (Figure 4).

Only some studies exploit the 3D system as a tool for analyzing the effectiveness of antiparasitic drugs. Three-dimensional murine peritoneal macrophages cultivated in Alvetex scaffolds were employed to evaluate the activity of leishmanicidal agents (amphotericin B, miltefosine, paromomycin sulfate, and sodium stibogluconate) [69]. Among the drugs analyzed, there was a 33% increase in the EC_50_ value of 3D macrophage cultures (EC_50_ = 52.3 nM) treated with amphotericin B when compared to the 2D model (EC_50_ = 34.9 nM), demonstrating that the cellular complexity impacts on the anti-Leishmania drug’s efficacy. In another study employing the human hepatocarcinoma cell line (HepG2), the 3D model of hepatic cells was used to infer the treatment dose of M5717, a *Plasmodium* elongation factor 2 inhibitor, in mice challenged with *Plasmodium berghei* [70]. The in vitro IC_99_ value was applied in a population pharmacokinetics modeling and simulation approach, based on Phase 1 clinical data, to calculate the therapeutic dose in vivo. In 2021, the estimated dose of M5717 entered the VIS liver stage induced by *Plasmodium* sp (NCT04250363), highlighting the relevance of the 3D model in the translational approaches to predict drug efficacy. Three-dimensional spheroids have also been exploited to evaluate drug efficacy against *T. cruzi*. Pretreatment of HeLa spheroids with resveratrol (40 µM), a bioactive compound found in some foods (grape, strawberry, and raspberry) and in red wine, reduces the *T. cruzi* infection level (50%) but did not impair trypomastigote transmigration to spheroid inner layers [71]. The efficacy of pyrazole-imidazoline and pyrazole-thiadiazole derivatives has been demonstrated in *T. cruzi*-infected spheroids generated from Vero cells [37,38]. The most effective pyrazole-imidazoline derivative (3-Cl,4-CH_3_), with oral bioavailability prediction, led to a 93% reduction in parasite load in 3D Vero cultures [37]. Similar results were achieved with pyrazole-thiazoline derivative (2,4-diCl) that showed 98% inhibition of the infection level in 3D microtissue [38]. Additionally, the effect of Bz, the reference drug, was evaluated regarding its efficacy and the modulation of inflammatory mediators in 3D cardiac spheroids infected by *T. cruzi* [72]. Treatment with Bz (10 µM) reduced parasite load by 92% and led to downregulation of interleukin 6 (IL-6) and tumor necrosis factor-alpha (TNF-α). Thus, incorporating the 3D culture model into the screening platform may allow for greater drug efficacy and toxicity predictability. 

The drug’s inhibitory effect was also evaluated by fluorescence microscopy. The staining of infected 3D cardiac spheroids with DAPI, a DNA dye, allowed the visualization of the nuclei of host cells and the nucleus and kinetoplast of intracellular parasites (Figure 5). The presence of many intracellular amastigotes revealed a high infection level in the outermost spheroid layers (Figure 5). However, the fluorescence microscope images obtained in the Z-axis demonstrated that, in addition to the infection in the peripheral region, the intracellular parasites were also identified in the innermost layers of the 3D cardiac microtissue (Supplementary data, Figure S1). Infection profile reduction was observed in the cardiac spheroids treated with **1c** (100 µM) and **2k** (100 µM). Conventional fluorescence imaging showed a decrease in amastigote nests from **1k**-treated spheroids compared to the untreated *T. cruzi*-infected spheroids (Figure 5). The parasitic load-reducing effect is even more expressive in 3D cardiac spheroids treated with **2k**, showing few intracellular parasites. Rare parasites are observed in 3D cardiac microtissues treated with Bz (100 µM). The effect of the **2k** treatment was most evident in the Z-series of optical sections (Supplementary data, Figure S2). It is possible to observe a decrease in the infection level of spheroids treated with **2k** (84 µM). The Z-axis images revealed rare parasites in the innermost layers of the *T. cruzi*-infected 3D microtissue treated with **2k** and Bz (Supplementary data, Figures S2 and S3). 

### 2.8. Ability of Pyrazole-Thiadiazole to Prevent Reactivation of Infection

**Figure 6:**
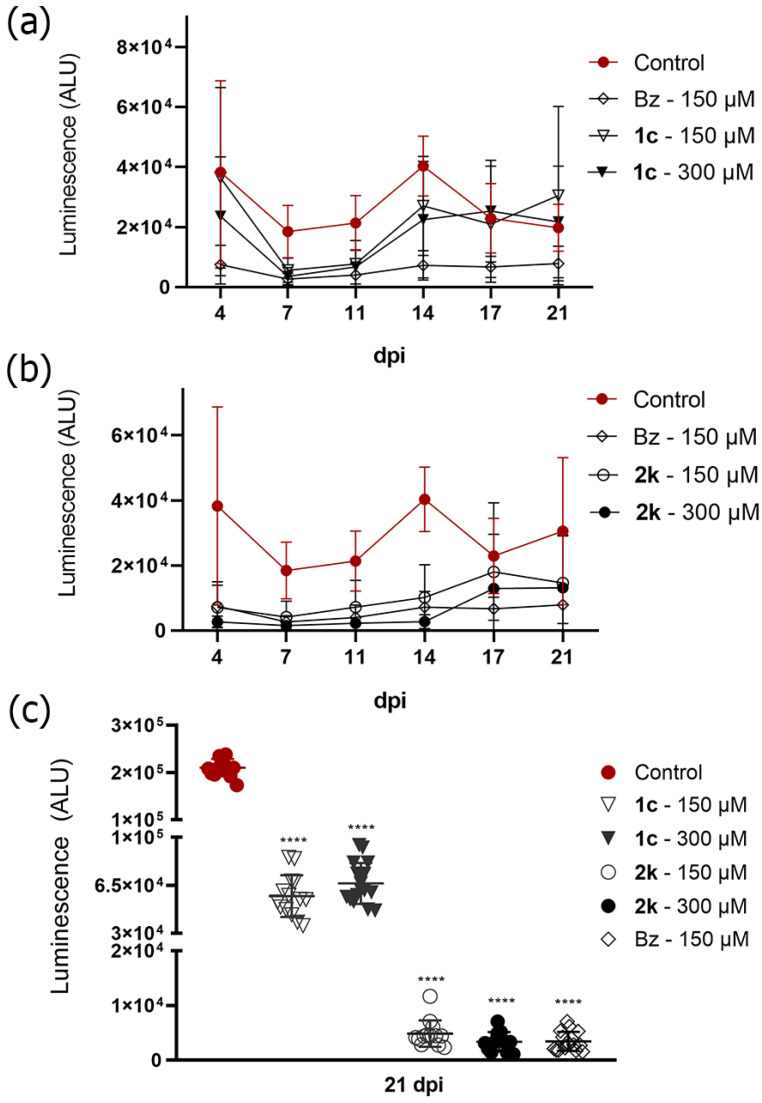
Recovery assay (washout) with pyrazole-thiadiazole derivatives. *T. cruzi*-infected Vero cell cultures were subjected to 10 days of treatment with **1c** and **2k**, followed by 10 days of cultivation without compounds. The presence of trypomastigotes in the supernatant was evaluated in untreated and *T. cruzi*-infected cultures treated with **1c** (**a**) and **2k** (**b**) every 3–4 days post-treatment. The effect of compounds was also demonstrated in the cell monolayer (**c**). Luminescence values corresponding to the viability of the parasites are expressed as arbitrary luminescence units (ALU). (*) The statistical significance compared to the untreated group was assessed using the one-way ANOVA test followed by the Kruskal–Wallis test, with significance levels denoted as (****) *p* < 0.0001.

Derivatives **1c** and **1k** were also evaluated for their ability to inhibit the resurgence of the parasite. Thus, cultures of Vero cells infected by *T. cruzi* (Dm28c-Luc) were treated for 10 days with the selected compounds, **1c** and **2k**, at 150 µM and 300 µM, corresponding to 3.5 to 13 times the IC_90_. The maximum concentration represents values lower than the CC_20_ for Vero cell monolayers (>500 µM). During the treatment period (10 days), the compounds were replaced every 3–4 days, followed by an additional period of 10 days of cultivation with no drug pressure. The results of the washout assay demonstrated that both **1c** and **2k** were able to significantly reduce the parasite load in the cell monolayer compared to the *T. cruzi*-infected and untreated group (ALU_mean_ = 210,424 ± 18,379) (Figure 6). Vero cell monolayers treated with **2k** at concentrations of 150 µM (ALU_meann_ = 4845 ± 2419) and 300 µM (ALU_mean_ = 3349 ± 1767) showed activity 11.8 and 19.8 times greater than **1c** (150 µM–ALU average = 57,103 ± 15,228; 300 µM–= 66,378 ± 14,991), respectively (Figure 6). Treatment with **2k** achieved an effect like that observed in Bz treatment (150 µM; ALU_mean_ = 3426 ± 1727).

The release of parasites into the culture supernatant was also monitored during treatment with selected derivatives (10 days) and, subsequently, for another 10 days without drug pressure. Treatment with **1c**, at concentrations of 150 and 300 µM, resulted in an inhibition of 52% and 66% in the release of trypomastigotes, respectively, 11 days after infection (dpi), compatible with the inhibitory effect of Bz (77%). However, upon the compound’s removal, trypomastigote release quickly returned to levels seen in the untreated infected group. The **2k** derivative maintained parasite release levels like Bz up to 14 dpi, mainly at a concentration of 300 µM, achieving a 93% reduction compared to the untreated infected group, with Bz reaching 82% inhibition (Figure 6). After 10 days of drug withdrawal (21 dpi), the parasite release level of **2k** (150 and 300 µM) remained like the reference drug (Bz) (Figure 6).

The translational relevance of a washout assay has been highlighted in identifying candidates with promising trypanocidal activity [73]. Prolonged treatment with posaconazole, for example, was not able to prevent the reactivation of the infection in vitro [74], corroborating the data on trypanostatic activity evidenced in the clinical trial with resurgence of parasitism in the absence of drug pressure [14,15]. Studies with purine nucleoside analogs, which revealed a reduction in parasitemia, and the survival of animals treated with 2.5 and 25 mg/kg of the Cpd1 analog demonstrated a 17% sterile cure (negative PCR reaction) and inability to prevent reactivation of the infection in the in vitro washout assay, probably related to the presence of dormant parasites [75]. Pyrazole-thiadiazole candidates were also unable to prevent the reactivation of infection after drug withdrawal, with **1c** exhibiting a trypanostatic action. Derivative **2c** showed similar results to Bz, reducing the release of trypomastigotes (≥82%) but without achieving a sterile cure, suggesting low efficacy in dormant parasites.

### 2.9. Drug Combination Effect

**Figure 7:**
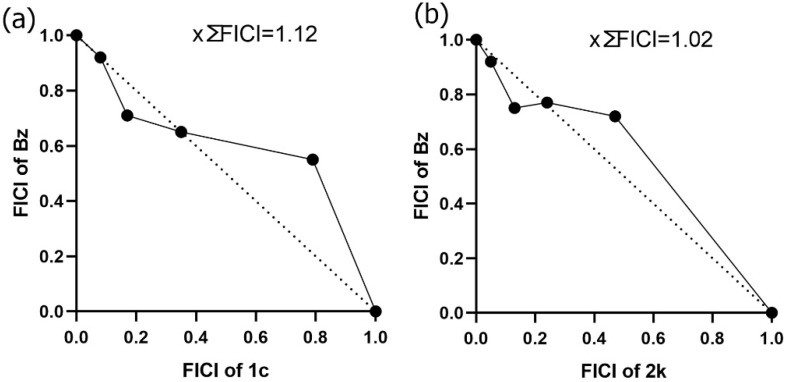
In vitro combinatorial activity of **1c** (**a**) and **2k** (**b**) with Bz against amastigote forms of *T. cruzi*. FICI values were calculated based on the IC_50_ values of the combinations. The isobologram graph was plotted with FICI values. The dashed line represents a range of additive values between the FICIs of the compounds.

Despite the effectiveness of long-term therapies in the acute phase of Chagas disease, Bz toxicity is one of the main reasons for patients’ low adherence to treatment. One strategy to overcome this gap is the search for compounds that, combined with Bz, can reduce its dosage and treatment period, improving cure rates and reducing adverse effects [76,77]. Therefore, we evaluated the efficacy of the combined in vitro treatment between promising pyrazole-thiadiazole candidates and Bz. Combinations between concentrations of selected compounds and Bz (5:0, 4:1, 3:2, 2:3, 1:4, and 0:5) were prepared and *T. cruzi*-infected Vero cell cultures were subjected to combined treatment for 72 h. The values of the mean sums of fractional inhibitory concentrations (x∑FICI) demonstrated that both combinations are classified as additive with x∑FICI = 1.12 and 1.02 for **1c**-Bz and **2k**-Bz, respectively. The isobologram plots corresponding to the FICI values of the compounds in combination are shown in Figure 7. None of the proportions between the selected compounds and Bz reach synergistic activity values.

Studies have demonstrated that the combined therapy strategy in in vivo *T. cruzi* infection models can result in positive outcomes even using combinations of compounds considered additive in the in vitro model [78,79]. In this context, the combination of the sulfone metabolite of fexinidazole (fex-SFN) and Bz, classified as an additive in the in vitro model for *T. cruzi* (Y strain), presents additional advantages compared to monotherapy in the in vivo model [80]. These combinations resulted in faster suppression of parasitemia and higher levels of parasitological cure than monotherapies. Improved efficacy was also reported in the combination of a pyrazolone derivative (NDP-227), a phosphodiesterase inhibitor, and Bz in the acute mouse model of *T. cruzi* infection (Y strain) [81]. The combined treatment of NDP-227 with Bz, identified with an additive profile in the in vitro isobologram, led to approximately 90% parasitemia reduction and an increased survival rate (85%) compared to Bz and NPD-227 monotherapies. Therefore, additional combination therapy studies in the in vivo model between pyrazole-thiadiazole derivatives may represent a promising strategy to improve efficacy.

## 3. Materials and Methods

### 3.1. Chemistry

The melting points were measured in duplicate in Allerbest equipment. The FT-IR spectra were recorded on the PerkinElmer spectrometer (Spectrum 100), with ATR system and ZnSe apparatus. The analyses were performed with 16 scans and a resolution of 4 cm^−1^. The ESI HRMS analyses were accomplished using Q-TOF Micromass/Waters equipment (ZQ-4000). NMR spectra were recorded in a 300 or 400 MHz Bruker Avance spectrometer, using DMSO-*d_6_* as the solvent and TMS as the internal standard. All NMR spectra are shown in supplementary data (Figure S4). The reaction evolution was verified by thin-layer chromatography (TLC) with precoated 60 F254 silica gel plates. All reagents and solvents were commercially acquired and the key intermediates were utilized in the reactions to obtain the planned compounds without further purification. The key intermediates **4(a-l)** and **5(a-l)** were prepared using methodologies previously reported by our research group [39,40].

#### General Procedure for the Synthesis of **1(a-l)** and **2(a-l)**

5-Amino-1-aryl-1*H*-pyrazol-4-carbonitrile **3(a-l)** or 1-aryl-1*H*-pyrazol-4-carbonitrile **4(a-l)** was dissolved in trifluoroacetic acid (2 mL/mmol) with 1.1 eq. of thiosemicarbazide, in a round-bottom flask, which was further connected in an Allihn condenser. The reaction mixture was heated and kept under reflux for 24 h. After that, TLC analysis showed that raw materials were completely consumed. Subsequently, 3 mL of ethanol was added to the flask, and the mixture was poured by stirring into cold water. The precipitate was filtered under reduced pressure, washed with cold water, and dried in a desiccator.

*5-Amino-2-(1-phenyl-1H-pyrazol-4-yl)-1,3,4-thiadiazole* **1a**: Yield: 77%. m.p.: 289–291 °C. FT-IR ν (cm^−1^): 3349, 3089, 2962, 2745, 2647, 1635, 1588, 1505, 1467. ^1^H NMR (400 MHz, DMSO-*d6*) *δ* 9.00 (d, *J* = 0.5 Hz, 1H), 8.17 (s, 1H), 7.91-7.89 (m, *J* = 7.5, 2H), 7.57–7.53 (m, *J* = 7.5 Hz, 2H), 7.40–7.36 (m, *J* = 7.5 Hz, 1H). ^13^C NMR (100 MHz, DMSO-*d6*) *δ* 167.8, 148.3, 138.9, 138.7, 129.5, 126.9, 126.5, 118.5, 115.3. HRMS (ESI) *m/z*: [M+H]^+^ = 244.0656 (found), 244.0651 (calculated).

*5-Amino-2-(1-(3-chlorophenyl)-1H-pyrazol-4-yl)-1,3,4-thiadiazole* **1b**: Yield: 83%. m.p.: 310–311 °C. FT-IR ν (cm^−1^): 3381, 3113, 3060, 2971, 2753, 2712, 2657, 1649, 1584, 1529, 1487. ^1^H NMR (400 MHz, DMSO-*d6*) *δ* 9.07 (s, 1H), 8.17 (s, 1H), 8.02 (t, *J* = 1.5 Hz, 1H), 7.90 (dd, *J* = 8.2, 1.5 Hz, 1H), 7.55 (t, *J* = 8.2 Hz, 1H), 7.41-7,35 (m, 3H). ^13^C NMR (100 MHz, DMSO-*d6*) *δ* 167.8, 148.0, 140.2, 139.2, 134.1, 131.4, 126.8, 126.5, 118.2, 117.0, 116.2. HRMS (ESI) *m/z*: [M+H]^+^ = 278.0263 (found), 278.0262 (calculated).

*5-Amino-2-(1-(2,4-dichlorophenyl)-1H-pyrazol-4-yl)-1,3,4-thiadiazole* **1c**: Yield: 31%. m.p.: 284–285 °C. FT-IR ν (cm^−1^): 3255, 3092, 2940, 2766, 2670, 1617, 1585, 1514, 1498, 1464. ^1^H NMR (400 MHz, DMSO-*d6*) *δ* 8.61 (d, *J* = 1.6 Hz, 1H), 8.16 (d, *J* = 1.6 Hz, 1H), 7.91 (d, *J* = 2.0 Hz, 1H), 7.70-7.67 (m, *J* = 8.5 Hz, 1H), 7.64-7.62 (m, *J* = 8.5 Hz, 1H), 7.30 (s, 2H). ^13^C NMR (100 MHz, DMSO-*d6*) *δ* 167.6, 148.1, 138.8, 136.1, 133.9, 130.6, 129.9, 129.3, 129.2, 128.3, 114.9. HRMS (ESI) *m/z*: [M+H]^+^ = 311.9865 (found), 311.9872 (calculated).

*5-Amino-2-(1-(3,5-dichlorophenyl)-1H-pyrazol-4-yl)-1,3,4-thiadiazole* **1d**: Yield: 79%. m.p.: 283–285 °C. FT-IR ν (cm^−1^): 3291, 3097, 2959, 2666, 1651, 1587, 1505, 1468. ^1^H NMR (400 MHz, DMSO-*d6*) *δ* 9.08 (s, 1H), 8.18 (s, 1H), 7.97 (d, *J* = 1.3 Hz, 2H), 7.54 (pt, *J* = 1.3 Hz, 1H), 7.31 (s, 2H). ^13^C NMR (100 MHz, DMSO-*d6*) *δ* 167.7, 147.6, 140.7, 139.5, 135.0, 127.0, 125.8, 116.8, 116.5. HRMS (ESI) *m/z*: [M+H]^+^ = 311.9853 (found), 311.9872 (calculated).

*5-Amino-2-(1-(3,4-dichlorophenyl)-1H-pyrazol-4-yl)-1,3,4-thiadiazole* **1e**: Yield: 91%. m.p.: 313–315 °C. FT-IR ν (cm^−1^): 3278, 3129, 3095, 2961, 2707, 2658, 1631, 1589, 1519, 1481. ^1^H NMR (300 MHz, DMSO-*d6*) *δ* 9.12 (s, 1H), 8.21-8.19 (m. 2H), 7.91 (dd, *J* = 8.8, 2.6 Hz, 1H), 7.78 (d, *J* = 8.8 Hz, 1H). ^13^C NMR (75 MHz, DMSO-*d6*) *δ* 168.0, 147.9, 139.4, 138.7, 132.1, 131.5, 128.9, 127.2, 120.0, 118.5, 116.1. HRMS (ESI) *m/z*: [M+H]^+^ = 311.9880 (found), 311.9872 (calculated).

*5-Amino-2-(1-(4-chlorophenyl)-1H-pyrazol-4-yl)-1,3,4-thiadiazole* **1f**: Yield: 74%. m.p.: 328–330 °C. FT-IR ν (cm^−1^): 3272, 3089, 2950, 2772, 2680, 1627, 1580, 1519, 1499. ^1^H NMR (300 MHz, DMSO-*d6*) *δ* 9.00 (s, 1H), 8.13 (s, 1H), 7.92 (d, *J* = 8.7 Hz, 2H), 7.57 (d, *J* = 8.7 Hz, 2H), 7.29 (s, 2H). ^13^C NMR (75 MHz, DMSO-*d6*) *δ* 167.7, 148.0, 139.0, 137.9, 130.9, 129.5, 126.53, 120.1, 116.1. HRMS (ESI) *m/z*: [M+H]^+^ = 278.0272 (found), 278.0262 (calculated).

*5-Amino-2-(1-(4-fluorophenyl)-1H-pyrazol-4-yl)-1,3,4-thiadiazole* **1g**: Yield: 58%. m.p.: 335–337 °C. FT-IR ν (cm^−1^): 3267, 3114, 3093, 2960, 2781, 2692, 1635, 1588, 1509. ^1^H NMR (300 MHz, DMSO-*d6*) *δ* 8.94 (s, 1H), 8.11 (s, 1H), 7.91 (dd, *J* = 8.8, 4.7 Hz, 2H), 7.38–7.28 (m, 4H). ^13^C NMR (75 MHz, DMSO-*d6*) *δ* 167.7, 160.5 (d, *J* = 243.6 Hz), 148.2, 138.8, 135.7, 126.5, 120.6 (d, *J* = 8.5 Hz), 116.3 (d, *J* = 23.0 Hz), 115.9. HRMS (ESI) *m/z*: [M+H]^+^ = 262.0567 (found), 262.0557 (calculated).

*5-Amino-2-(1-(3-fluorophenyl)-1H-pyrazol-4-yl)-1,3,4-thiadiazole* **1h**: Yield: 47%. m.p.: 302–303 °C. FT-IR ν (cm^−1^): 3385, 3242, 3101, 2935, 2733, 2652, 1638, 1599, 1522, 1499, 1482, 1461. ^1^H NMR (300 MHz, DMSO-*d6*) *δ* 9.05 (s, 1H), 8.17 (s, 1H), 7.95-7.71 (m, 2H), 7.58 (td, *J* = 8.4, 6.4 Hz, 1H), 7.32 (s, 2H), 7.20 (tdd, *J* = 8.4, 2.4, 1.0 Hz, 1H). ^13^C NMR (75 MHz, DMSO-*d6*) *δ* 167.7, 162.4 (d, *J* = 243.7 Hz), 147.9, 140.4 (d, *J* = 10.4 Hz), 139.0, 131.4 (d, *J* = 9.4 Hz), 126.6, 116.1, 114.2 (d, *J* = 2.9 Hz), 113.3 (d, *J* = 21.2 Hz), 105.7 (d, *J* = 26.8 Hz). HRMS (ESI) *m/z*: [M+H]^+^ = 262.0570 (found), 262.0557 (calculated).

*5-Amino-2-(1-(4-bromophenyl)-1H-pyrazol-4-yl)-1,3,4-thiadiazole* **1i**: Yield: 76%. m.p.: 340–341 °C. FT-IR ν (cm^−1^): 3273, 3089, 2949, 2769, 2677, 1626, 1592, 1517, 1495, 1467. ^1^H NMR (300 MHz, DMSO-*d6*) *δ* 9.02 (s, 1H), 8.15 (s, 1H), 7.86 (d, *J* = 8.9 Hz, 2H), 7.70 (d, *J* = 8.9 Hz, 2H). ^13^C NMR (75 MHz, DMSO-*d6*) *δ* 167.6, 147.9, 138.9, 138.2, 132.3, 126.3, 120.3, 119.0, 116.0. HRMS (ESI) *m/z*: [M+H]^+^ = 323.9748 (M+2 found), 323.9736 (M+2 calculated).

*5-Amino-2-(1-(3-bromophenyl)-1H-pyrazol-4-yl)-1,3,4-thiadiazole* **1j**: Yield: 82%. m.p.: 301–303 °C. FT-IR ν (cm^−1^): 3385, 3262, 3096, 2939, 2763, 2661, 1622, 1589, 1517, 1490, 1451. ^1^H NMR (300 MHz, DMSO-*d6*) *δ* 9.06 (s, 1H), 8.15-8.13 (m, 2H), 7.92 (d, *J* = 7.9 Hz, 1H), 7.54–7.34 (m, 4H). ^13^C NMR (75 MHz, DMSO-*d6*) *δ* 167.8, 148.0, 140.3, 139.1, 131.5, 129.4, 126.8, 122.39, 120.9, 117.4, 116.2. HRMS (ESI) *m/z*: [M+H]^+^ = 323.9742 (M+2 found), 323.9736 (M+2 calculated).

*5-Amino-2-(1-(4-nitrophenyl)-1H-pyrazol-4-yl)-1,3,4-thiadiazole* **1k**: Yield: 20%. m.p.: 331–333 °C. FT-IR ν (cm^−1^): 3267, 3115, 3095, 2957, 2781, 2690, 1632, 1593, 1518, 1505. ^1^H NMR (400 MHz, DMSO-*d6*) *δ* 9.14 (s, 1H), 8.37 (d, *J* = 9.2 Hz, 2H), 8.26 (s, 1H), 8.15 (d, *J* = 9.2 Hz, 2H), 7.35 (s, 2H). ^13^C NMR (100 MHz, DMSO-*d6*) *δ* 168.3, 148.1, 145.4, 143.7, 140.6, 127.4, 125.7, 119.0, 117.2. HRMS (ESI) *m/z*: [M+H]^+^ = 289.0500 (found), 289.0502 (calculated).

*5-Amino-2-(1-(4-methoxyphenyl)-1H-pyrazol-4-yl)-1,3,4-thiadiazole* **1l**: Yield: 56%. m.p.: 298–300 °C. FT-IR ν (cm^−1^): 3402, 3342, 3276, 3113, 2957, 2933, 2835, 2743, 2651, 1611, 1519, 1504, 1459. ^1^H NMR (400 MHz, DMSO-*d6*) *δ* 8.86 (d, *J* = 0.5 Hz, 1H), 8.09 (d, *J* = 0.5 Hz, 1H), 7.80 (d, *J* = 9.1 Hz, 2H), 7.08 (d, *J* = 9.1 Hz, 2H), 3.81 (s, 3H). ^13^C NMR (100 MHz, DMSO-*d6*) *δ* 167.8, 158.2, 148.7, 138.4, 132.9, 126.4, 120.4, 115.4, 114.9, 114.8, 55.6. HRMS (ESI) *m/z*: [M+H]^+^ = 274.0752 (found), 274.0757 (calculated).

*5-Amino-2-(5-amino-1-phenyl-1H-pyrazol-4-yl)-1,3,4-thiadiazole* **2a**: Yield: 65%. m.p.: 131–132 °C. FT-IR ν (cm^−1^): 3413, 3314, 3252, 3118, 3107, 2765, 2633, 1608, 1561, 1505, 1459. ^1^H NMR (400 MHz, DMSO-*d6*) *δ* 7.68 (d, *J* = 0.5 Hz, 1H), 7.61 (d, *J* = 7.8 Hz, 2H), 7.54 (t, *J* = 7.8 Hz, 2H), 7.40 (ddd, *J* = 0.5, 7.8 Hz, 1H), 7.11 (s, 2H), 6.28 (s, 2H). ^13^C NMR (100 MHz, DMSO-*d6*) *δ* 164.8, 151.5, 144.5, 138.6, 138.1, 129.2, 127.0, 122.9, 95.6. HRMS (ESI) *m/z*: [M+H]^+^ = 259.0773 (found), 259.0760 (calculated).

*5-Amino-2-(5-amino-1-(3-chlorophenyl)-1H-pyrazol-4-yl)-1,3,4-thiadiazole* **2b**: Yield: 52%. m.p.: 289–291 °C. FT-IR ν (cm^−1^): 3447, 3263, 3153, 3062, 2760, 2643, 1618, 1596, 1574, 1544, 1505, 1468. ^1^H NMR (400 MHz, DMSO-*d6*) *δ* 7.72 (s, 1H), 7.68 (s, 1H), 7.63–7.54 (m, 2H), 7.46 (d, *J* = 7.8 Hz, 1H), 7.12 (s, 2H), 6.41 (s, 2H). ^13^C NMR (100 MHz, DMSO-*d6*) *δ* 165.0, 151.3, 144.8, 139.4, 139.2, 133.4, 130.9, 126.8, 122.5, 121.3, 95.9. HRMS (ESI) *m/z*: [M+H]^+^ = 293.0369 (found), 293.0371 (calculated).

*5-Amino-2-(5-amino-1-(2,4-dichlorophenyl)-1H-pyrazol-4-yl)-1,3,4-thiadiazole* **2c**: Yield: 61%. m.p.: 165–167 °C. FT-IR ν (cm^−1^): 3428, 3287, 3148, 3071, 2766, 2650, 1611, 1571, 1505, 1470. ^1^H NMR (400 MHz, DMSO-*d6*) *δ* 7.66 (d, *J* = 1.0 Hz, 1H), 7.61-7.55 (m, 2H), 7.06 (s, 2H), 6.23 (s, 2H). ^13^C NMR (100 MHz, DMSO-*d6*) *δ* 164.8, 151.6, 146.2, 139.1, 134.8, 134.3, 133.0, 131.6, 129.9, 128.4, 94.0. HRMS (ESI) *m/z*: [M+H]^+^ = 326.9971 (found), 326.9981 (calculated).

*5-Amino-2-(5-amino-1-(3,5-dichlorophenyl)-1H-pyrazol-4-yl)-1,3,4-thiadiazole* **2d**: Yield: 72%. m.p.: 299–300 °C. FT-IR ν (cm^−1^): 3435, 3322, 3170, 3085, 2668, 1612, 1567, 1504, 1455. ^1^H NMR (400 MHz, DMSO-*d6*) *δ* 7.76 (s, 1H), 7.69 (d, *J* = 1.7 Hz, 2H), 7.64 (t, *J* = 1.7 Hz, 1H), 7.14 (s, 2H), 6.53 (s, 2H). ^13^C NMR (100 MHz, DMSO) *δ* 165.2, 151.2, 145.1, 140.3, 139.8, 134.6, 126.4, 121.3, 96.3. HRMS (ESI) *m/z*: [M+H]^+^ = 326.9997 (found), 326.9981 (calculated).

*5-Amino-2-(5-amino-1-(3,4-dichlorophenyl)-1H-pyrazol-4-yl)-1,3,4-thiadiazole* **2e**: Yield: 69%. m.p.: 258–260 °C. FT-IR ν (cm^−1^): 3436, 3303, 3168, 3091, 2973, 2853, 2637, 1611, 1567, 1506, 1470. ^1^H NMR (400 MHz, DMSO-*d6*) *δ* 7.88 (d, *J* = 2.4 Hz, 1H), 7.79 (d, *J* = 8.7 Hz, 1H), 7.74 (s, 1H), 7.64 (dd, *J* = 8.7 Hz, *J* = 2.4 Hz, 1H), 7.14 (s, 2H), 6.46 (s, 2H). ^13^C NMR (100 MHz, DMSO-*d6*) *δ* 165.2, 151.3, 145.0, 139.5, 138.1, 131.7, 131.2, 129.3, 124.6, 123.1, 96.1. HRMS (ESI) *m/z*: [M+H]^+^ = 326.9987 (found), 326.9981 (calculated).

*5-Amino-2-(5-amino-1-(4-chlorophenyl)-1H-pyrazol-4-yl)-1,3,4-thiadiazole* **2f**: Yield: 65%. m.p.: 238–240 °C. FT-IR ν (cm^−1^): 3389, 3303, 3266, 3209, 3047, 2853, 2712, 2642, 1609, 1570, 1520, 1498, 1475. ^1^H NMR (400 MHz, DMSO-*d6*) *δ* 7.78 (s, 1H), 7.66-7.59 (m, 4H). ^13^C NMR (100 MHz, DMSO-*d6*) *δ* 165.2, 151.6, 144.9, 139.1, 137.2, 131.4, 129.4, 124.9, 96.0. HRMS (ESI) *m/z*: [M+H]^+^ = 293.0365 (found), 293.0371 (calculated).

*5-Amino-2-(5-amino-1-(4-fluorophenyl)-1H-pyrazol-4-yl)-1,3,4-thiadiazole* **2g**: Yield: 56%. m.p.: 251–253 °C. FT-IR ν (cm^−1^): 3458, 3385, 3299, 3100, 3080, 2771, 2645, 1614, 1568, 1508, 1479. ^1^H NMR (400 MHz, DMSO-*d6*) *δ* 7.67 (s, 1H), 7.63 (dd, *J* = 8.8, 4.9 Hz, 2H), 7.38 (t, *J* = 8.8 Hz, 2H), 7.10 (s, 2H), 6.27 (s, 2H). ^13^C NMR (100 MHz, DMSO-*d6*) *δ* 164.9, 160.6 (d, *J* = 244.2 Hz), 151.4, 144.7, 138.5, 134.5 (d, *J* = 2.9 Hz), 125.5 (d, *J* = 8.9 Hz), 116.0 (d, *J* = 22.9 Hz), 95.5. HRMS (ESI) *m/z*: [M+H]^+^ = 277.0653 (found), 277.0666 (calculated).

*5-Amino-2-(5-amino-1-(3-fluorophenyl)-1H-pyrazol-4-yl)-1,3,4-thiadiazole* **2h**: Yield: 26%. m.p.: 285–286 °C. FT-IR ν (cm^−1^): 3412, 3306, 3176, 3081, 2922, 2959, 2643, 1611, 1568, 1522, 1498, 1476. ^1^H NMR (400 MHz, DMSO-*d6*) *δ* 7.72 (s, 1H), 7.61-7.46 (m, 3H), 7.24 (ddd, *J* = 8.7, 2.2 Hz, 1H), 7.12 (s, 2H), 6.42 (s, 2H). ^13^C NMR (100 MHz, DMSO-*d6*) *δ* 165.0, 162.0 (d, *J* = 244.4 Hz), 151.3, 144.7, 139.6 (d, *J* = 10.3 Hz), 139.1, 131.0 (d, *J* = 9.2 Hz), 118.7 (d, *J* = 2.9 Hz), 113.6 (d, *J* = 21.1 Hz), 109.9 (d, *J* = 25.1 Hz), 95.9. HRMS (ESI) *m/z*: [M+H]^+^ = 277.0675 (found), 277.0666 (calculated).

*5-Amino-2-(5-amino-1-(4-bromophenyl)-1H-pyrazol-4-yl)-1,3,4-thiadiazole* **2i**: Yield: 71%. m.p.: 286–287 °C. FT-IR ν (cm^−1^): 3382, 3282, 3167, 3086, 2786, 2730, 2645, 1620, 1573, 1548, 1518, 1490. ^1^H NMR (300 MHz, DMSO-*d6*) *δ* 7.76 (s, 1H), 7.73 (d, *J* = 8.8 Hz, 2H), 7.57 (d, *J* = 8.8 Hz, 2H). ^13^C NMR (75 MHz, DMSO-*d6*) *δ* 165.0, 151.4, 144.6, 138.9, 137.4, 132.1, 124.9, 119.6, 95.8. HRMS (ESI) *m/z*: [M+H]^+^ = 338.9836 (M+2 found), 338.9845 (M+2 calculated).

*5-Amino-2-(5-amino-1-(3-bromophenyl)-1H-pyrazol-4-yl)-1,3,4-thiadiazole* **2j**: Yield: 63%. m.p.: 245–246 °C. FT-IR ν (cm^−1^): 3443, 3301, 3168, 3075, 2932, 2760, 2657, 1614, 1572, 1510, 1469. ^1^H NMR (400 MHz, DMSO-*d6*) *δ* 7.81 (t, *J* = 1.9 Hz, 1H), 7.72 (s, 1H), 7.67–7.64 (m, 1H), 7.61-7.59 (m, 1H), 7.50 (t, *J* = 8.0 Hz, 1H), 7.13 (s, 2H), 6.41 (s, 2H). ^13^C NMR (100 MHz, DMSO-*d6*) *δ* 165.0, 151.3, 144.8, 139.5, 139.2, 131.1, 129.7, 125.3, 121.7, 121.7, 95.9. HRMS (ESI) *m/z*: [M+H]^+^ = 338.9827 (M+2 found), 338.9845 (M+2 calculated).

*5-Amino-2-(5-amino-1-(4-nitrophenyl)-1H-pyrazol-4-yl)-1,3,4-thiadiazole* **2k**: Yield: 45%. m.p.: 321–323 °C. FT-IR ν (cm^−1^): 3467, 3283, 3158, 3115, 2933, 2855, 2639, 1638, 1597, 1575, 1502. ^1^H NMR (400 MHz, DMSO-*d6*) *δ* 8.39 (d, *J* = 9.0 Hz, 2H), 7.96 (d, *J* = 9.0 Hz, 2H), 7.84 (s, 1H), 7.16 (s, 2H), 6.62 (s, 2H). ^13^C NMR (100 MHz, DMSO-*d6*) *δ* 165.2, 151.0, 145.2, 144.9, 143.5, 140.3, 124.9, 124.8, 122.5, 96.6. HRMS (ESI) *m/z*: [M+H]^+^ = 304.0616 (found), 304.0611 (calculated).

*5-Amino-2-(5-amino-1-(4-methoxyphenyl)-1H-pyrazol-4-yl)-1,3,4-thiadiazole* **2l**: Yield: 66%. m.p.: 303–305 °C. FT-IR ν (cm^−1^): 3376, 3306, 3083, 2971, 2903, 2839, 2784, 2688, 1615, 1569, 1510, 1482. ^1^H NMR (400 MHz, DMSO-*d6*) *δ* 7.62 (s, 1H), 7.48 (d, *J* = 8.8 Hz, 2H), 7.10–7.08 (m, 4H), 6.13 (s, 2H), 3.82 (s, 3H). ^13^C NMR (100 MHz, DMSO-*d6*) *δ* 164.7, 158.2, 151.6, 144.5, 138.0, 131.0, 125.0, 114.4, 95.2, 55.3. HRMS (ESI) *m/z*: [M+H]^+^ = 289.0867 (found), 289.0866 (calculated).

### 3.2. In Silico Analysis

The analysis of physicochemical properties was performed using DataWarrior software version 5.5.0. The structure–activity similarity (SAS) map was analyzed using Activity Landscape Plotter, an online open web platform (https://www.difacquim.com/d-tools/ (accessed on 10 December 2023)).

### 3.3. Cell Culture

Vero cell cultures grown in RPMI 1640 medium containing 10% fetal bovine serum (FBS) were cultured at 37 °C in a humidified atmosphere of 5% CO_2_ [82]. The cultures were used for drug screening assays and to obtain culture-derived trypomastigotes of *T. cruzi*.

The primary culture of heart muscle cells was obtained from fetuses of *Swiss webster* mice [83]. The heart was removed and the ventricles were fragmented and subjected to dissociation with trypsin and collagenase type II (Worthington Biochemical Corporation, Lakewood, USA) dissociation solution. Cell monolayers were employed to evaluate drug-induced cardiotoxicity. 

Three-dimensional (3D) cardiac cells were obtained as previously described [84]. Briefly, isolated cardiac muscle cells (2.5 × 10^4^ cells/well) were seeded in 96-well U-bottom plates coated with agarose (1%) to form cell aggregates. Cardiac microtissue (7 days in culture) was employed to determine cardiotoxicity and drug efficacy in more complex systems. All animal-use procedures were approved by the Animal Care and Use Committee of Instituto Oswaldo Cruz (license L017/2022-A2).

### 3.4. Trypanosoma cruzi

Genetically modified *T. cruzi* parasites, of clone Dm28c (TcI) expressing the firefly luciferase enzyme gene (Dm28c-Luc), were kindly provided by Dr. Cristina Henriques [85]. *T. cruzi* Dm28c-Luc was maintained in a Vero cell cultivated in RPMI 1640 medium supplemented with 10% FBS. At 4-day post-infection (dpi), trypomastigotes, released in the cell culture supernatant, were harvested, followed by sedimentation (4000 rpm/20 min/4 °C) and quantification in a Neubauer chamber [38]. Isolated trypomastigotes were used in the phenotypic screening assays as well as the antiparasitic activity in 3D cardiac microtissues, recovery (washout), and drug combination assays.

### 3.5. Toxicity in Mammalian Cells

Vero cells (1.5 ×10^4^ cells/well), cultivated for 24 h at 37 °C in white 96-well plates with clear bottoms, were used to assess the toxicity of the pyrazole-thiadiazole derivatives (series 1 and 2). After washing, the cells were incubated for 72 h at 37 °C with different drug concentrations (500–1.95 μM). Cell viability was measured by adding 20 μL/well of CellTiter Glo reagent (Promega Corporation, Madison, WI, USA). This cell viability kit generates a luminescent signal proportional to the amount of ATP. The luminescence readout was performed using the Glomax Microplate Reader (Promega Corporation, Madison, WI, USA). Thus, the 50% cytotoxicity concentration (CC_50_), which reduces the cell viability by 50% (CC_50_), was calculated with Prism GraphPad software (version 8.2.1) [37,38].

Additionally, the cytotoxicity of the most active compounds was performed in cardiac monolayers (2D) and 3D spheroids models. The 2D cardiac cultures (5 × 10^4^ cells/well) and 3D cardiac microtissues (2.5 × 10^4^ cells/well) were incubated for 72 h at 37 °C with pyrazole-thiadiazole candidates followed by ATP detection by CellTiter-Glo [86]. A minimum of 3 independent assays were performed in duplicate.

### 3.6. Anti-T. cruzi Activity In Vitro

Drug screening was performed against clinically relevant forms of *T. cruzi* (amastigotes and trypomastigotes). Trypomastigotes, Dm28c-Luc (1 × 10^6^ parasites/well), were added to 96-well plates and incubated with pyrazole-thiadiazole derivatives or Bz (100–0.41 µM). After 24 h of treatment (37 °C), the viability of the parasites was analyzed by adding a *D*-Luciferin solution (300 µg/mL) and luminescence was measured in the Glomax microplate reader.

The activity of pyrazole-thiadiazole derivatives against intracellular amastigotes was evaluated in 24 h-*T. cruzi* infected Vero cell cultures (1.5 × 10^4^ cells/well) at a 10:1 ratio. The cultures were washed and incubated with serial dilutions of the derivatives or Bz (100–0.41 µM). Drug treatment was carried out for 72 h at 37 °C and the parasite viability was determined by luminescence readout after *D*-Luciferin substrate addition (300 µg/mL). The reading was performed in the Glomax reader. The cell cultures incubated with the culture medium containing dimethylsulphoxide (DMSO; concentration ≤ 1%) were used as the positive control. The concentrations required to inhibit parasite viability by 50% (IC_50_) and 90% (IC_90_) were calculated using Prism GraphPad software (version 8.2.1). The selectivity index (SI), which measures the window between cytotoxicity (CC_50_) and anti-*T. cruzi* activity (IC_50_), was calculated as CC_50_/IC_50_. A minimum of 3 independent assays in duplicate were performed [38].

### 3.7. Antiparasitic Activity in 3D Cardiac Microtissues

The efficacy of the most active pyrazole-thiadiazole derivatives was assessed in the *T. cruzi*-infected 3D cardiac microtissue model. Cardiac spheroids (2.5 × 10^4^ cells/well), formed after 7 days of cultivation in a U-bottom plate, were infected for 24 h with trypomastigote forms of *T. cruzi* (Dm28c-Luc) at a ratio of 20:1 parasite-host cell. After washing, the drugs were added at concentrations of IC_90_, two times IC_90_, and 100 µM, respecting the CC_20_ value of cardiac spheroids as the maximum concentration. Bz was added at 100 µM. Luminescence measurements, which reveal the luciferase activity of the viable parasites, were performed after the addition of *D*-luciferin substrate (300 µg/mL). The luminescent signal was read in the Glomax reader and the results were represented in arbitrary luminescence units (ALU). The fluorescence microscopy images were also acquired from untreated and drug-treated infected 3D microtissues. Three-dimensional cultures were fixed with 4% paraformaldehyde in PBS and labeled with 4′,6-diamidino-2-phenylindole (DAPI). Images were acquired using a Zeiss Axio Imager M2 fluorescence microscope using Axio vision software (version 4.8) [37].

### 3.8. Washout Assay

*T. cruzi*-infected Vero cell cultures were subjected to experimental treatment with the most active compounds at concentrations 3.5 to 13 times the IC_90_ for 10 days at 37 °C. The culture medium of the untreated and treated infected cultures was replaced every 3 or 4 days by a fresh medium with or without the pyrazole-thiadiazole derivatives. The presence of free viable parasites in the culture supernatant was monitored at each medium change by adding luciferin (300 µg/mL) followed by readout on a Glomax microplate reader. At the endpoint, culture supernatants and cell monolayers were evaluated for viable parasites. Positive and negative controls were performed using Bz (150 µM) and DMSO (≤1%), respectively. A minimum of 3 independent experimental runs were performed with 12 replicates.

### 3.9. Scanning Electron Microscopy

Trypomastigotes (Dm28c-Luc), untreated or treated for 24 h at 37 °C with IC_50_ and IC_75_ values of the most active compounds, were washed in PBS and fixed with glutaraldehyde (2.5%) (Sigma-Aldrich, St. Louis, MO, USA) in 0.1 M sodium cacodylate buffer containing 3.5% sucrose, pH 7.2. After washing, the parasites were adhered to glass coverslips coated with poly-*L*-lysine. Then, the cells were post-fixed with 1% osmium tetroxide (OsO_4_) in 0.1 M sodium cacodylate buffer containing 3.5% sucrose, pH 7.2. Subsequently, the samples were dehydrated in an increasing ethanol series (30, 50, 70, 90, and 100%–10 min each) and dried by the critical point method with CO_2_. Samples were mounted onto metal stubs and sputter-coated with gold for analysis under a JEOL scanning electron microscope (JEOL-JSM-6390-LV). 

### 3.10. In Vitro Drug Combination

The nature of interactions between Bz and the most active pyrazole-thiadiazole derivatives was evaluated by using an in vitro drug combination assay. Vero cell cultures infected with *T. cruzi* (Dm28c-Luc) were subjected to in vitro combination treatment for 72 h at 37 °C with selected candidates and Bz using a fixed ratio method [87]. The experimental design consisted of six concentration combinations (5:0, 4:1, 3:2, 2:3, 1:4, and 0:5) of each drug and Bz at a 1:3 ratio. The maximum compound concentrations were determined by ensuring that their IC_50_ values reached the fourth dilution in the series. Then, the serial dilutions were transferred to cultures infected with *T. cruzi* (Dm28c-Luc), followed by 72 h of experimental treatment. For each ratio, the relative IC_50_ of each compound was calculated separately. The fractional inhibitory concentration index (FICI) was calculated as follows: IC_50_ of the combination/IC_50_ of the compound alone. The sum of FICI (ΣFICI) was determined as composite FICI A + composite FICI B. xΣFICI, an average of sums of FICI, was used to classify the interaction as synergistic xΣFICI ≤ 0.5, additive xΣFICI > 0.5–4, and antagonistic for xΣFICI > 4. The isobologram graph was constructed by tracing the FICI for each compound proportion [38,88].

### 3.11. Statistical Analysis

Data represent at least three independent experiments, with the values expressed in mean ± standard deviation (SD). Statistical analysis was performed with the Prism GraphPad 8.0 software. Data were analyzed using one-way ANOVA followed by the Kruskal–Wallis test. A *p*-value of 0.05 or lower was considered statistically significant.

## 4. Conclusions

The research presented in this work focuses on the bioactivity and selectivity of newly synthesized pyrazole-thiadiazole derivatives against *T. cruzi*. The study assessed the efficacy of the most active compounds (**1c** and **2k**) using robust preclinical cell-based models to enhance the in vitro to in vivo translatability. Derivative **2k** exhibited significant activity in 3D cardiac microtissues, leading to a substantial reduction in parasite load. Furthermore, derivative **2k** demonstrated comparable efficacy to Bz in controlling parasite recrudescence and displayed an additive effect in Bz combination assay. These findings suggest the potential for structural optimization of the **2k** derivative to enhance its biological activity against *T. cruzi*.

## Figures and Tables

**Table 1:** Biological activity of pyrazole-thiadiazole derivatives against *Trypanosoma cruzi*.

	Anti-*T. cruzi* Activity (mean ± SD µM)							Cytotoxicity (Mean ± SD µM)
	Trypomastigotes			Intracellular Amastigotes				Vero Cells
Compounds	IC_50_	IC_90_	SI	IC_50_	IC_90_	SI	pIC50	CC_50_
**1a**	>100	>100	>5	68.63 ± 1.03	>70	>7.49	4.16	>500
**1b**	>100	>100	>5	68.11 ± 2.99	>70	>7.73	4.17	>500
**1c**	21.71 ± 2.94	>100	>23.03	13.54 ± 4.47	42.27 ± 3.47	>32.96	4.87	>500
**1d**	>100	>100	>5	58.66 ± 12.29	>70	>8.29	4.23	>500
**1e**	>100	>100	>5	18.75 ± 2.28	40.37 ± 13.31	>25.64	4.73	>500
**1f**	>100	>100	>5	>70	>70	>7.14	<4.15	>500
**1g**	>100	>100	>5	>70	>70	>7.14	<4.15	>500
**1h**	>100	>100	>5	66.85 ± 3.37	>70	>7.86	4.17	>500
**1i**	>100	>100	>5	28.41 ± 1.77	>70	>17.53	4.55	>500
**1j**	>100	>100	>5	>70	>70	>7.14	<4.15	>500
**1k**	>100	>100	>5	58.33 ± 12.83	>70	>8.16	4.23	>500
**1l**	53.03 ± 4.44	>100	>9.43	24.46 ± 4.21	>70	>19.08	4.61	>500
**2a**	>100	>100	>5	>70	>70	>7.14	<4.15	>500
**2b**	>100	>100	>5	>70	>70	>7.14	<4.15	>500
**2c**	>100	>100	>5	>70	>70	>7.14	<4.15	>500
**2d**	>100	>100	>5	>70	>70	>7.14	<4.15	>500
**2e**	72.62 ± 6.76	>100	>6.88	33.64 ± 2.16	58.83 ± 8.26	>13.73	4.47	>500
**2f**	>100	>100	>5	>70	>70	>7.14	<4.15	>500
**2g**	>100	>100	>5	>70	>70	>7.14	<4.15	>500
**2h**	>100	>100	>5	>70	>70	>7.14	<4.15	>500
**2i**	>100	>100	>5	68.73 ± 0.05	>70	>7.58	4.16	>500
**2j**	>100	>100	>5	>70	>70	>7.14	<4.15	>500
**2k**	>100	>100	>5	10.37 ± 1.21	23.19 ± 4.23	>33.87	4.98	>500
**2l**	>100	>100	>100	>70	>70	>7.14	<4.15	>500
**Bz**	12.82 ± 2.66	>100	>38.88	3.61 ± 1.25	11.53 ± 4.17	>158.23	5.44	>500

The IC_50_ and IC_90_ mean values from three independent experiments ± standard deviation (SD); IC_50_: Concentration that inhibits parasite viability by 50%; CC_50_: Concentration that reduces the viability of Vero cells by 50%; Selectivity index (SI) = CC_50_ of Vero cells/IC_50_ of trypomastigotes or intracellular amastigotes of *T. cruzi*.

**Table 2:** Cardiac toxicity of selected pyrazole-thiadiazole candidates.

Compounds	Toxicity CC_50_ (Mean ± SD µM)	
	2D Culture	3D Culture
**1c**	>500	>500
**2k**	191.20 ± 21.0	>500
**Bz**	>500	>500

CC_50_ mean values from three independent experiments ± standard deviation (SD); CC_50_: Concentration that reduces the viability of cardiac muscle cells by 50%.

## Data Availability

The authors confirm that the data supporting the findings of this study are available within the article [and/or its Supplementary Materials].

## Supplementary Materials

The following supporting information can be downloaded at: https://www.mdpi.com/article/10.3390/molecules29153544/s1: Figure S1–S3—Z-axis images of cardiac spheroids; Figure S4 NMR spectra of the compounds.

## References

[1] Alevi K.C.C., de Oliveira J., Rocha D.S.R., Galvão C. (2021). Trends in taxonomy of Chagas disease vectors (Hemiptera, Reduviidae, Triatominae): From Linnaean to integrative taxonomy. Pathogens.

[2] Vieira C.B., Praça Y.R., Bentes K.L.D.S., Santiago P.B., Silva S.M.M., Silva G.D.S., Motta F.N., Bastos I.M.D., de Santana J.M., de Araújo C.N. (2018). Triatomines: Trypanosomatids, bacteria, and viruses potential vectors?. Front. Cell. Infect. Microbiol..

[3] Franco-Paredes C., Villamil-Gómez W.E., Schultz J., Henao-Martínez F., Parra-Henao G., Rassi A., Rodríguez-Morales A.J., Suarez J.A. (2020). A deadly feast: Elucidating the burden of orally acquired acute Chagas disease in Latin America—Public health and travel medicine importance. Travel Med. Infect. Dis..

[4] World Health Organization Chagas Disease (also Known as American Trypanosomiasis). https://www.who.int/news-room/fact-sheets/detail/chagas-disease-(American-trypanosomiasis).

[5] World Health Organization Ending the Neglect to Attain the Sustainable Development Goals: A Road Map for Neglected Tropical Diseases 2021–2030. https://apps.who.int/iris/handle/10665/338565.

[6] Pan American Health Organization Less than 10% of People with Chagas Receive a Diagnosis. https://www.paho.org/en/news/13-4-2023-less-10-people-chagas-receive-diagnosis.

[7] Pérez-Molina J.A., Molina I. (2018). Chagas disease. Lancet Infect. Dis..

[8] Rassi A., Marin-Neto J.A. (2010). Chagas disease. Lancet Infect. Dis..

[9] Keegan R., Yeung C., Baranchuk A. (2020). Sudden cardiac death risk stratification and prevention in Chagas disease: A non-systematic review of the literature. Arrhythm. Electrophysiol..

[10] De Sousa A.S., Vermeij D., Ramos A.N., Luquetti A.O. (2024). Chagas disease. Lancet.

[11] Morillo C.A., Marin-Neto J.A., Avezum A., Sosa-Estani S., Rassi A., Rosas F., Villena E., Quiroz R., Bonilla R., Britto C. (2015). Randomized trial of benznidazole for chronic Chagas’ cardiomyopathy. N. Engl. J. Med..

[12] Marin-Neto J.A., Rassi A. (2022). The challenge of risk assessment in the riddle of Chagas heart disease. Mem. Inst. Oswaldo Cruz..

[13] Bosch-Nicolau P., Fernández M.L., Sulleiro E., Villar J.C., Perez-Molina J.A., Correa-Oliveira R., Sosa-Estani S., Sánchez-Montalvá A., Del Carmen Bangher M., Moreira O.C. (2024). Efficacy of three benznidazole dosing strategies for adults living with chronic Chagas disease (MULTIBENZ): An international, randomised, double-blind, phase 2b trial. Lancet.

[14] Molina I., Prat J.G., Salvador F., Treviño B., Sulleiro E., Serre N., Pou D., Roure S., Cabezos J., Valerio L. (2014). Randomized trial of posaconazole and benznidazole for chronic Chagas’ disease. N. Engl. J. Med..

[15] Morillo C.A., Waskin H., Sosa-Estani S., Del Carmen Bangher M., Cuneo C., Milesi R., Mallagray M., Apt W., Beloscar J., Gascon J. (2017). Benznidazole and posaconazole in eliminating parasites in asymptomatic *T. cruzi* carriers: The STOP-CHAGAS Trial. J. Am. Coll. Cardiol..

[16] Pinazo M.-J., Forsyth C., Losada I., Esteban E.T., García-Rodríguez M., Villegas M.L., Molina I., Crespillo-Andújar C., Gállego M., Ballart C. (2024). Efficacy and safety of fexinidazole for treatment of chronic indeterminate Chagas disease (FEXI-12): A multicentre, randomised, double-blind, phase 2 trial. Lancet Infect. Dis..

[17] Torrico F., Gascón J., Barreira F., Blum B., Almeida I.C., Alonso-Vega C., Barboza T., Bilbe G., Correia E., Garcia W. (2021). New regimens of benznidazole monotherapy and in combination with fosravuconazole for treatment of Chagas disease (BENDITA): A phase 2, double-blind, randomised trial. Lancet Infect. Dis..

[18] Torrico F., Gascón J., Ortiz L., Pinto J., Rojas G., Palacios A., Barreira F., Blum B., Schijman A.G., Vaillant M. (2022). A phase-2, randomized, multicenter, placebo-controlled, proof-of-concept trial of oral fexinidazole in adults with chronic indeterminate Chagas disease. Clin. Infect. Dis..

[19] Gabaldón-Figueira J.C., Martinez-Peinado N., Escabia E., Ros-Lucas A., Chatelain E., Scandale I., Gascon J., Pinazo M.J., Alonso-Padilla J. (2023). State-of-the-art in the drug discovery pathway for Chagas disease: A framework for drug development and target validation. Res. Rep. Trop. Med..

[20] Pfarr K.M., Krome A.K., Al-Obaidi I., Batchelor H., Vaillant M., Hoerauf A., Opoku N.O., Kuesel A.C. (2023). The pipeline for drugs for control and elimination of neglected tropical diseases: 1. Anti-infective drugs for regulatory registration. Parasit. Vectors.

[21] Abbasi Shiran J., Ghanbari M., Mohammadnejadi E., Razzaghi-Asl N. (2023). Structural insight into privileged heterocycles as anti-*Trypanosoma cruzi* and *brucei* agents. Curr. Top. Med. Chem..

[22] Anthwal T., Paliwal S., Nain S. (2022). Diverse biological activities of 1,3,4-thiadiazole scaffold. Chemistry.

[23] Kaur P., Arora V. (2023). Pyrazole as an anti-microbial scaffold: A comprehensive review. Mini Rev. Org. Chem..

[24] Reviriego F., Olmo F., Navarro P., Marín C., Ramírez-Macías I., García-España E., Albelda M.T., Gutiérrez-Sánchez R., Sánchez-Moreno M., Arán V.J. (2017). Simple dialkyl pyrazole-3,5-dicarboxylates show in vitro and in vivo activity against disease-causing trypanosomatids. Parasitology.

[25] Hassanzadeh F., Jafari E., Saeedi M., Saberi S. (2020). Synthesis and evaluation of thiadiazole-based antileishmanial agents. J. Rep. Pharm. Sci..

[26] Bennani F.E., Doudach L., Cherrah Y., Ramli Y., Karrouchi K., Faouzi M.E.A. (2020). Overview of recent developments of pyrazole derivatives as an anticancer agent in different cell line. Bioorg. Chem..

[27] Chaudhari P.J., Bari S.B., Surana S.J., Shirkhedkar A.A., Bonde A.G., Khadse S.C., Ugale V.G., Nagar A.A., Cheke R.S. (2022). discovery and anticancer activity of novel 1,3,4-thiadiazole- and aziridine-based indolin-2-ones via in silico design followed by supramolecular green synthesis. ACS Omega.

[28] Mantzanidou M., Pontiki E., Hadjipavlou-Litina D. (2021). Pyrazoles and pyrazolines as anti-inflammatory agents. Molecules.

[29] Koval A., Lozynskyi A., Shtrygol S., Lesyk R. (2022). An overview on 1,2,4-triazole and 1,3,4-thiadiazole derivatives as potential anesthesic and anti-inflammatory agents. ScienceRise Pharm. Sci..

[30] Varghese S., Rahmani R., Russell S., Deora G.S., Ferrins L., Toynton A., Jones A., Sykes M., Kessler A., Eufrasio A. (2019). Discovery of potent N-ethylurea pyrazole derivatives as dual inhibitors of *Trypanosoma brucei* and *Trypanosoma cruzi*. ACS Med. Chem. Lett..

[31] Salvador R.R.S., Bello M.L., Barreto I.R.L., Vera M.A.F., Muri E.M.F., Albuquerque S., Dias L.R.S. (2016). New carbohydrazide derivatives of 1*H*-pyrazolo[3,4-b]pyridine and trypanocidal activity. An. Acad. Bras. Cienc..

[32] Linciano P., Dawson A., Pöhner I., Costa D.M., Sá M.S., Cordeiro-da-Silva A., Luciani R., Gul S., Witt G., Ellinger B. (2017). Exploiting the 2-Amino-1,3,4-Thiadiazole Scaffold To Inhibit *Trypanosoma brucei* Pteridine Reductase in Support of Early-Stage Drug Discovery. ACS Omega.

[33] Freitas R.H., Barbosa J.M., Bernardino P., Sueth-Santiago V., Wardell S.M., Wardell J.L., Decoté-Ricardo D., Melo T.G., da Silva E.F., Salomão K. (2020). Synthesis and trypanocidal activity of novel pyridinyl-1,3,4-thiadiazole derivatives. Biomed. Pharmacother..

[34] Martins S.C., Lazarin-Bidóia D., Desoti V.C., Falzirolli H., da Silva C.C., Ueda-Nakamura T., Silva S.O., Nakamura C.V. (2016). 1,3,4-Thiadiazole derivatives of R–(+)–limonene benzaldehyde-thiosemicarbazones cause death in *Trypanosoma cruzi* through oxidative stress. Microbes Infect..

[35] Camargo J.d.N.A., Pianoski K.E., dos Santos M.G., Lazarin-Bidóia D., Volpato H., Moura S., Nakamura C.V., Rosa F.A. (2020). Antiparasitic Behavior of Trifluoromethylated Pyrazole 2-Amino-1,3, 4-thiadiazole Hybridsand Their Analogues: Synthesis and Structure-Activity Relationship. Front. Pharmacol..

[36] Monteiro M.E., Lechuga G., Lara L.S., Souto B.A., Viganó M.G., Bourguignon S.C., Calvet C.M., Oliveira F.O.R., Alves C.R., Souza-Silva F. (2019). Synthesis, structure-activity relationship and trypanocidal activity of pyrazole-imidazoline and new pyrazole-tetrahydropyrimidine hybrids as promising chemotherapeutic agents for Chagas disease. Eur. J. Med. Chem..

[37] Orlando L.M.R., Lechuga G.C., Lara L.d.S., Ferreira B.S., Pereira C.N., Silva R.C., Dos Santos M.S., Pereira M.C.S. (2021). Structural optimization and biological activity of pyrazole derivatives: Virtual computational analysis, recovery assay and 3d culture model as potential predictive tools of effectiveness against *Trypanosoma cruzi*. Molecules.

[38] Lara L.d.S., Lechuga G.C., Orlando L.M.R., Ferreira B.S., Souto B.A., dos Santos M.S., Pereira M.C.S. (2022). Bioactivity of novel pyrazole-thiazolines scaffolds against *Trypanosoma cruzi*: Computational approaches and 3d spheroid model on drug discovery for Chagas disease. Pharmaceutics.

[39] Rosa G.S., Souto B.A., Pereira C.N., Teixeira B.C., Dos Santos M.S. (2019). A convenient synthesis of pyrazole-imidazoline derivatives by microwave irradiation. J. Heterocycl. Chem..

[40] Lourenço A.L.P.G., Vegi P.F., Faria J.V., Pinto G.S.P., Dos Santos M.S., Sathler P.C., Saito M.S., Santana M., Dutra T.P.P., Rodrigues C.R. (2019). Pyrazolyl-tetrazoles and imidazolyl-pyrazoles as potential anticoagulants and their integrated multiplex analysis virtual screening. J. Braz. Chem. Soc..

[41] Ishankhodzhaeva M.M., Kadyrova S.A., Surazhskaya M.D., Parpiev N.A., Koz P.A. (2001). Crystalline and Molecular Structure of 2-amino-5-phenyl-1,3,4-thiadiazole. Russ. J. Org. Chem..

[42] Sun D., Gao W., Hu H., Zhou S. (2022). Why 90% of clinical drug development fails and how to improve it?. Acta Pharm. Sin. B.

[43] Don R., Ioset J.R. (2014). Screening strategies to identify new chemical diversity for drug development to treat kinetoplastid infections. Parasitology.

[44] Katsuno K., Burrows J.N., Duncan K., Hooft van Huijsduijnen R., Kaneko T., Kita K., Mowbray C.E., Schmatz D., Warner P., Slingsby B.T. (2015). Hit and lead criteria in drug discovery for infectious diseases of the developing world. Nat. Rev. Drug Discov..

[45] Chiodi D., Ishihara Y. (2023). “Magic Chloro”: Profound effects of the chlorine atom in drug discovery. J. Med. Chem..

[46] Egan T.J., Hunter R., Kaschula C.H., Marques H.M., Misplon A., Walden J. (2000). Structure−function relationships in aminoquinolines: Effect of amino and chloro groups on quinoline−hematin complex formation, inhibition of β-hematin formation, and antiplasmodial activity. J. Med. Chem..

[47] Saenz-Garcia J.L., Borges B.S., Souza-Melo N., Machado L.V., Miranda J.S., Pacheco-Lugo L.A., Moretti N.S., Wheleer R., Soares Medeiros L.C., DaRocha W.D. (2022). Trypanin disruption affects the motility and infectivity of the protozoan *Trypanosoma cruzi*. Front. Cell. Infect. Microbiol..

[48] Ralston K.S., Hill K.L. (2006). Trypanin, a component of the flagellar dynein regulatory complex, is essential in the bloodstream form African trypanosomes. PLoS Pathog..

[49] Kabututu Z.P., Thayer M., Melehani J.H., Hill K.L. (2010). CMF70 Is a subunit of the dynein regulatory complex. J. Cell Sci..

[50] Ralston K.S., Lerner A.G., Diener D.R., Hill K.L. (2006). Flagellar motility contributes to cytokinesis in *Trypanosoma brucei* and is modulated by an evolutionarily conserved dynein regulatory system. Eukaryot. Cell.

[51] Cooper R., de Jesus A.R., Cross G.A. (1993). Deletion of an immunodominant Trypanosoma cruzi surface glycoprotein disrupts flagel-lum-cell adhesion. J. Cell Biol..

[52] de Jesus A.R., Cooper R., Espinosa M., Gomes J.E., Garcia E.S., Paul S., Cross G.A. (1993). Gene deletion suggests a role for *Trypanosoma cruzi* surface glycoprotein GP72 in the insect and mammalian stages of the life cycle. J. Cell Sci..

[53] Kohl L., Robinson D., Bastin P. (2003). Novel roles for the flagellum in cell morphogenesis and cytokinesis of trypanosomes. EMBO J..

[54] Vaughan S., Kohl L., Ngai I., Wheeler R.J., Gull K. (2008). A repetitive protein essential for the flagellum attachment zone filament structure and function in *Trypanosoma brucei*. Protist.

[55] Kelly F.D., Sanchez M.A., Landfear S.M. (2020). Touching the surface: Diverse roles for the flagellar membrane in kinetoplastid parasites. Microbiol. Mol. Biol. Rev..

[56] Galetovic A., Souza R.T., Santos M.R.M., Cordero E.M., Bastos I.M.D., Santana J.M., Ruiz J.C., Lima F.M., Marini M.M., Mortara R.A. (2011). The repetitive cytoskeletal protein H49 of *Trypanosoma cruzi* is a calpain-like protein located at the flagellum attachment zone. PLoS ONE.

[57] Fairlamb A.H., Bowman I.B.R. (1980). Uptake of trypanocidal drug suramin by bloodstream forms of *Trypanosoma brucei* and its effects on respiration and growth rate in vivo. Mol. Biochem. Parasitol..

[58] Voogd T.E., Vansterkenburg E.L., Wilting J., Janssen L.H. (1993). Recent research on the biological activity of suramin. Pharmacol. Rev..

[59] Bisaggio D.F., Campanati L., Pinto R.C., Souto-Padron T. (2006). Effect of suramin on trypomastigote forms of *Trypanosoma cruzi*: Changes on cell motility and on the ultrastructure of the flagellum-cell body attachment region. Acta Trop..

[60] Mamoshina P., Rodriguez B., Bueno-Orovio A. (2021). Toward a broader view of mechanisms of drug cardiotoxicity. Cell Rep. Med..

[61] Xu F., Li X., Yu X., Li Q., Li W., Xiao X. (2022). Effects of Different doses of doxorubicin on H9C2 cells. J. Biosci. Med..

[62] Fiuza L.F.d.A., Batista D.G.J., Girão R.D., Hulpia F., Finamore-Araújo P., Aldfer M.M., Elmahallawy E.K., De Koning H.P., Moreira O., Van Calenbergh S. (2022). Phenotypic evaluation of nucleoside analogs against *Trypanosoma cruzi* infection: In vitro and in vivo approaches. Molecules.

[63] Soltantabar P., Calubaquib E.L., Mostafavi E., Ghazavi A., Stefan M.C. (2021). Heart/liver-on-a-chip as a model for the evaluation of cardiotoxicity induced by chemotherapies. Organs-on-a-Chip.

[64] Zuppinger C. (2019). 3D cardiac cell culture: A critical review of current technologies and applications. Front. Cardiovasc. Med..

[65] Tadano K., Miyagawa S., Takeda M., Tsukamoto Y., Kazusa K., Takamatsu K., Akashi M., Sawa Y. (2021). Cardiotoxicity assessment using 3D vascularized cardiac tissue consisting of human iPSC-derived cardiomyocytes and fibroblasts. Mol. Ther. Methods Clin. Dev..

[66] Arai K., Kitsuka T., Nakayama K. (2021). Scaffold-based and scaffold-free cardiac constructs for drug testing. Biofabrication.

[67] Berrouet C., Dorilas N., Rejniak K.A., Tuncer N. (2020). Comparison of drug inhibitory effects (IC50) in monolayer and spheroid cultures. Bull. Math. Biol..

[68] Melissaridou S., Wiechec E., Magan M., Jain M.V., Chung M.K., Farnebo L., Roberg K. (2019). The effect of 2D and 3D cell cultures on treatment response, EMT profile, and stem cell features in head and neck cancer. Cancer Cell Int..

[69] Keeffe A.O., Hale C., Cotton J.A., Yardley V., Gupta K., Ananthanarayanan A., Murdan S., Croft S.L. (2020). Novel 2D and 3D assays to determine the activity of anti-leishmanial drugs. Microorganisms..

[70] Khandelwal A., Arez F., Alves P.M., Badolo L., Brito C., Fischli C., Fontinha D., Oeuvray C., Prudêncio M., Rottmann M. (2022). Translation of liver stage activity of M5717, a *Plasmodium* elongation factor 2 inhibitor: From bench to bedside. Malar. J..

[71] Rodriguez M.E., Tekiel V., Campo V.A. (2022). In vitro evaluation of Resveratrol as a potential pre-exposure prophylactic drug against *Trypanosoma cruzi* infection. Infect. Dis. Poverty.

[72] de Almeida Fiuza L.F., Batista D.D.G.J., Nunes D.F., Moreira O.C., Cascabulho C., Soeiro M.N.C. (2021). Benznidazole modulates the release of inflammatory mediators by cardiac spheroids infected with *Trypanosoma cruzi*. Exp. Parasitol..

[73] Sykes M.L., Kennedy E.K., Read K.D., Kaiser M., Avery V.M. (2022). Temporal and wash-out studies identify medicines for malaria venture pathogen box compound with fast-acting activity against both *Trypanosoma cruzi* and *Trypanosoma brucei*. Microorganisms.

[74] MacLean L.M., Thomas J., Lewis M.D., Cotillo I., Gray D.W., De Rycker M. (2018). Development of *Trypanosoma cruzi* in vitro assays to identify compounds suitable for progression in Chagas’ disease drug discovery. PLoS Negl. Trop. Dis..

[75] Cardoso-Santos C., Ferreira de Almeida Fiuza L., França da Silva C., Mazzeti A.L., Donola Girão R., Melo de Oliveira G., da Gama J.B.D., Cruz M.O., Lins S.G.N., Maes L. (2021). 7-Aryl-7-deazapurine 30-deoxyribonucleoside derivative as a novel lead for Chagas’ disease therapy: In vitro and in vivo pharmacology. JAC Antimicrob. Resist..

[76] Mazzeti A.L., Capelari-Oliveira P., Bahia M.T., Mosqueira V.C.F. (2021). Review on experimental treatment strategies against *Trypanosoma cruzi*. J. Exp. Pharmacol..

[77] Porta E.O.J., Kalesh K., Steel P.G. (2023). Navigating drug repurposing for Chagas disease: Advances, challenges, and opportunities. Front. Pharmacol..

[78] Pandey R.P., Nascimento M.S., Franco C.H., Bortoluci K., Silva M.N., Zingales B., Gibaldi D., Castaño Barrios L., Lannes-Vieira J., Cariste L.M. (2022). Drug repurposing in Chagas disease: Chloroquine potentiates benznidazole activity against *Trypanosoma cruzi* in vitro and in vivo. Antimicrob. Agents Chemother..

[79] Machado Y.A., Bahia M.T., Caldas I.S., Mazzeti A.L., Novaes R.D., Boas R.V.B., Santos L.J.S., Martins-Filho O.A., Marques M.J., Diniz L.F. (2020). Amlodipine increases the therapeutic potential of ravuconazole upon *Trypanosoma cruzi* infection, *Antimicrob*. Agents Chemother..

[80] Mazzeti A., Gonçalves K., Mota S., Pereira D., Diniz L., Bahia M. (2021). Combination therapy using nitro compounds improves the efficacy of experimental Chagas disease treatment. Parasitology.

[81] de Araújo J.S., França S.C., Batista D.D.G.J., Nefertiti A., Fiuza L.F.D.A., Fonseca-Berzal C.R., Bernardino S.P., Batista M.M., Sijm M., Kalejaiye T.D. (2020). Efficacy of novel pyrazolone phosphodiesterase inhibitors in experimental mouse models of *Trypanosoma cruzi*. Antimicrob. Agents Chemother..

[82] Ammerman N.C., Beier-Sexton M., Azad A.F. (2008). Growth and maintenance of Vero cell lines. Curr. Protoc. Microbiol..

[83] Meirelles M.N., de Araujo-Jorge T.C., Miranda C.F., de Souza W., Barbosa H.S. (1986). Interaction of *Trypanosoma cruzi* with heart muscle cells: Ultrastructural and cytochemical analysis of endocytic vacuole formation and effect upon myogenesis in vitro. Eur. J. Cell Biol..

[84] Garzoni L.R., Adesse D., Soares M.J., Rossi M.I., Borojevic R., de Meirelles M.d.N. (2008). Fibrosis and hypertrophy induced by *Trypanosoma cruzi* in a three-dimensional cardiomyocyte-culture system. J. Infect. Dis..

[85] Henriques C., Henriques-Pons A., Meuser-Batista M., Ribeiro A.S., de Souza W. (2014). In vivo imaging of mice infected with bioluminescent *Trypanosoma cruzi* unveils novel sites of infection. Parasit. Vectors.

[86] Orlando L.M.R., Lara L.d.S., Lechuga G.C., Rodrigues G.C., Pandoli O.G., de Sá D.S., Pereira M.C.S. (2023). Antitrypanosomal activity of 1,2,3-triazole-based hybrids evaluated using in vitro preclinical translational models. Biology.

[87] Fivelman Q.L., Adagu I.S., Warhurst D.C. (2004). Modified fixed-ratio isobologram method for studying in vitro interactions between atovaquone and proguanil or dihydroartemisinin against drug-resistant strains of *Plasmodium falciparum*. Antimicrob. Agents. Chem..

[88] Odds F.C. (2003). Synergy, antagonism, and what the chequerboard puts between them. J. Antimicrob. Chemother..

