# Supplementary material for: Synthesis and Anti-Trypanosoma cruzi Activity of New Pyrazole-Thiadiazole Scaffolds
Source: Molecules. 2024 Jul 27;29(15):3544. doi: 10.3390/molecules29153544 (PMC11314410; doi:10.3390/molecules29153544)

Supporting information

Synthesis and anti-*Trypanosoma cruzi* activity of new pyrazole-thiadiazole scaffolds

Thamyris Pérez de Souza [a], Lorraine Martins Rocha Orlando [a], Leonardo Silva Lara [a], Vitória Barbosa Paes [a], Lucas Penha Dutra [b], Maurício Silva dos Santos [b], Mirian Claudia de Souza Pereira [a,*]

[a] Laboratório de Ultraestrutura Celular, Instituto Oswaldo Cruz, Fiocruz. Av. Brasil 4365, Rio de Janeiro, RJ, 21040-900, Brazil.
[b] Laboratório de Síntese de Sistemas Heterocíclicos (LaSSH), Instituto de Física e Química (IFQ), Universidade Federal de Itajubá, Av. BPS 1303, Pinheirinho, 37500-903, Itajubá, MG, Brazil.
* Corresponding author: E-mail address: mirian@ioc.fiocruz.br (M.C.S. Pereira); Tel.: +55-21-2562-1023; Fax: +55-21-2562-1019

Table of contents

Z-axis images of cardiac spheroids infected by *T. cruzi* (untreated) .... Figure S1

Z-axis images of cardiac spheroids infected and treated with 2k .... Figure S2

Z-axis images of cardiac spheroids infected and treated with Bz.................... Figure S3

NMR spectra of the compounds .... Figure S4

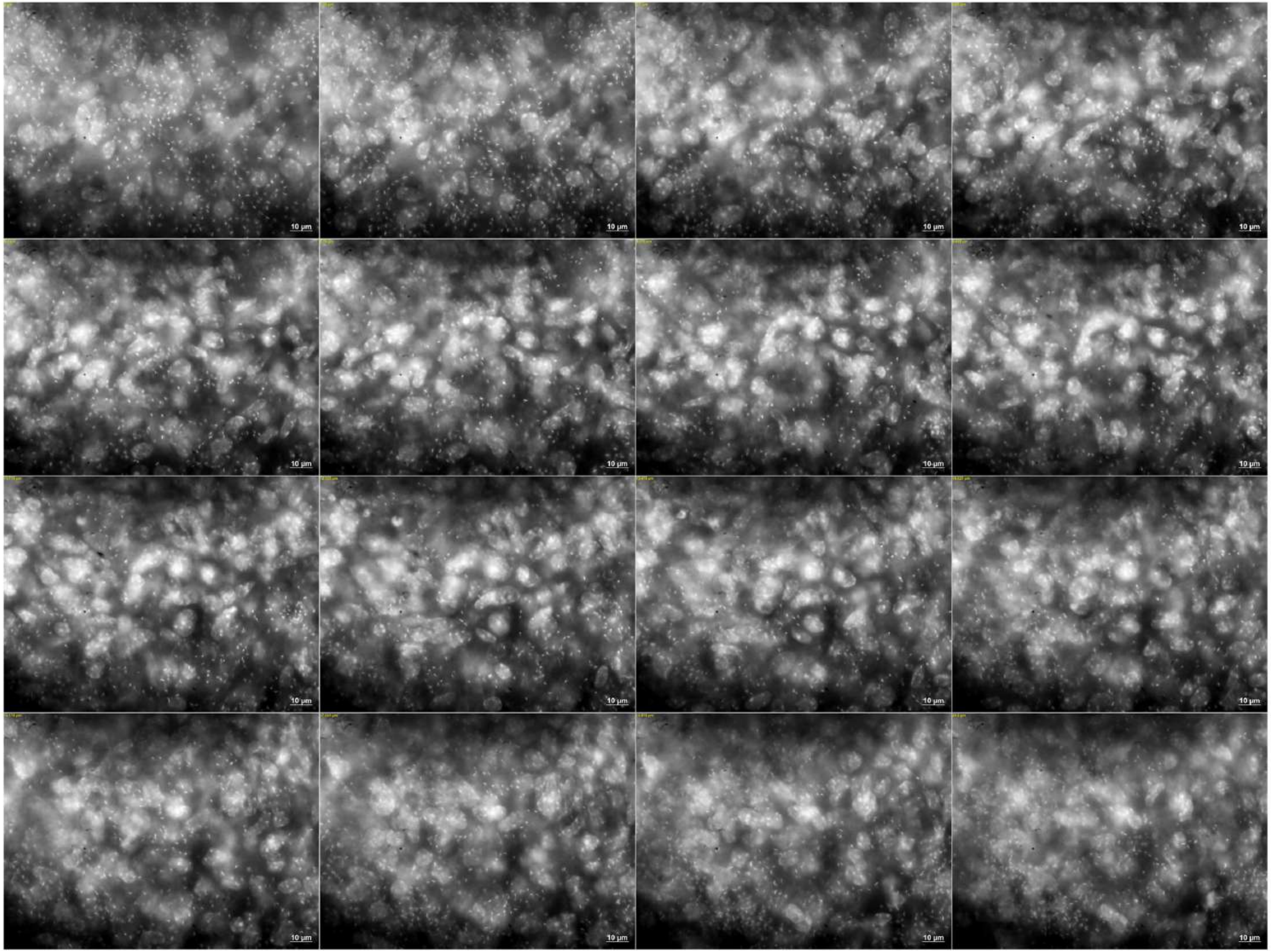

Figure S1. Z-axis images of *T. cruzi*-infected 3D cardiac spheroids stained with DNA dye. Serial Z-axis fluorescence images (22 µm interval) revealed the presence of intracellular parasites throughout the spheroid (outer and inner layers). DAPI stained the host cell nucleus and the nuclear and kinetoplast DNA of the parasite. Bar = 10 µm

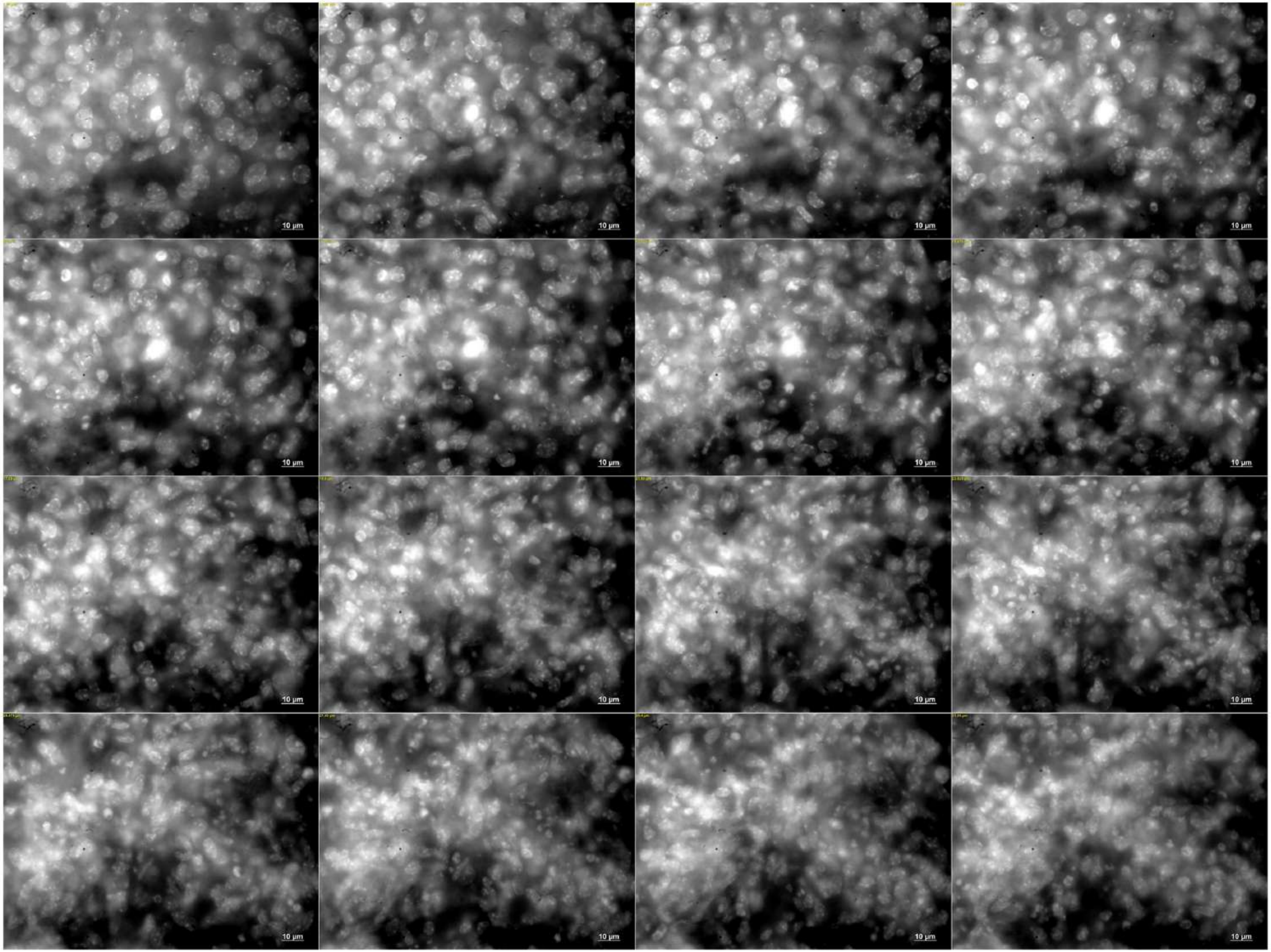

Figure S2. Sequence of optical sections in the Z axis of cardiac spheroids infected by *T. cruzi* after 72h treatment with **2k** analog (84.54 µM). A drastic reduction of intracellular amastigotes was noticed, with very few parasites visualized in the inner layers of the spheroid. Host cell nucleus and the nuclear and kinetoplast DNA of the parasites were visualized by DAPI satining. Bar = 10 µm

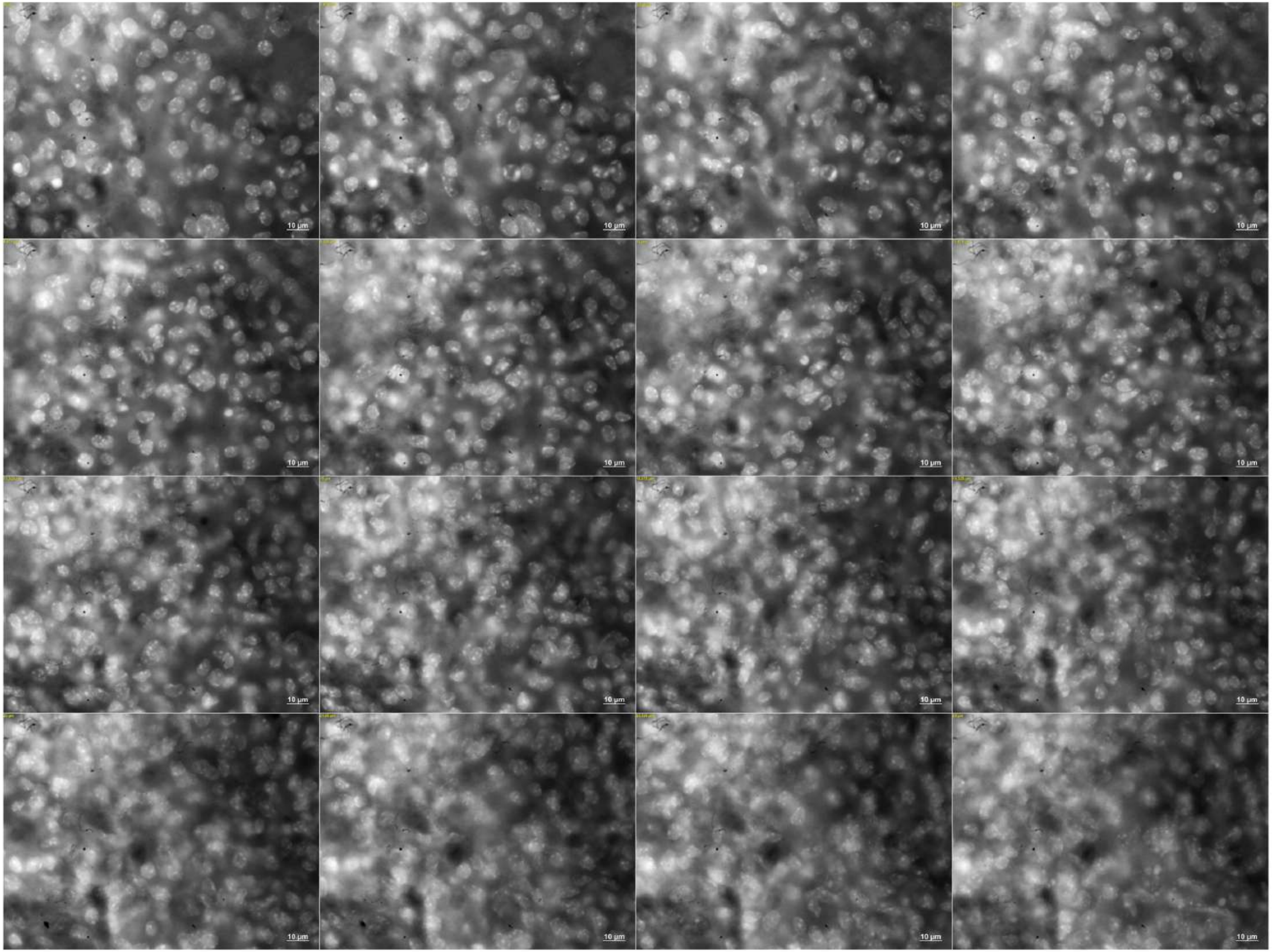

Figure S3. Fluorescence images of Z-axis sections of cardiac spheroids infected by *T. cruzi* and treated for 72 h with Bz (100 µM). DAPI staining (DNA dye) reveled rare parasites throughout the 3D cardiac spheroid. Bar = 10 µm

^{1}H NMR of compound **1a**

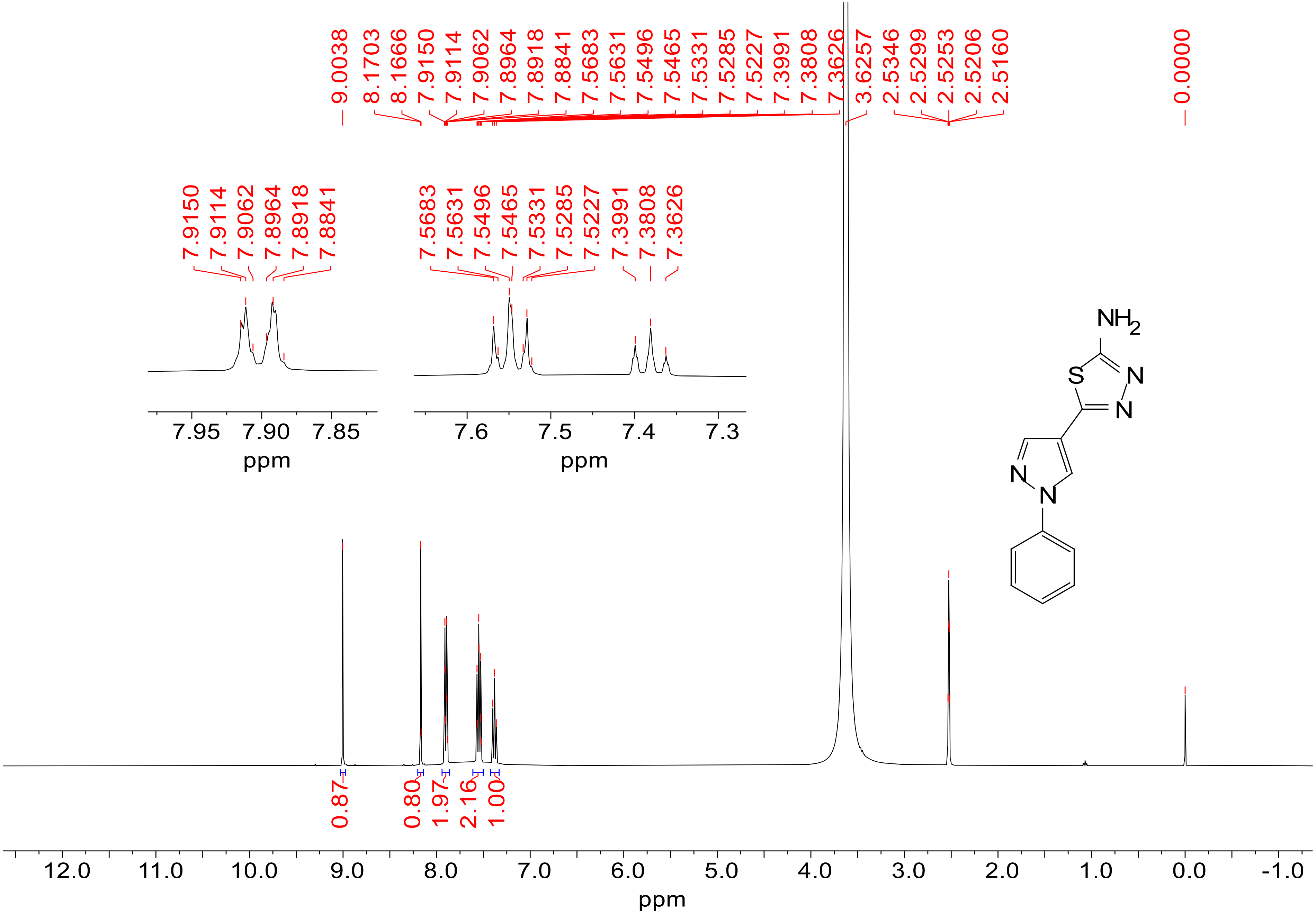

^{13}C NMR of compound **1a**

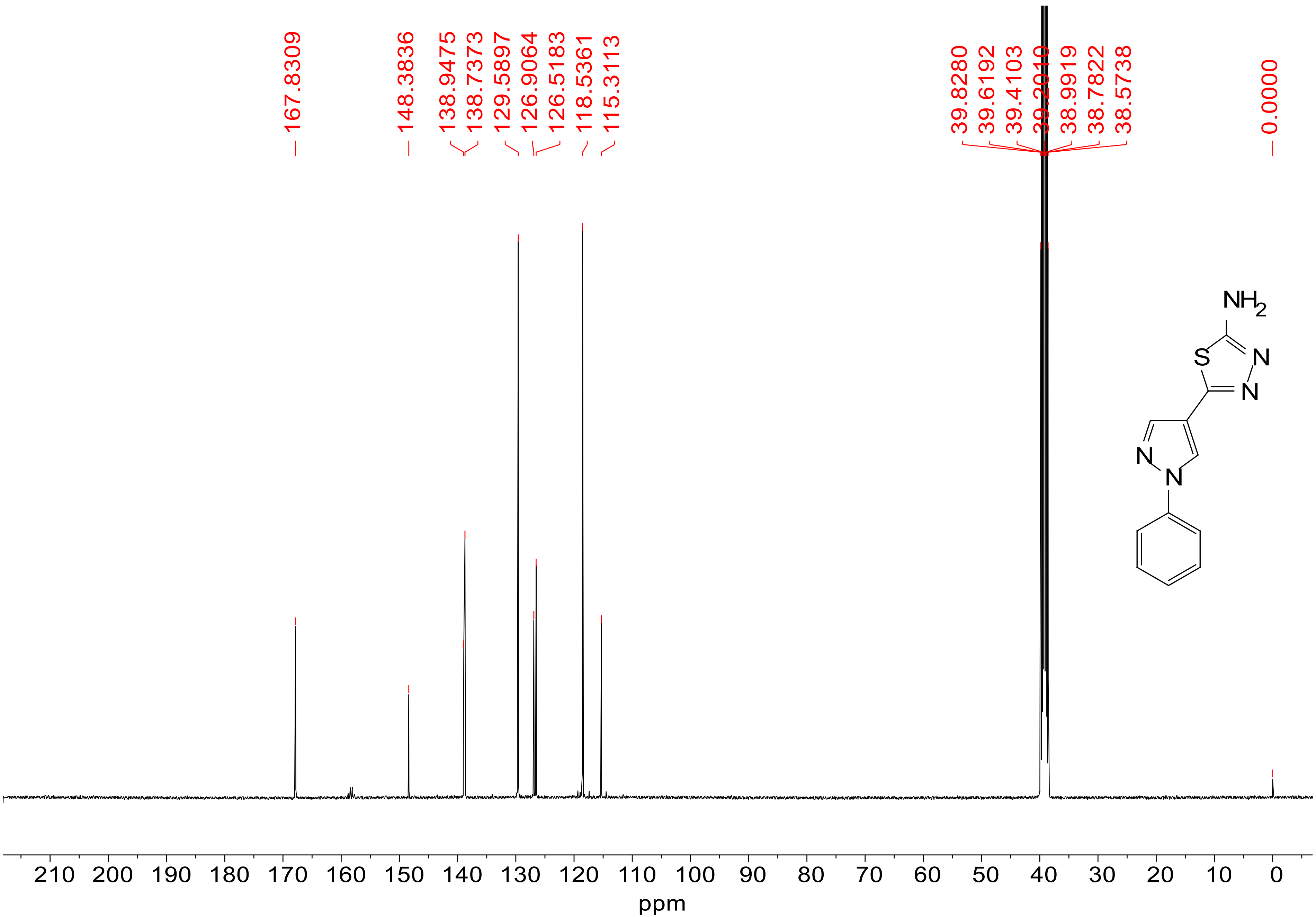

^{1}H NMR of compound **1b**

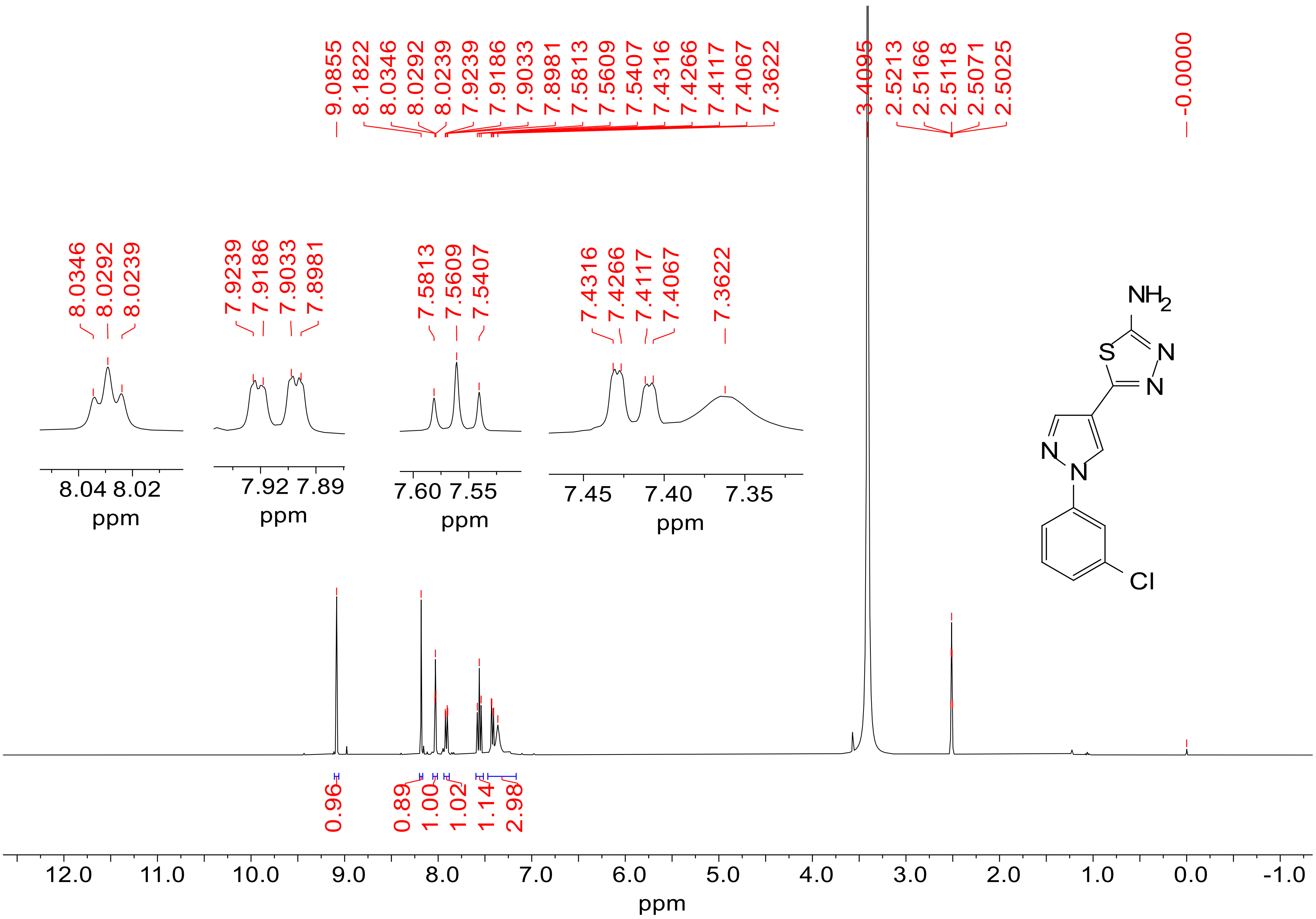

^{13}C NMR of compound **1b**

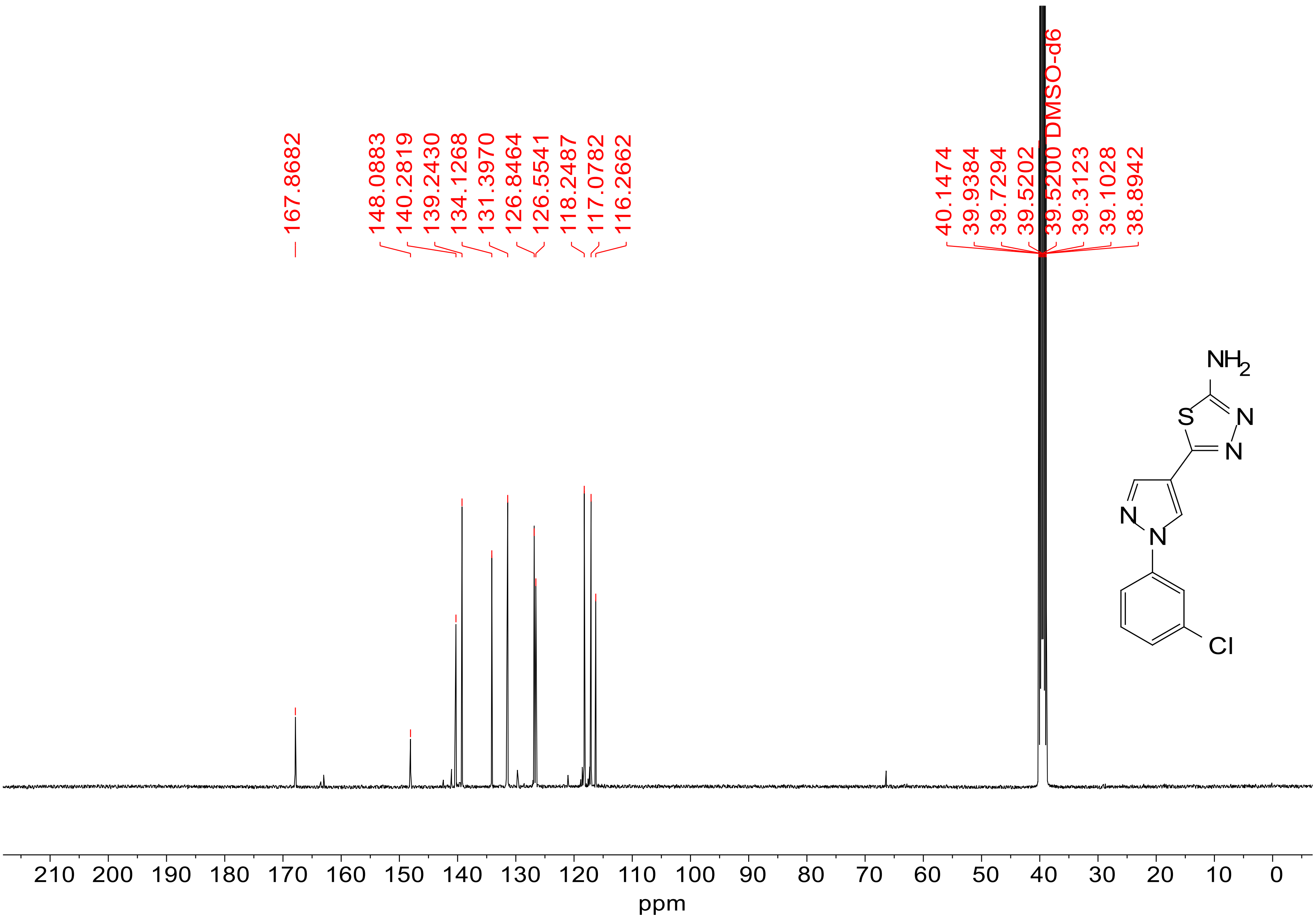

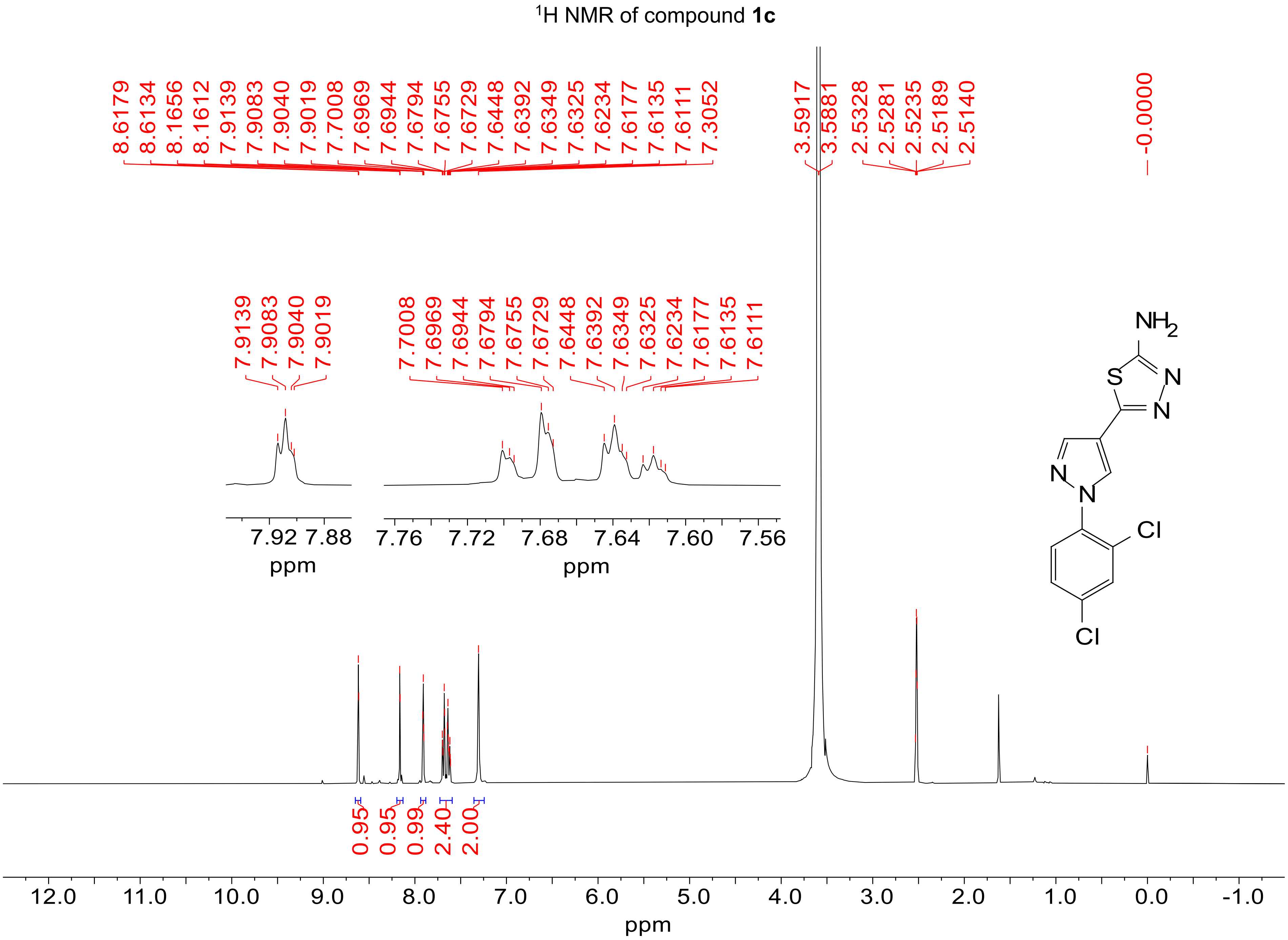

1H NMR of compound 1c
8.6179 8.6134 8.1656 8.1612 7.9139 7.9083 7.9040 7.9019 7.7008 7.6969 7.6944 7.6794 7.6755 7.6729 7.6448 7.6392 7.6349 7.6325 7.6234 7.6177 7.6135 7.6111 7.3052
3.5917 3.5881 2.5328 2.5281 2.5235 2.5189 2.5140
-0.0000
0.95 0.95 0.99 2.40 2.00
ppm
NH2
S
N
N
N
N
Cl
Cl

^{13}C NMR of compound **1c**

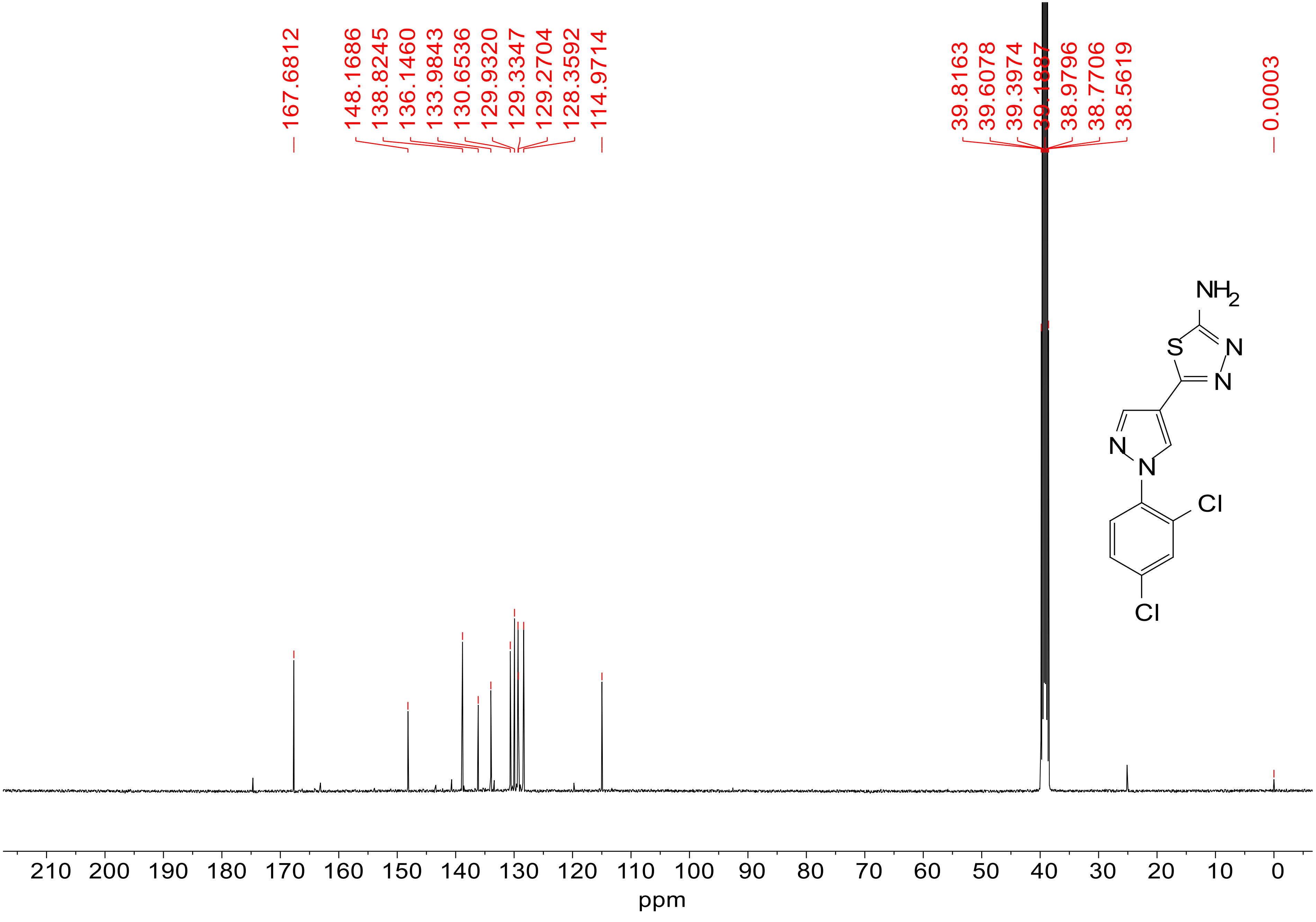

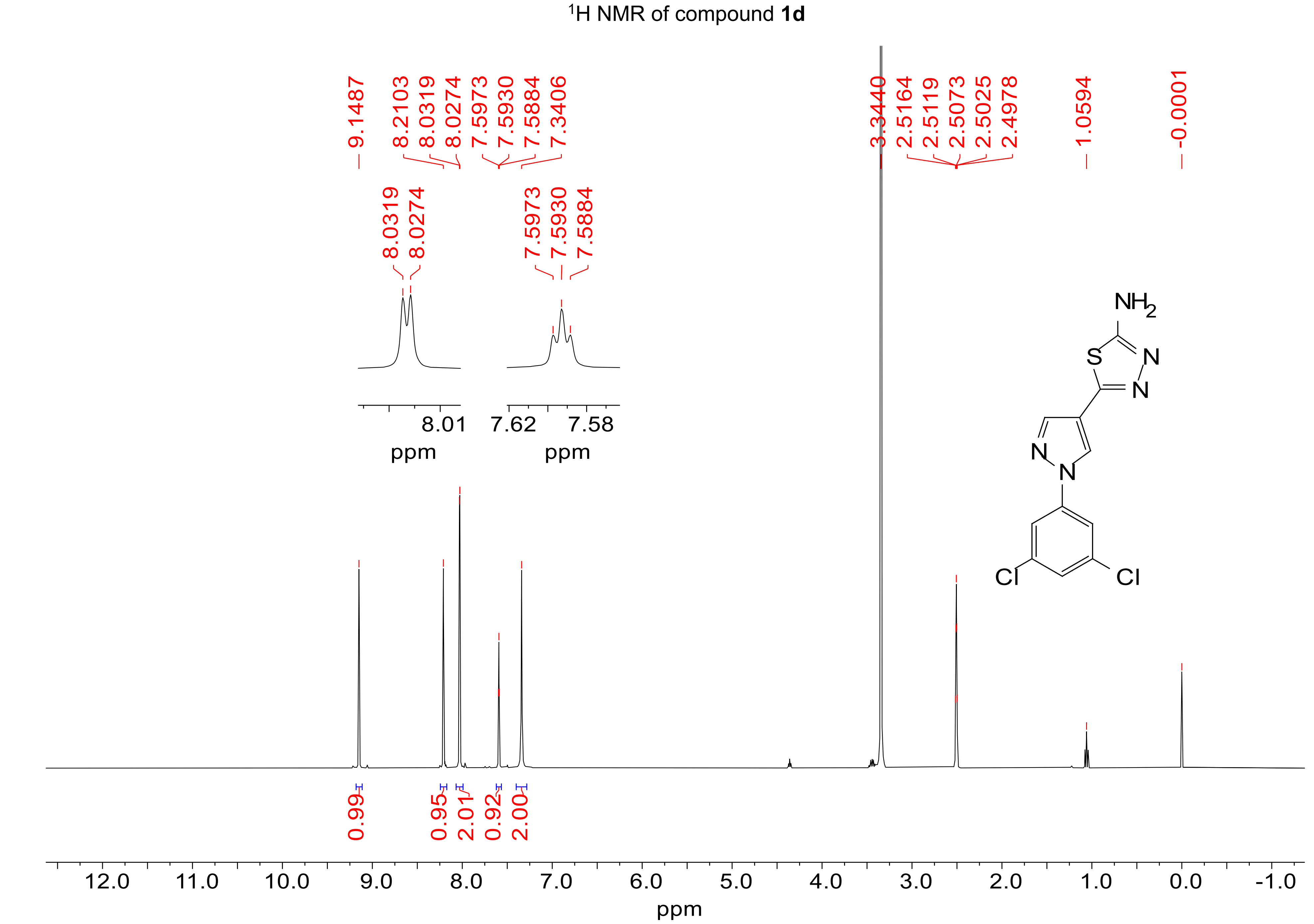
^{1}H NMR of compound **1d**
9.1487
8.2103
8.0319
8.0274
7.5973
7.5930
7.5884
7.3406
3.3440
2.5164
2.5119
2.5073
2.5025
2.4978
1.0594
-0.0001
8.0319
8.0274
7.5973
7.5930
7.5884
8.01
ppm
7.62
7.58
ppm
NH2
S
N
N
N
N
Cl
Cl
0.99
0.95
2.01
0.92
2.00
12.0
11.0
10.0
9.0
8.0
7.0
6.0
5.0
4.0
3.0
2.0
1.0
0.0
-1.0
ppm

^{13}C NMR of compound **1d**

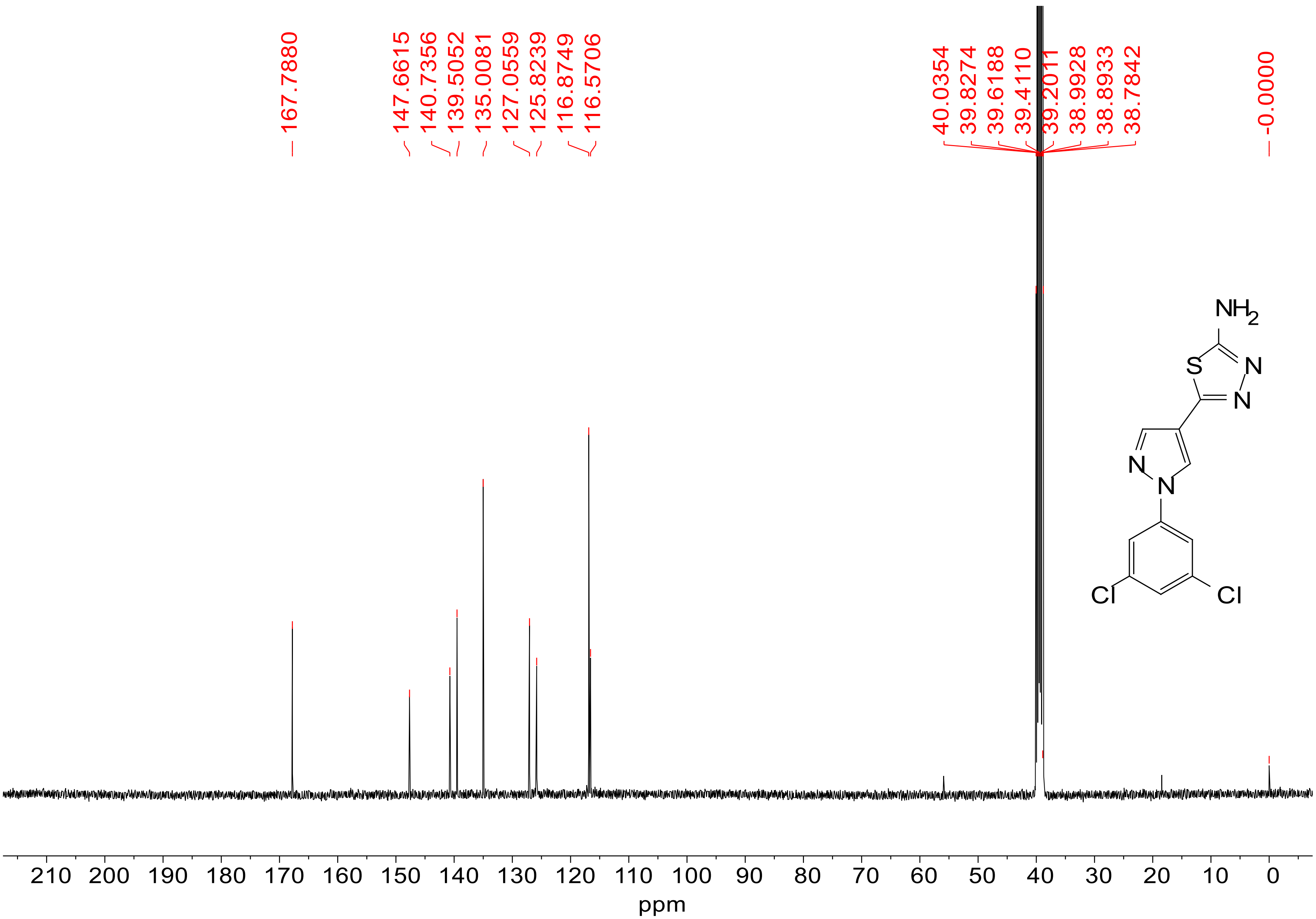

^{1}H NMR of compound **1e**

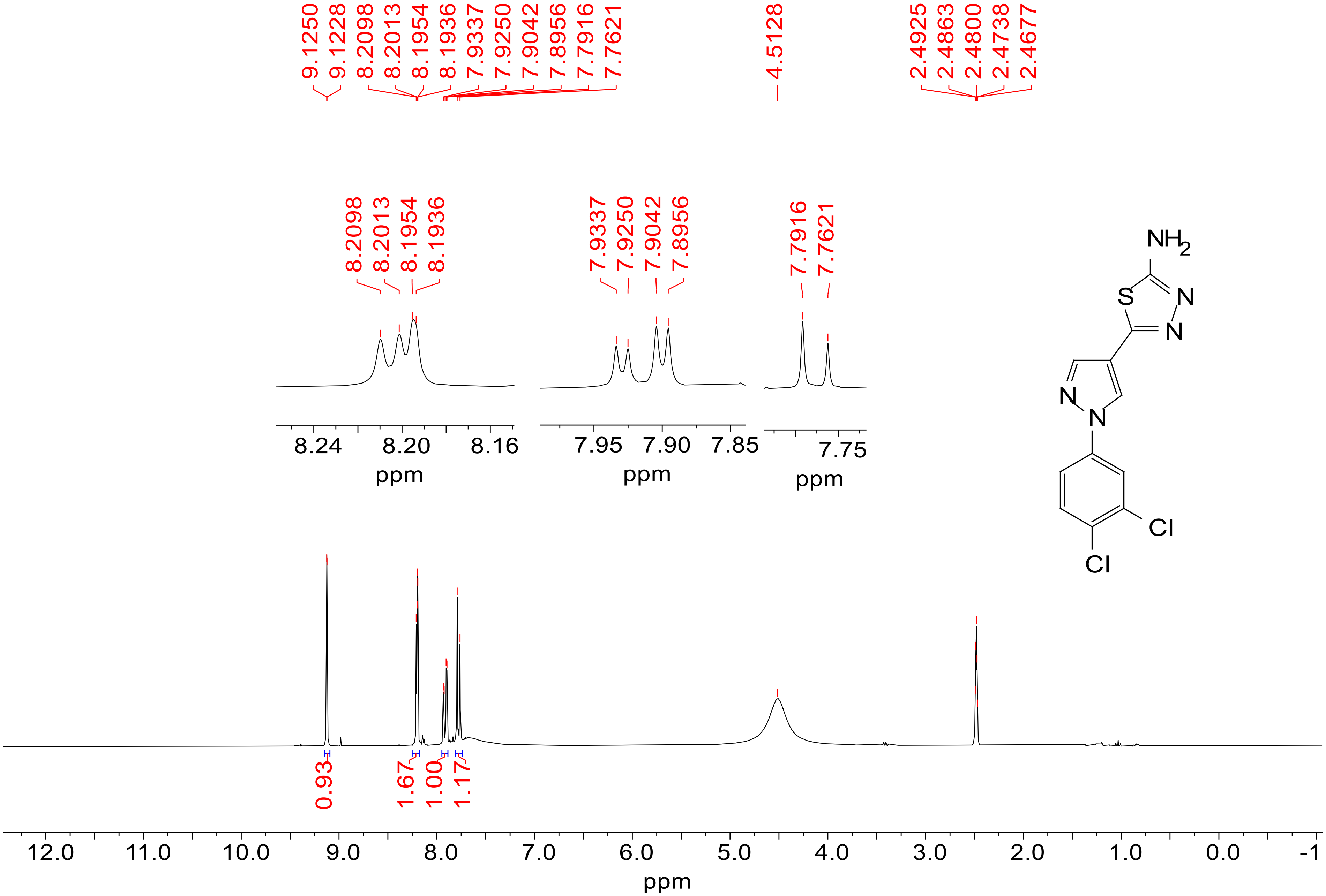

^{13}C NMR of compound **1e**

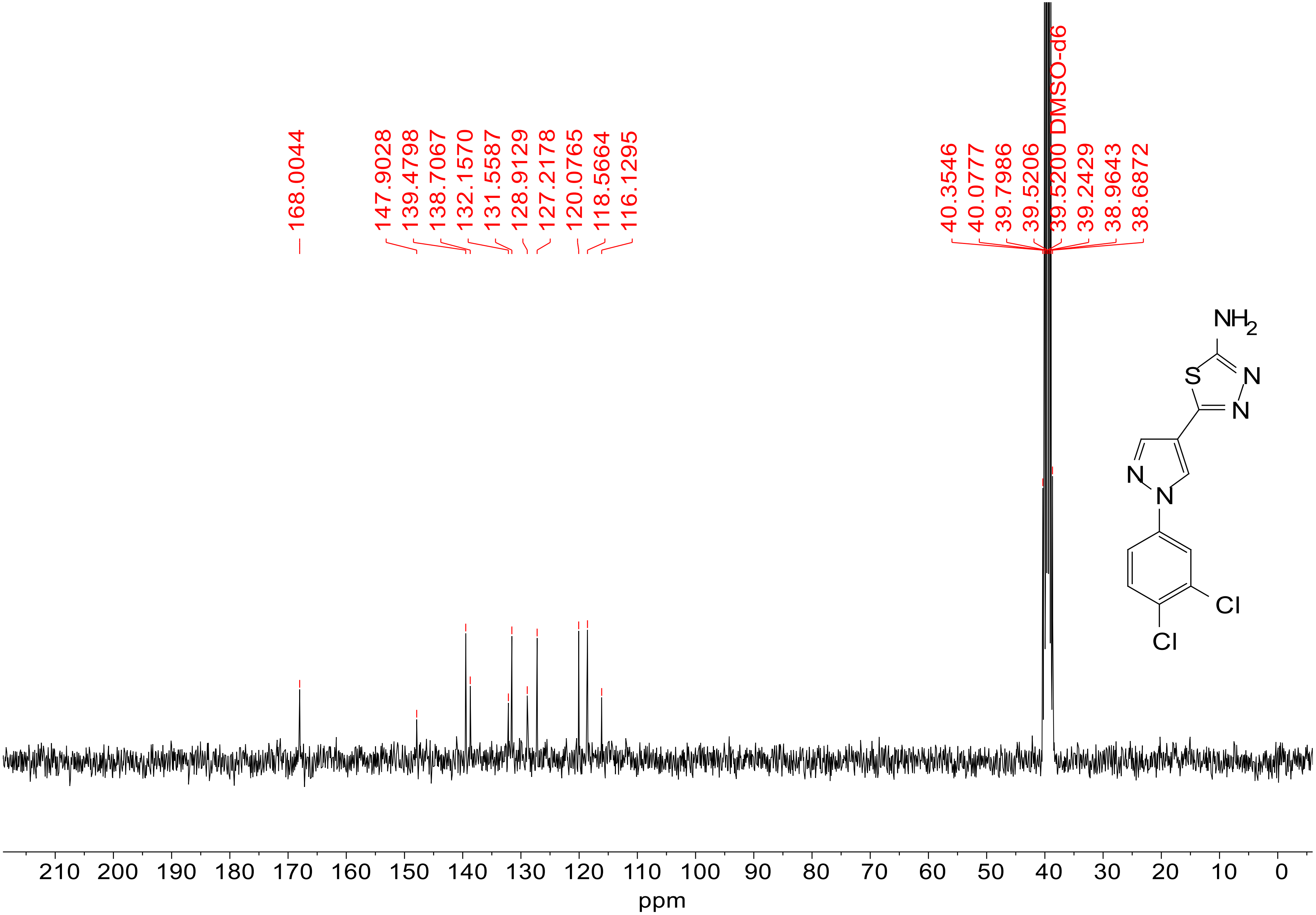

^{1}H NMR of compound **1f**

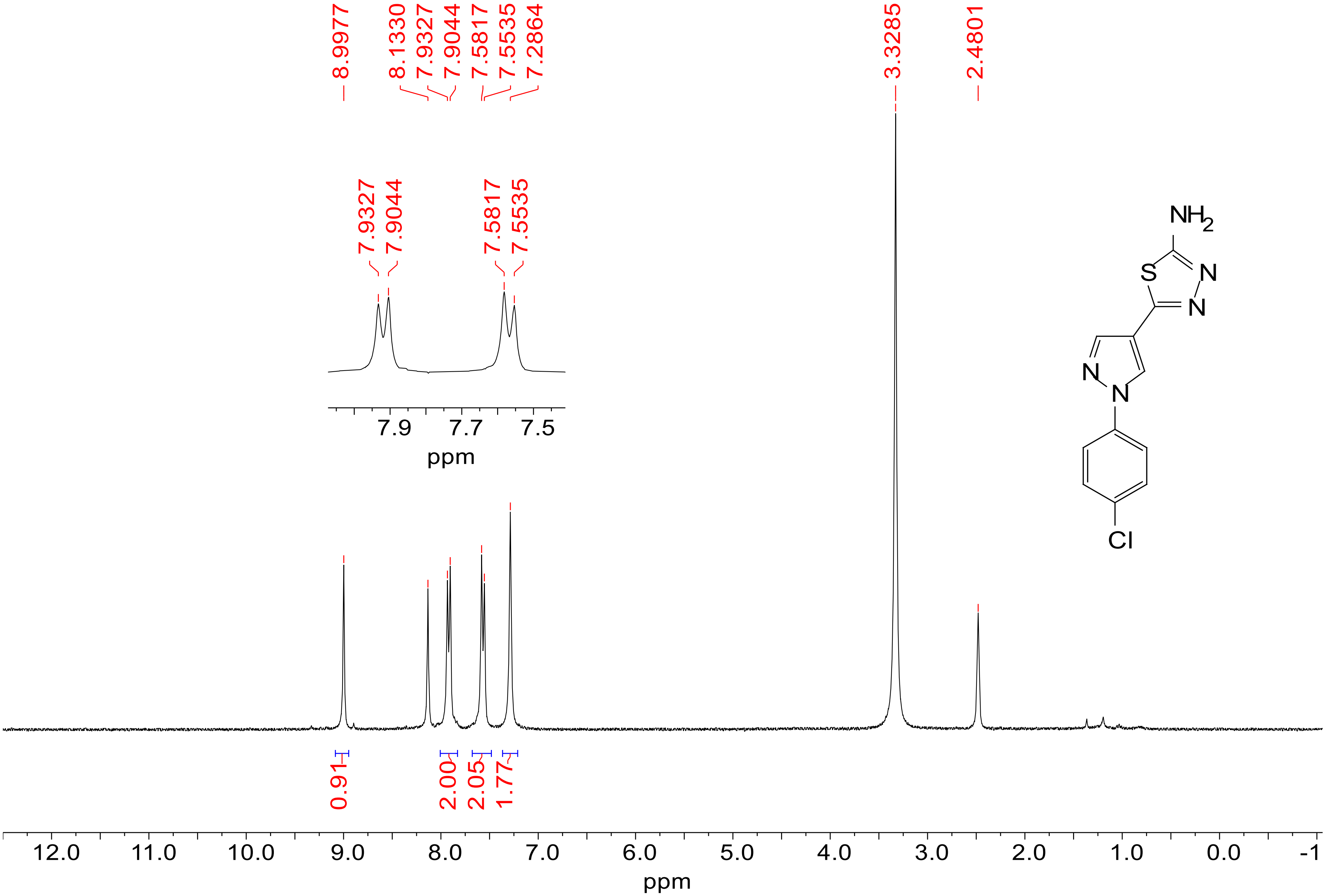

^{13}C NMR of compound **1f**

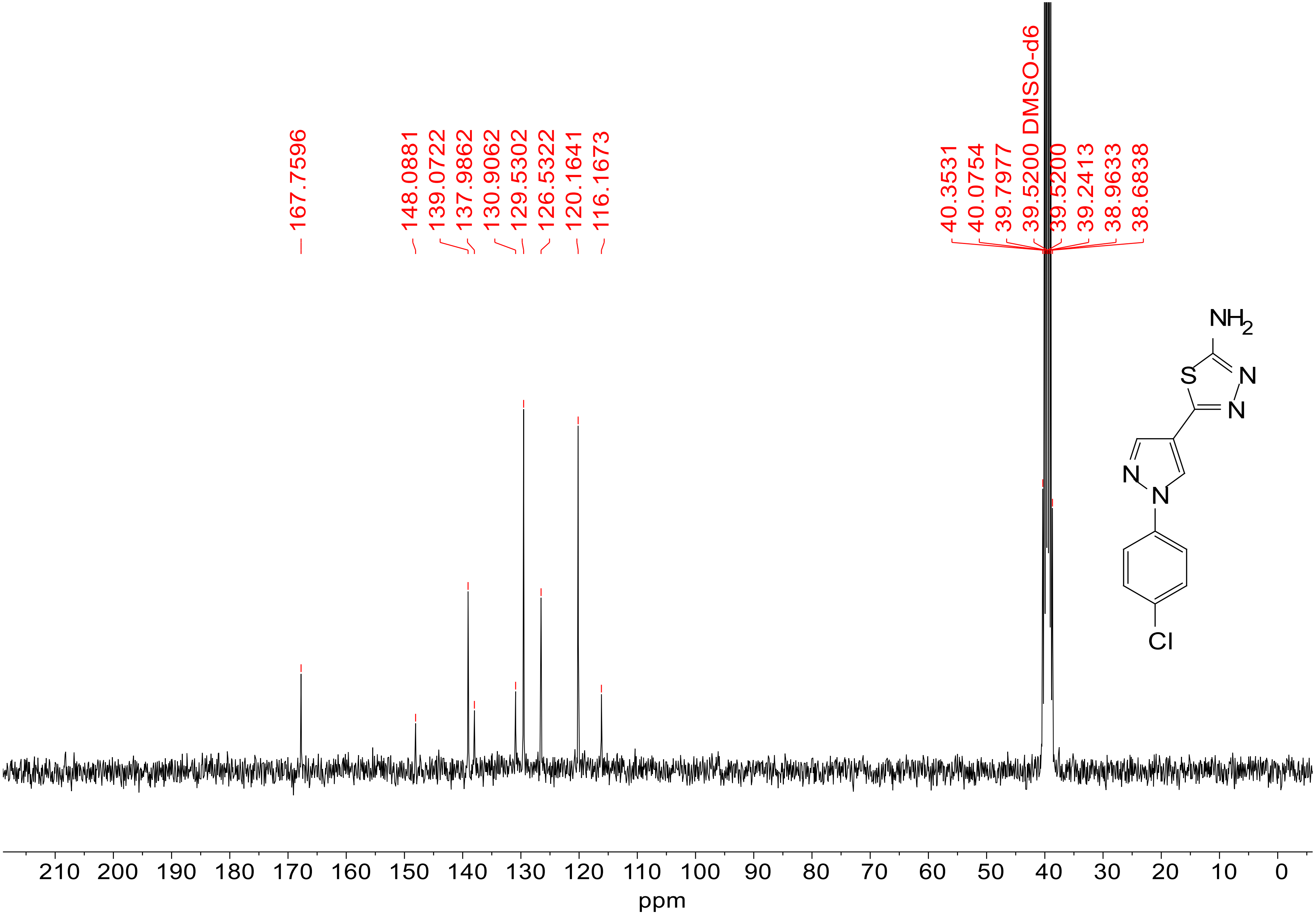

^{1}H NMR of compound **1g**

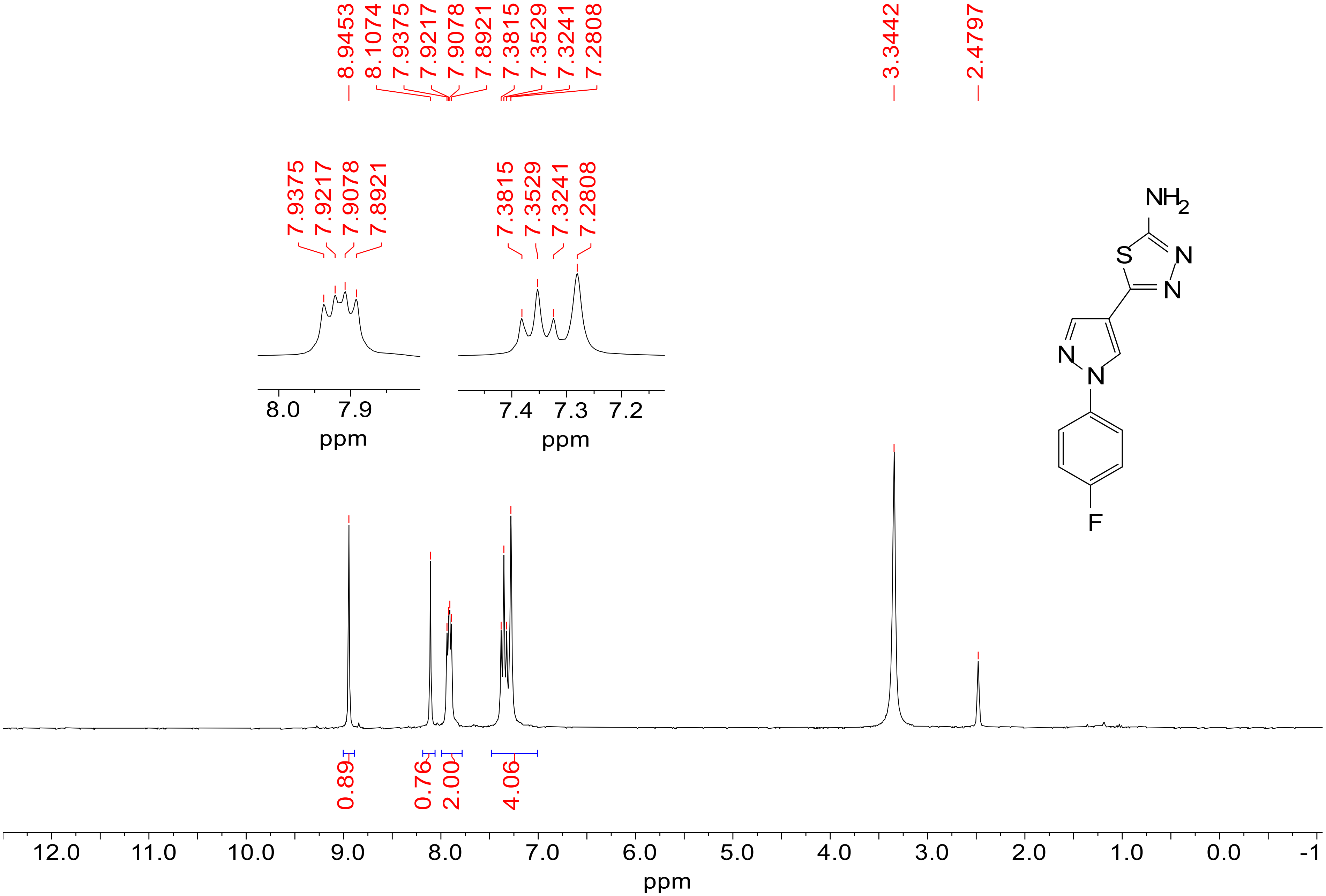

^{13}C NMR of compound **1g**

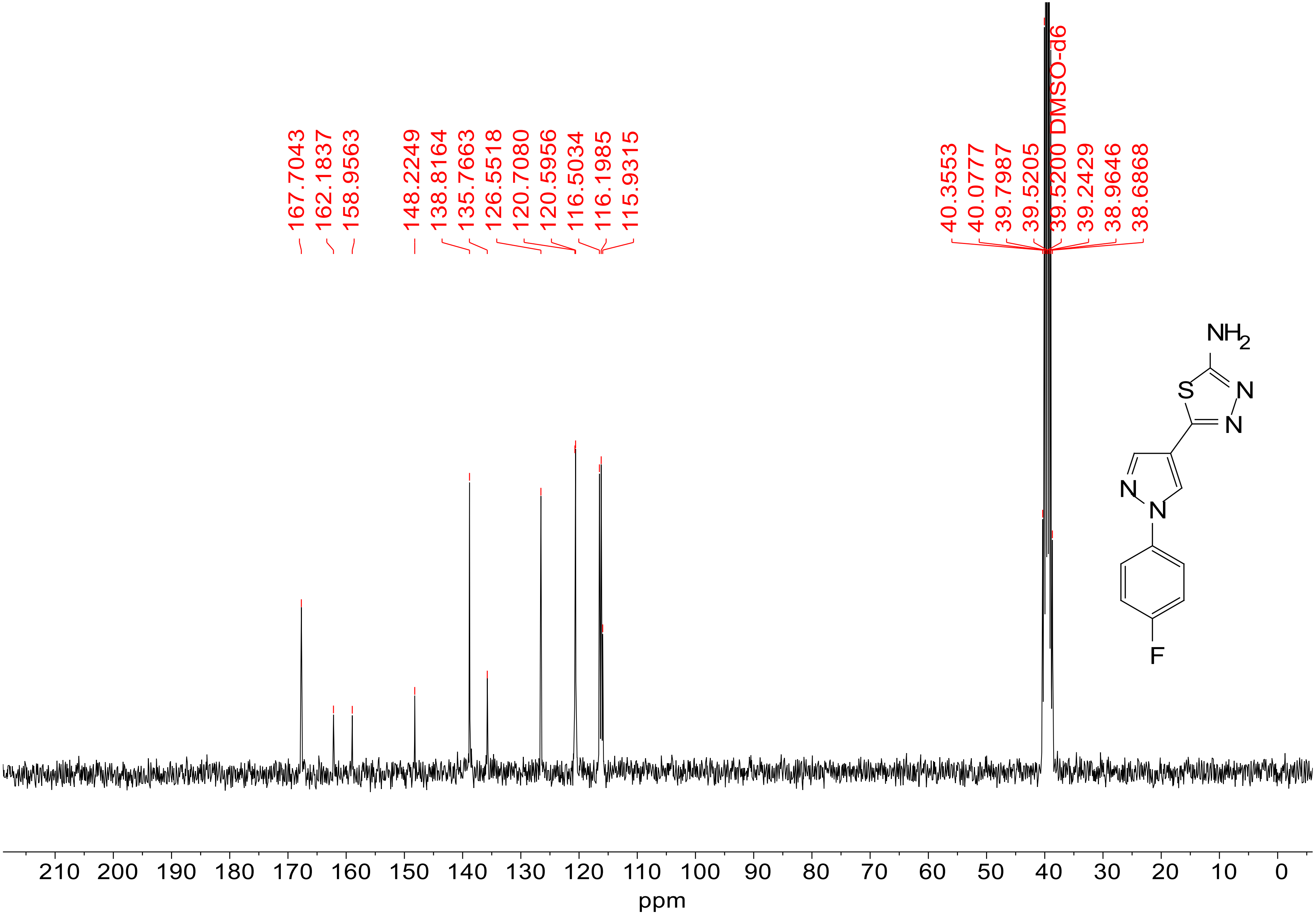

^{1}H NMR of compound **1h**

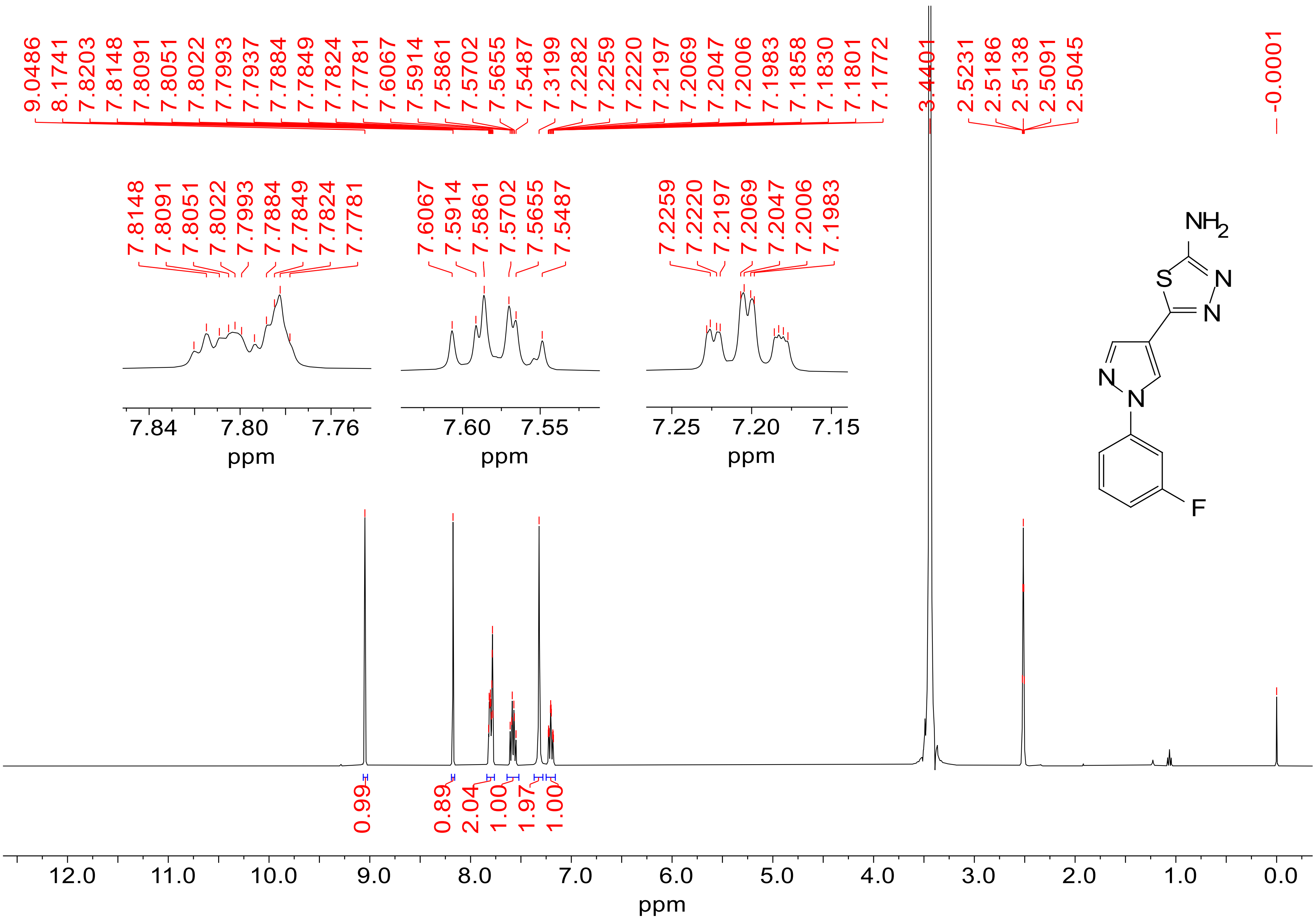

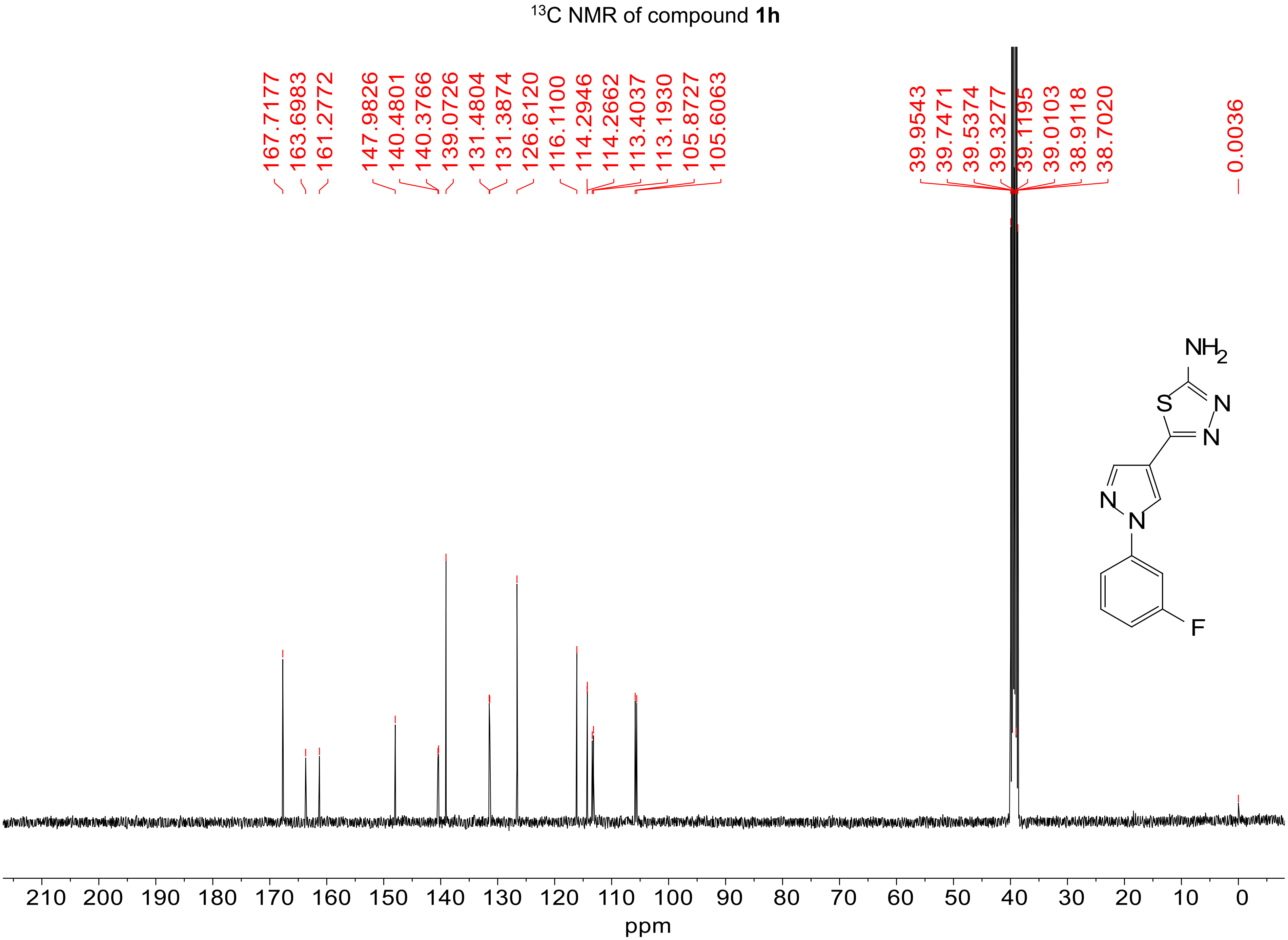

^{13}C NMR of compound **1h**
167.7177
163.6983
161.2772
147.9826
140.4801
140.3766
139.0726
131.4804
131.3874
126.6120
116.1100
114.2946
114.2662
113.4037
113.1930
105.8727
105.6063
39.9543
39.7471
39.5374
39.3277
39.1195
39.0103
38.9118
38.7020
0.0036
NH2
S
N
N
N
N
F
210 200 190 180 170 160 150 140 130 120 110 100 90 80 70 60 50 40 30 20 10 0
ppm

^{1}H NMR of compound **1i**

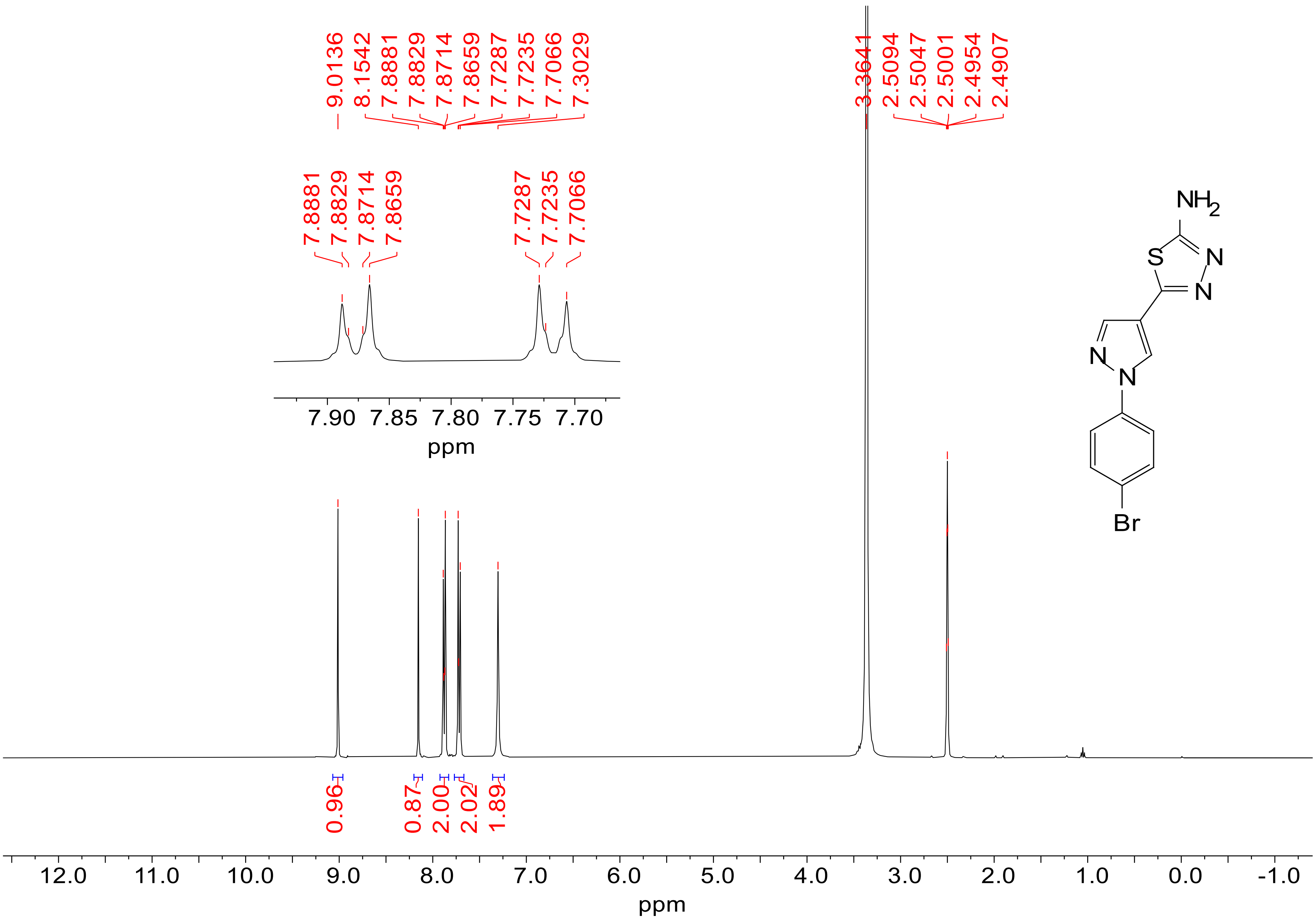

^{13}C NMR of compound **1i**

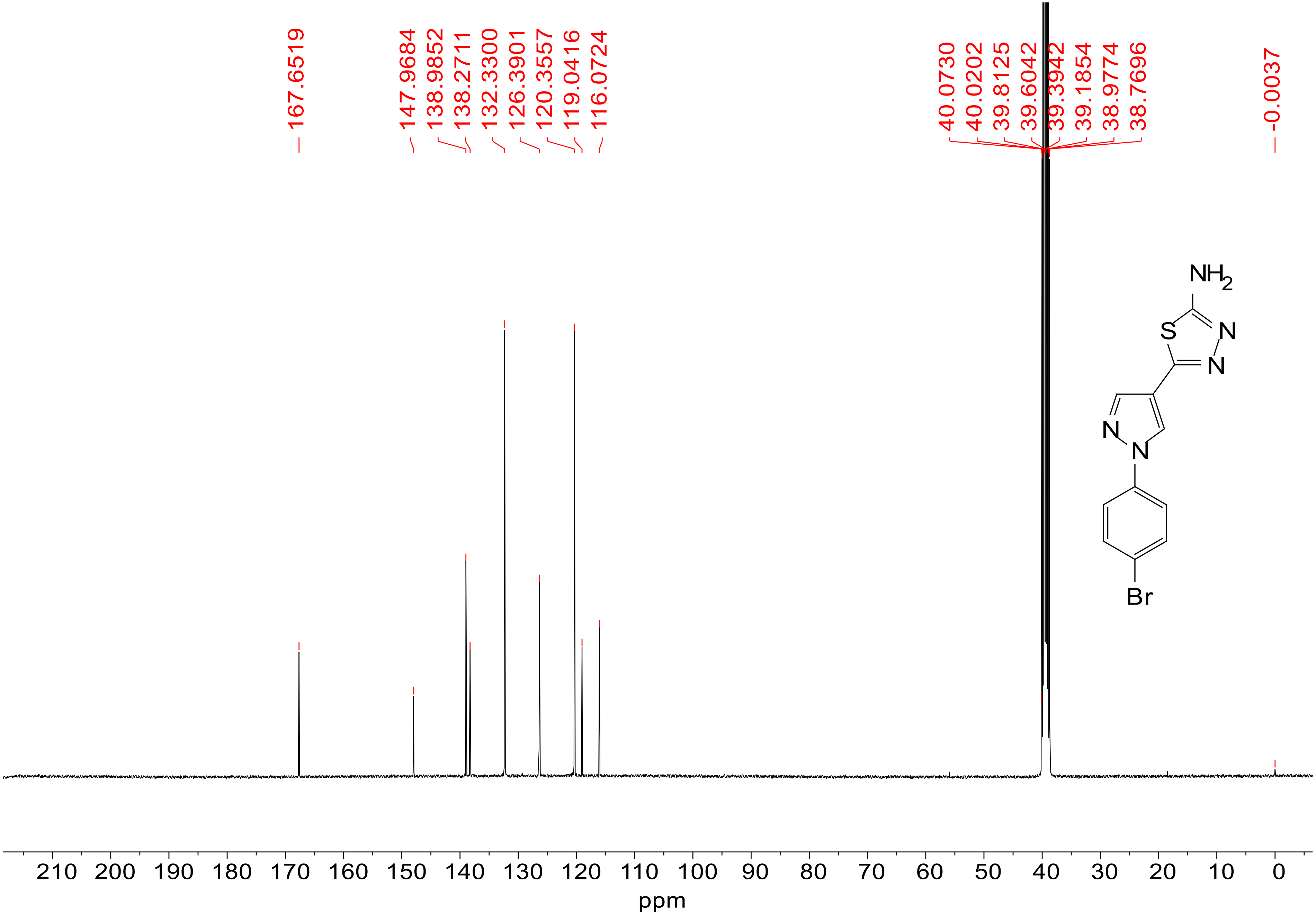

^{1}H NMR of compound **1j**

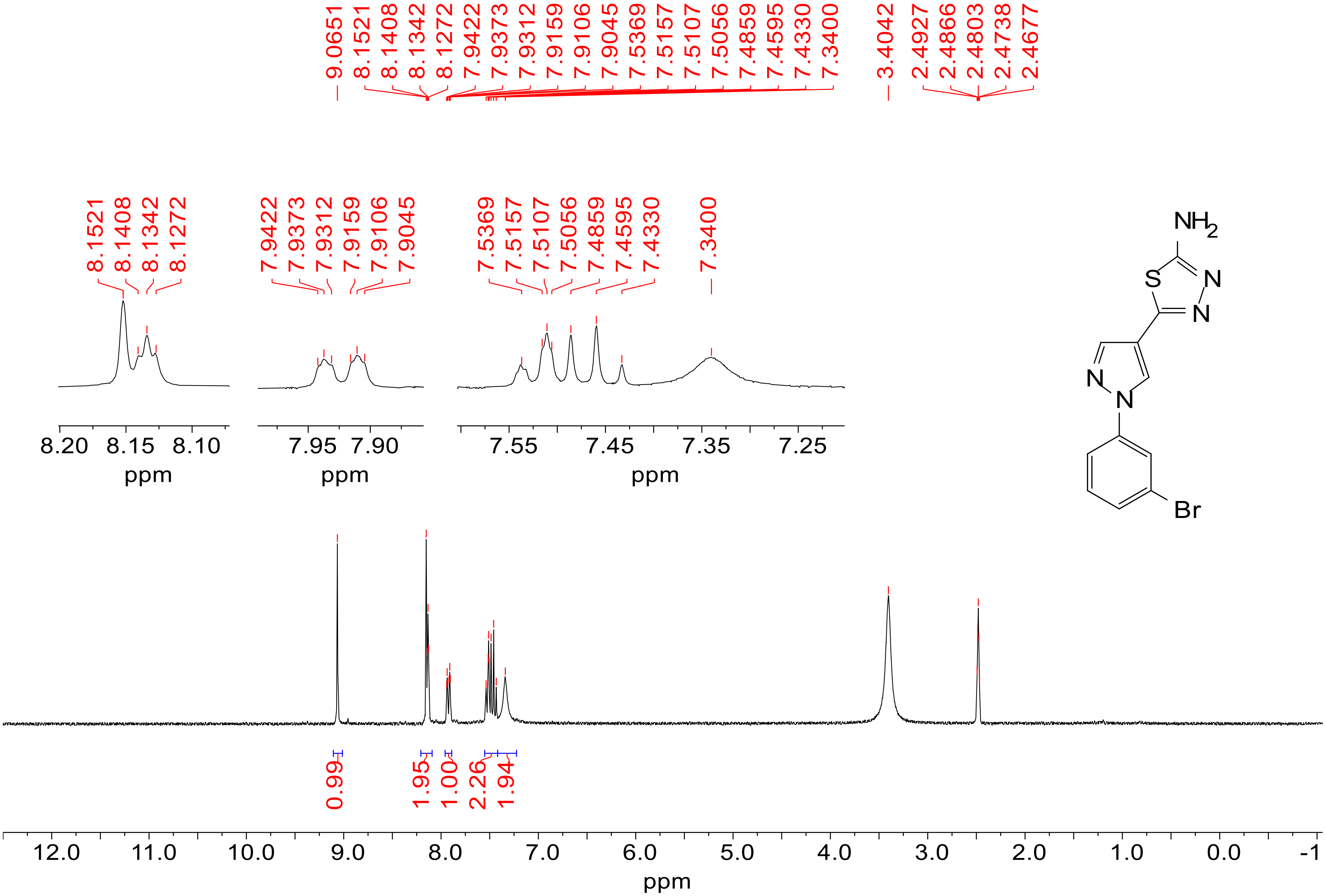

^{13}C NMR of compound **1j**

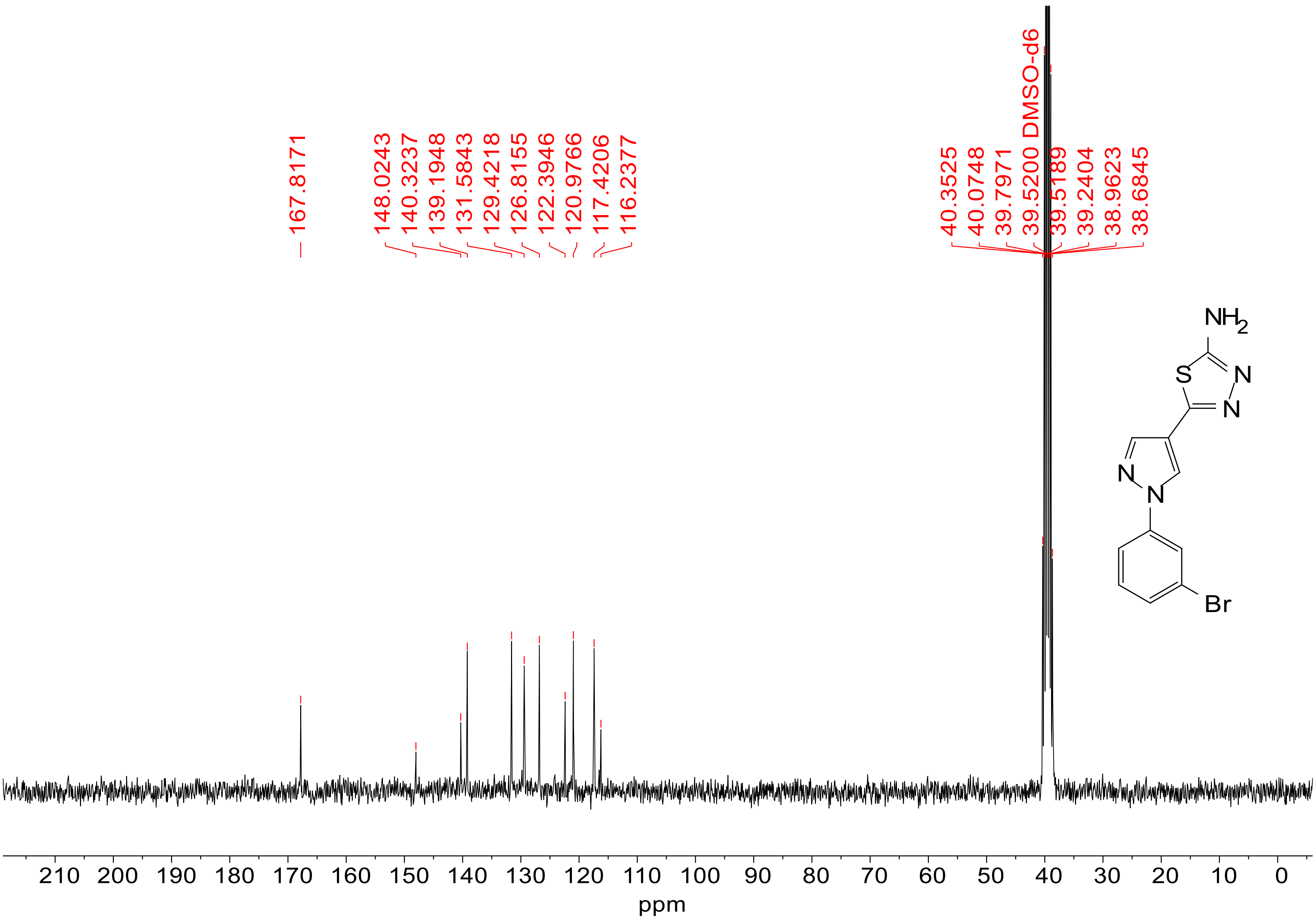

^{1}H NMR of compound **1k**

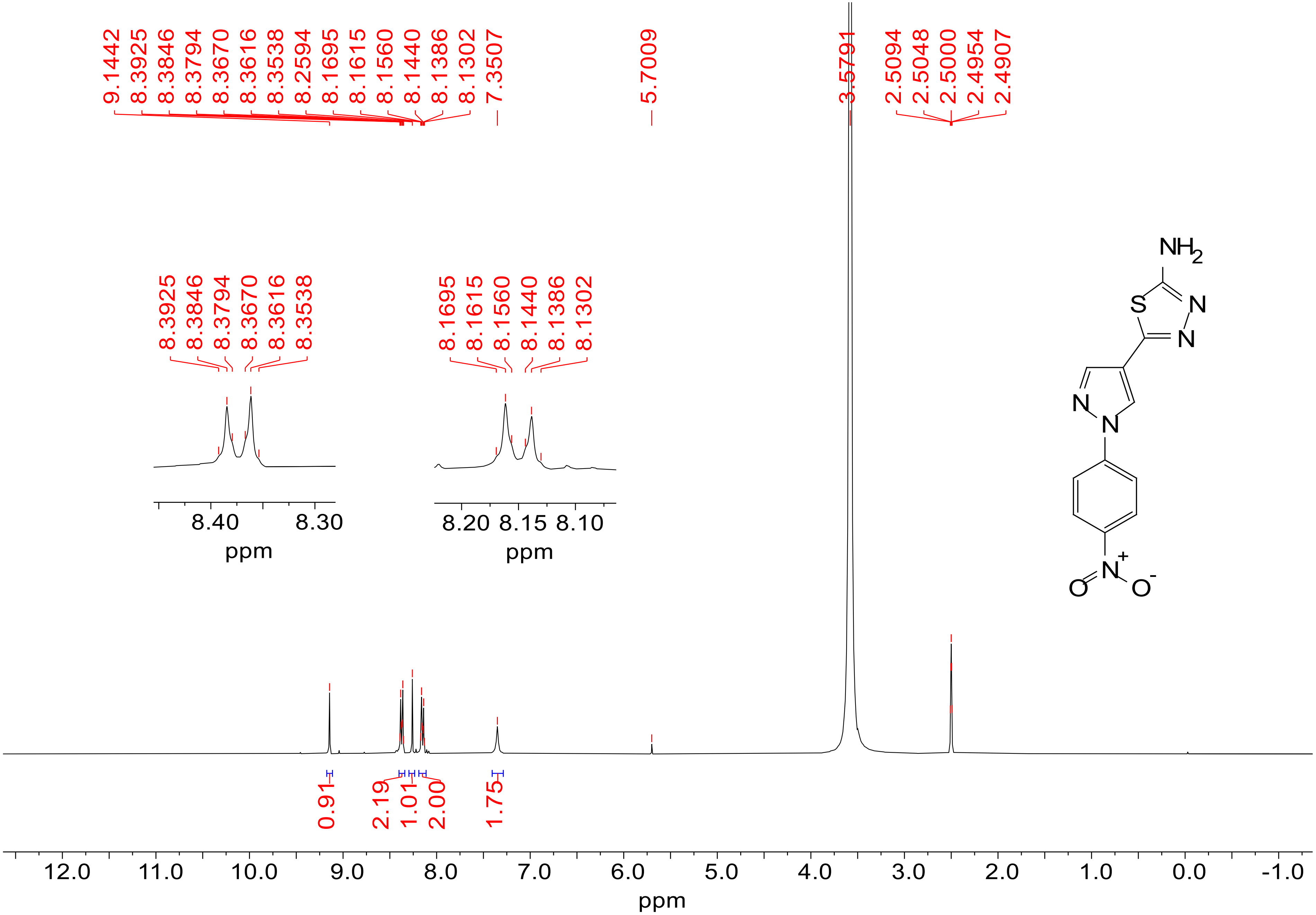

^{13}C NMR of compound **1k**

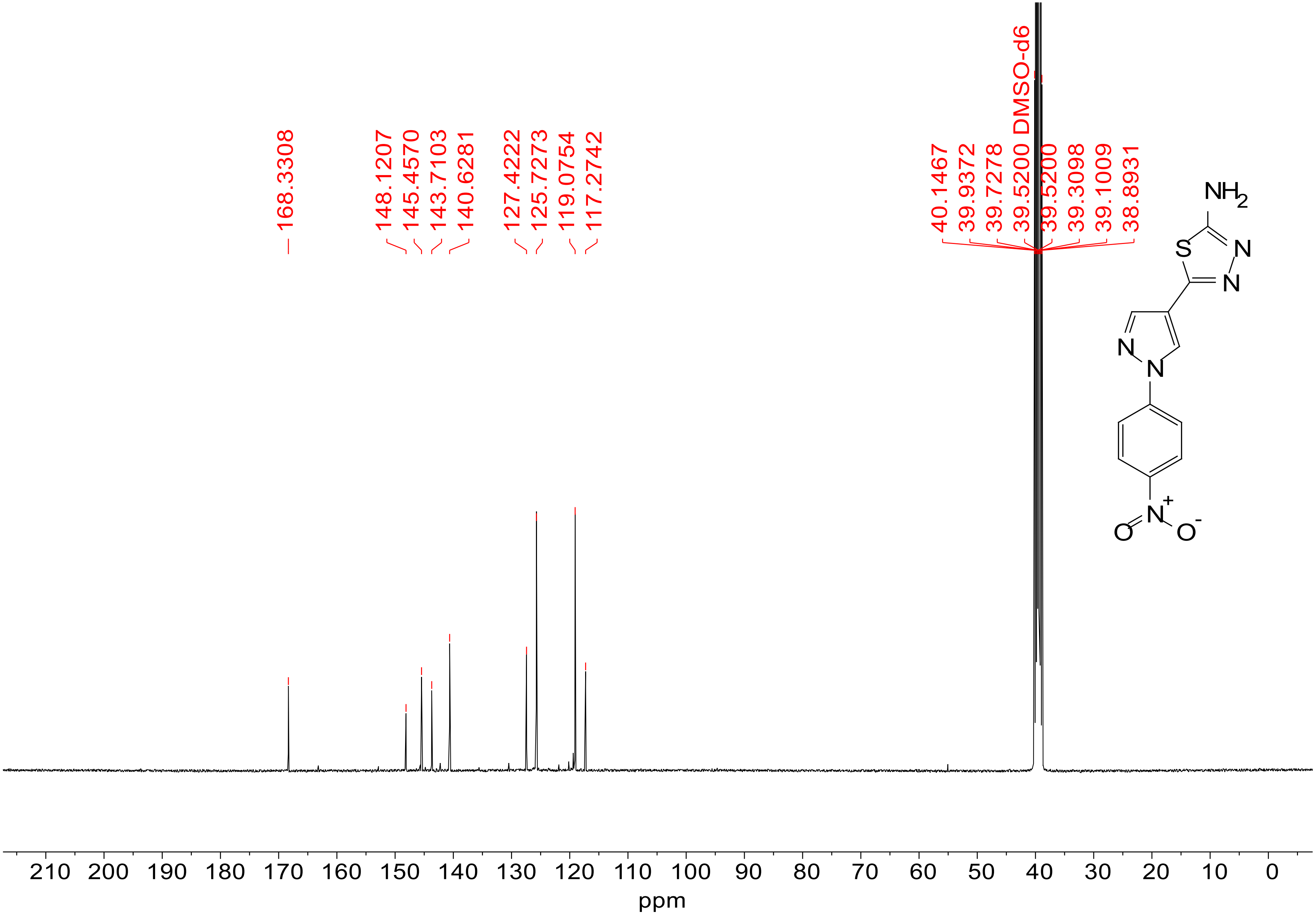

^{1}H NMR of compound **1l**

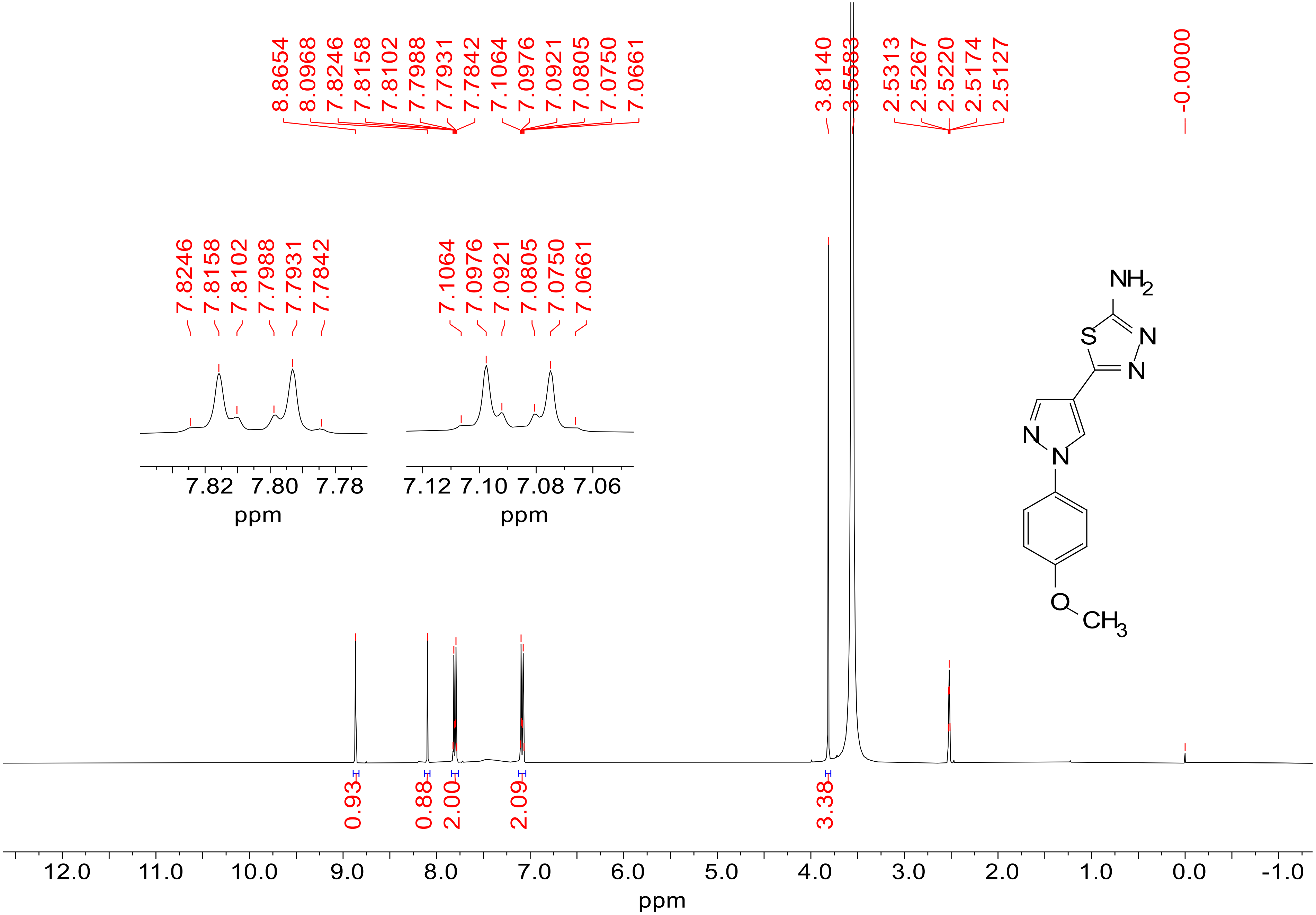

^{13}C NMR of compound **1l**

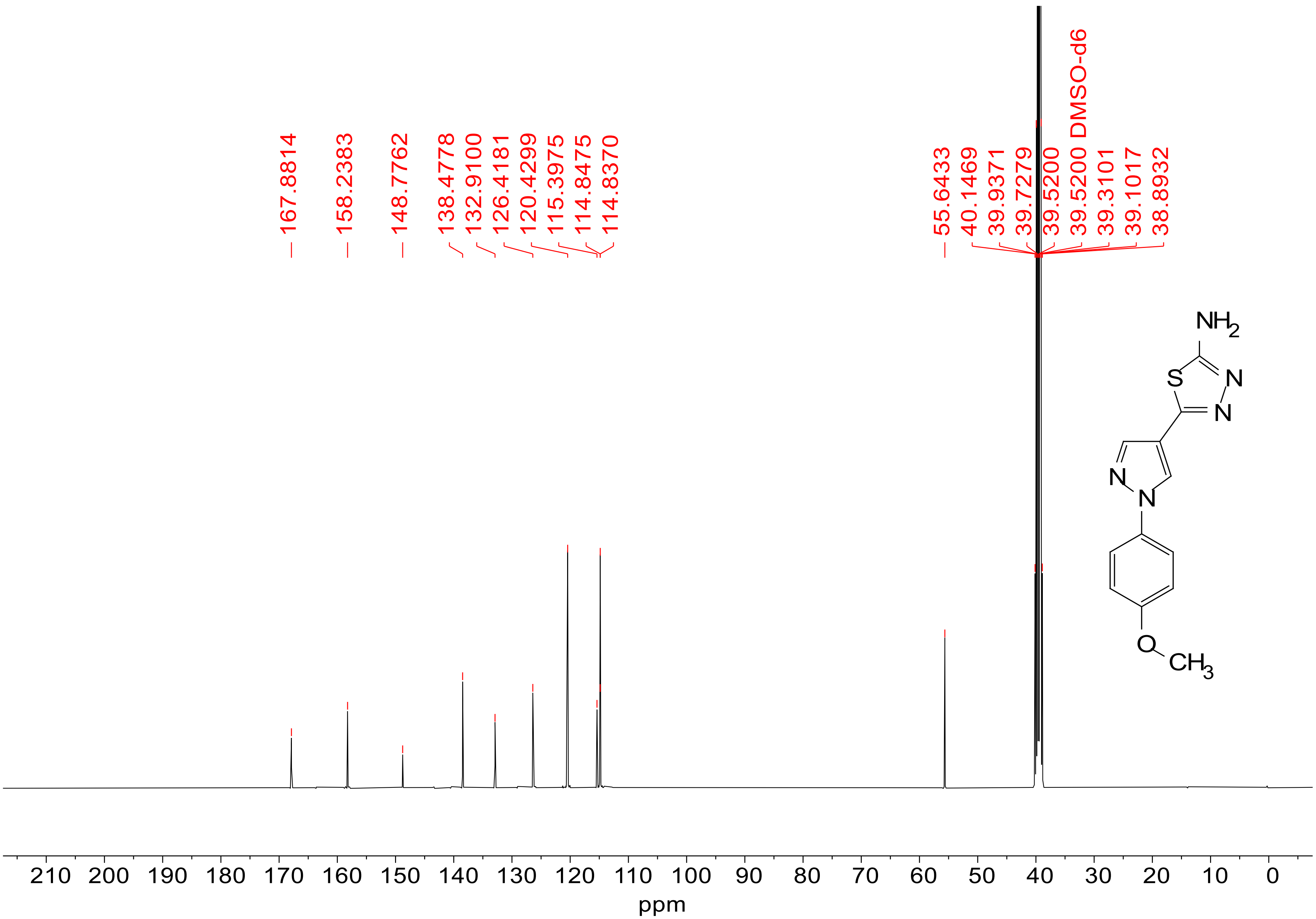

^{1}H NMR of compound **2a**

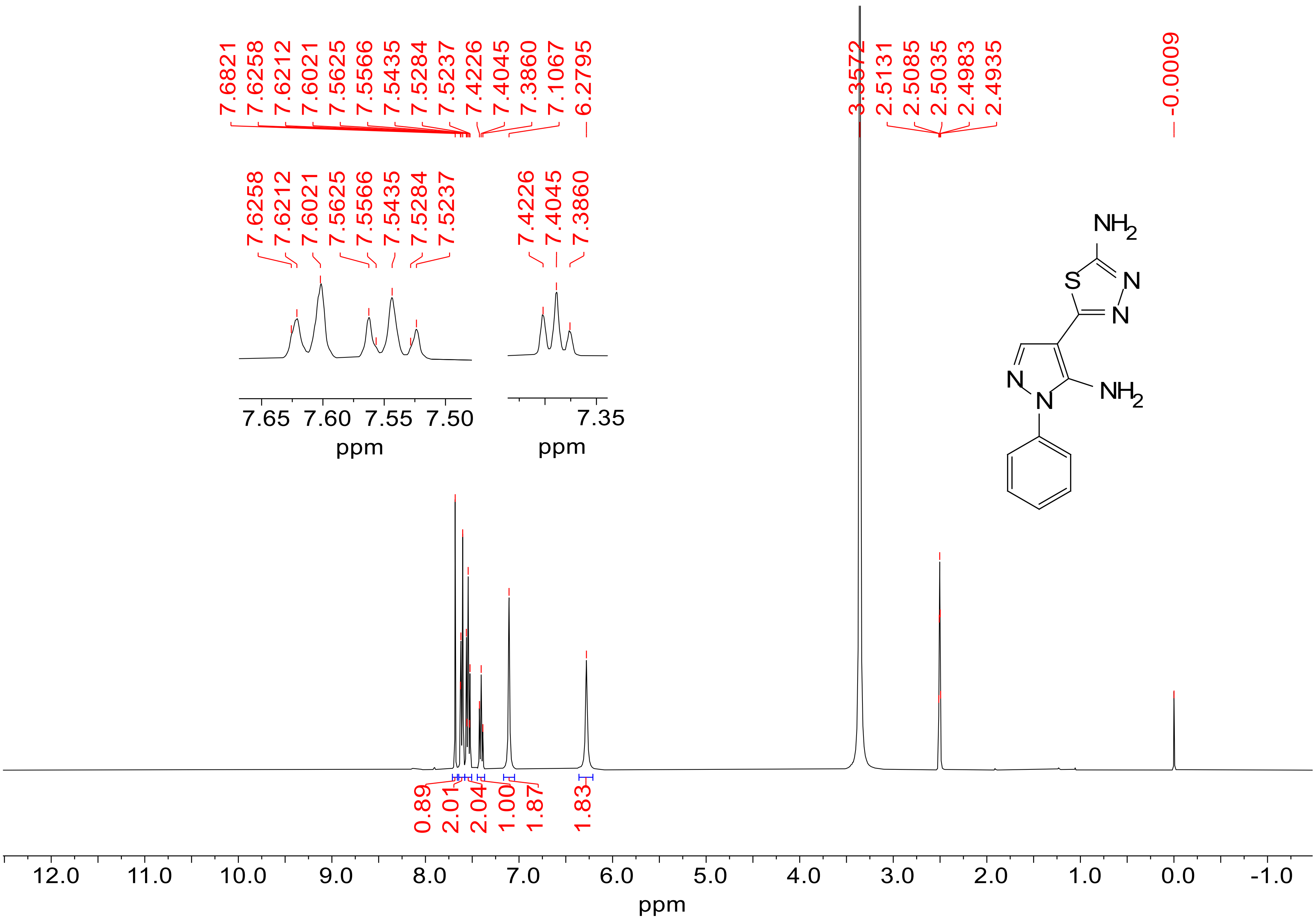

^{13}C NMR of compound **2a**

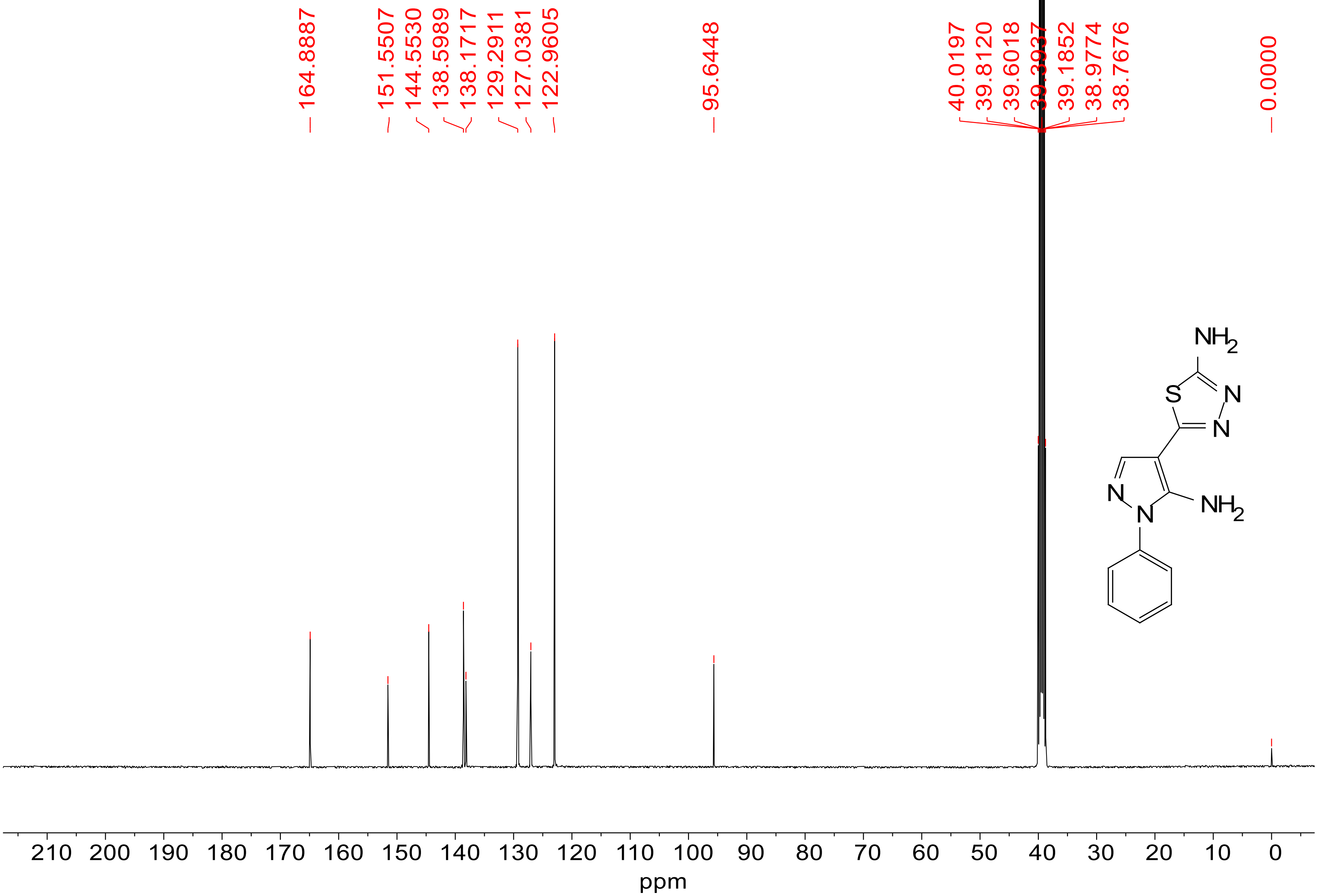

^{1}H NMR of compound **2b**

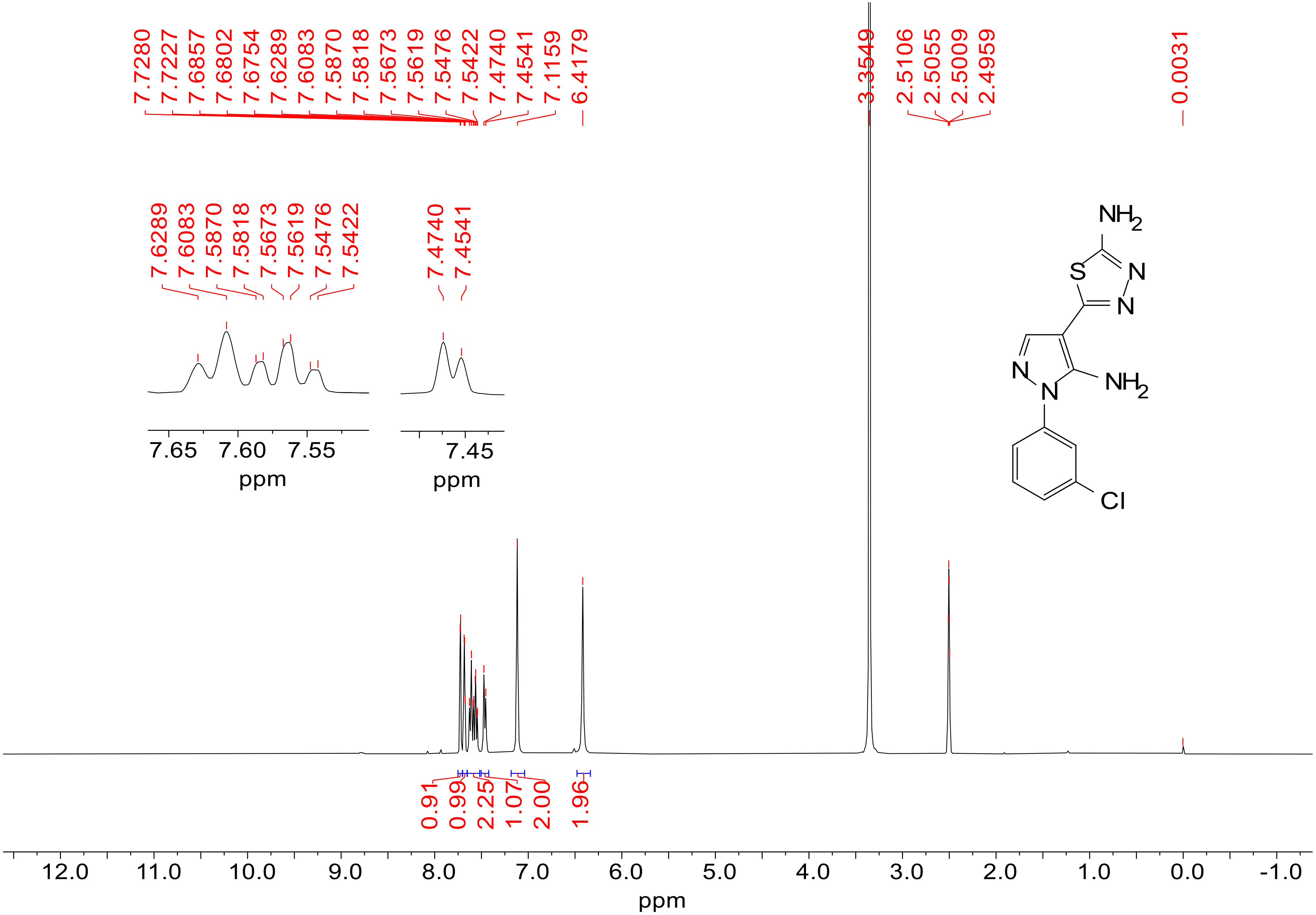

^{13}C NMR of compound **2b**

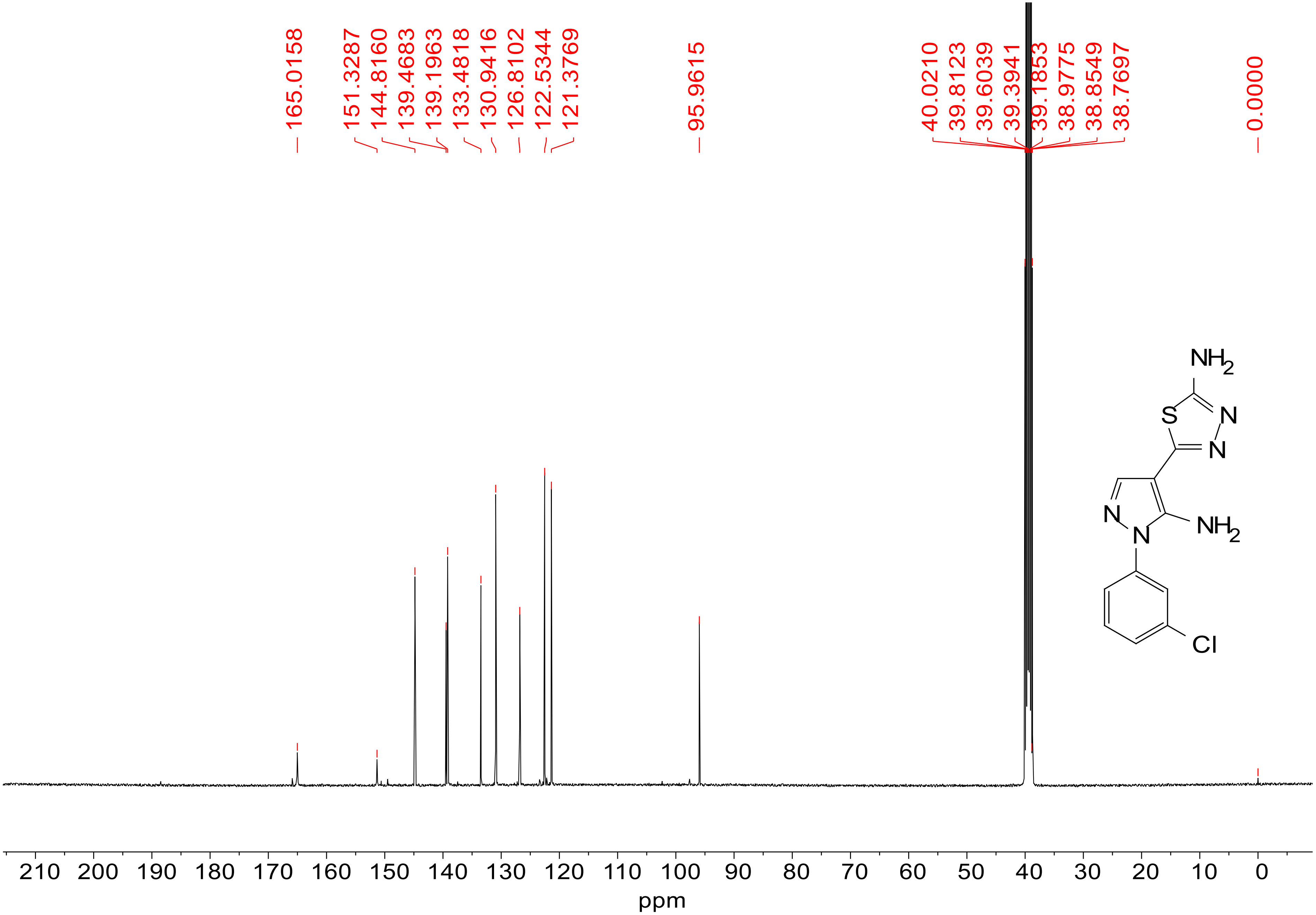

^{1}H NMR of compound **2c**

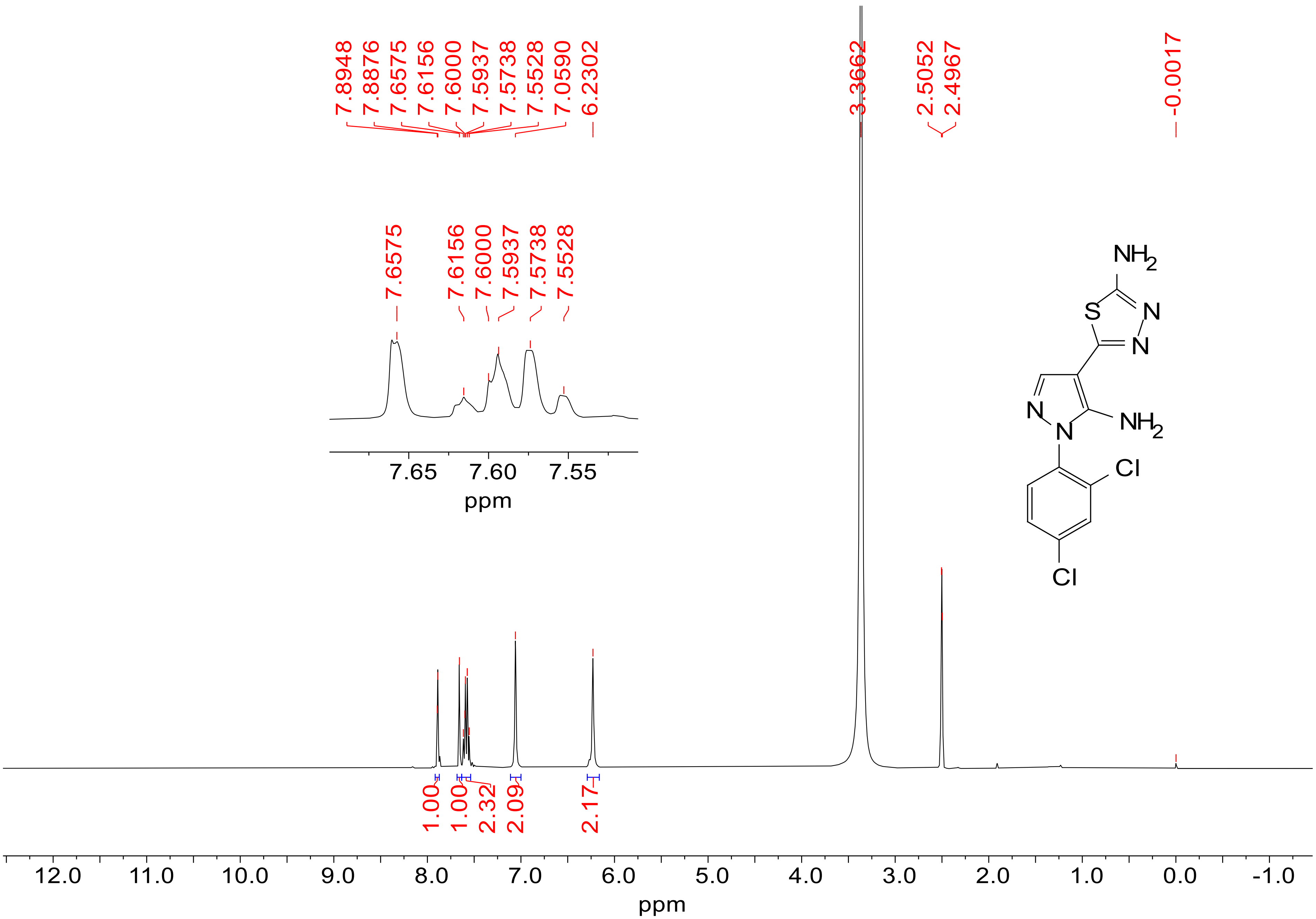

^{13}C NMR of compound **2c**

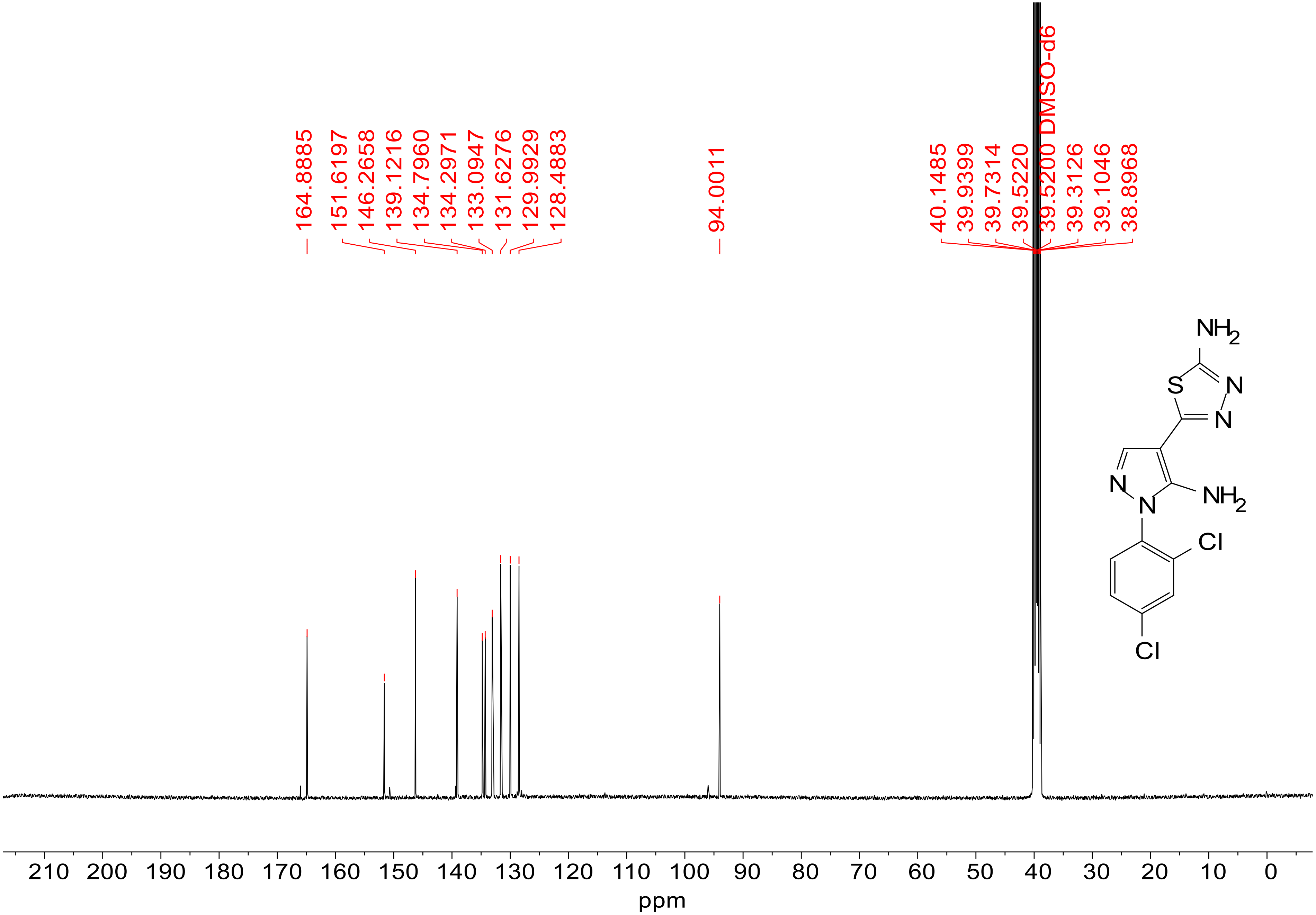

^{1}H NMR of compound **2d**

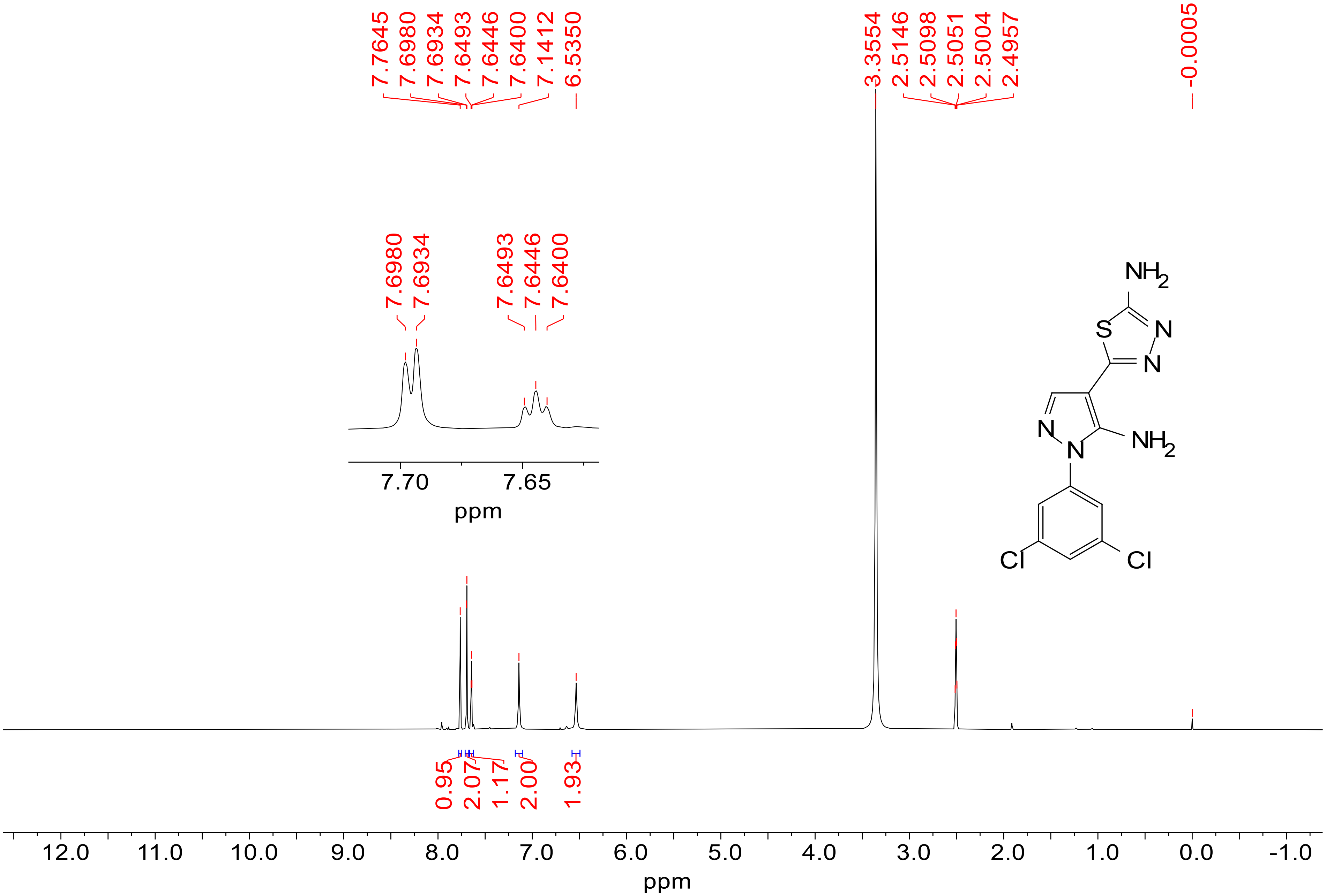

^{13}C NMR of compound **2d**

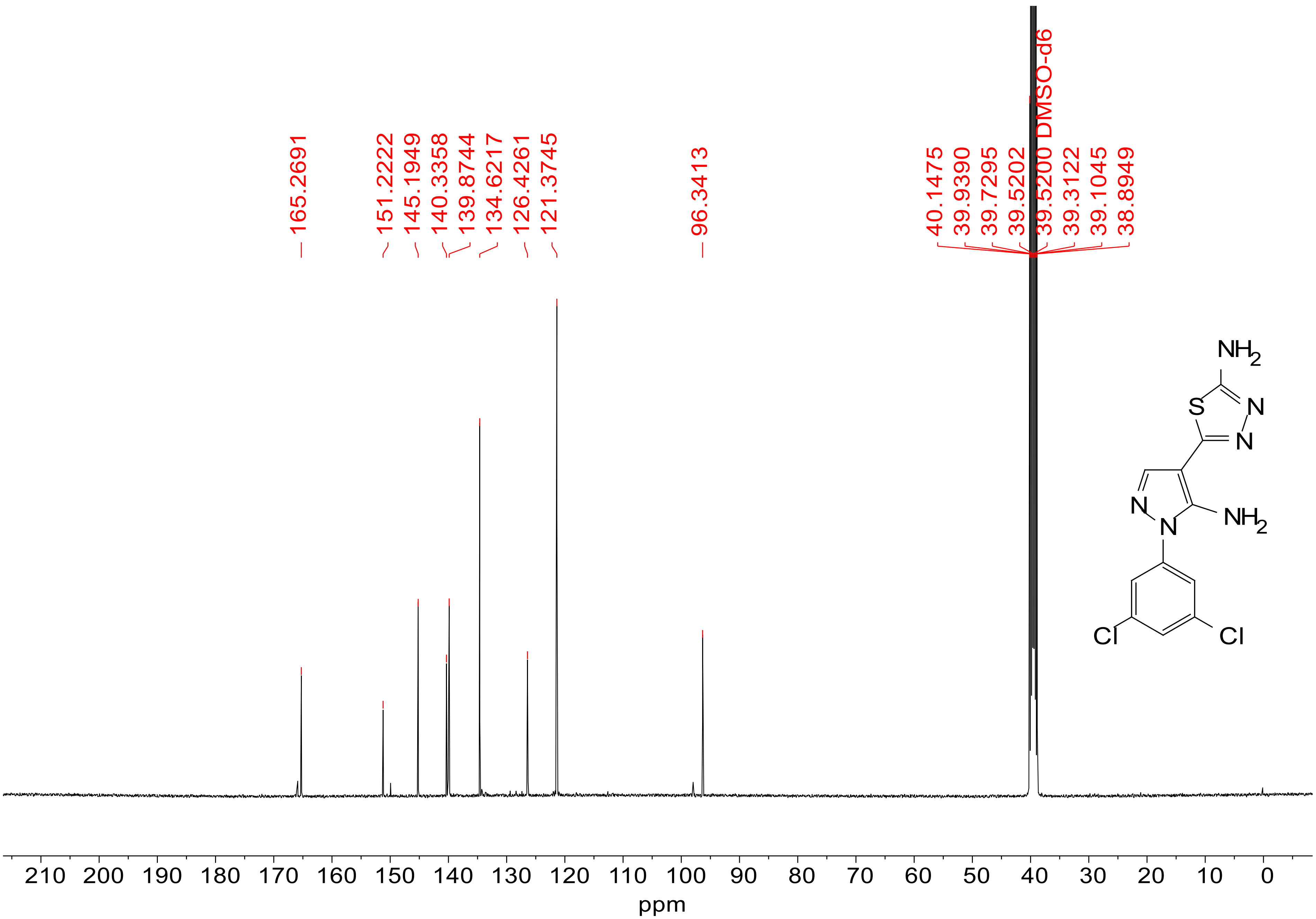

^{1}H NMR of compound **2e**

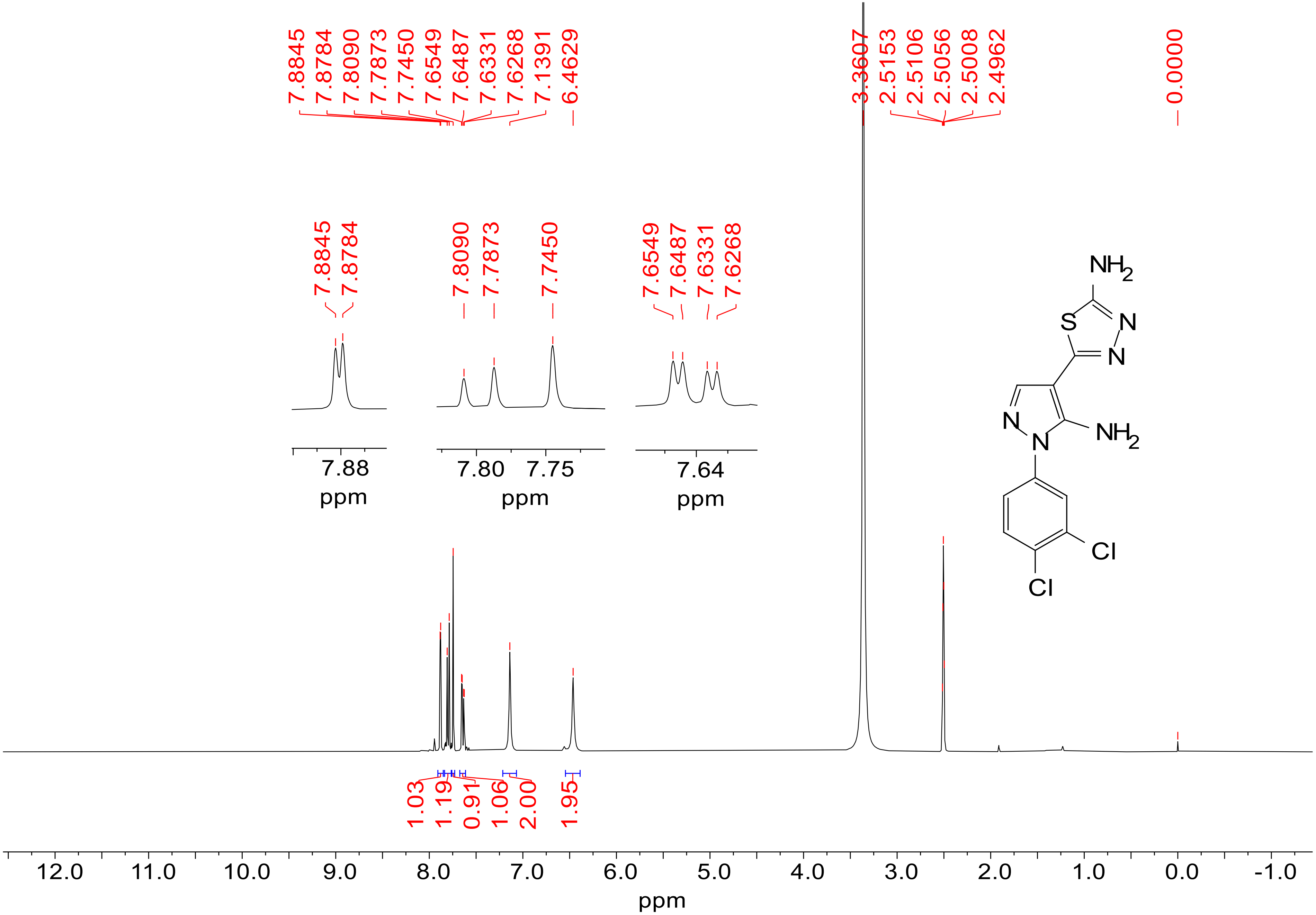

^{13}C NMR of compound **2e**

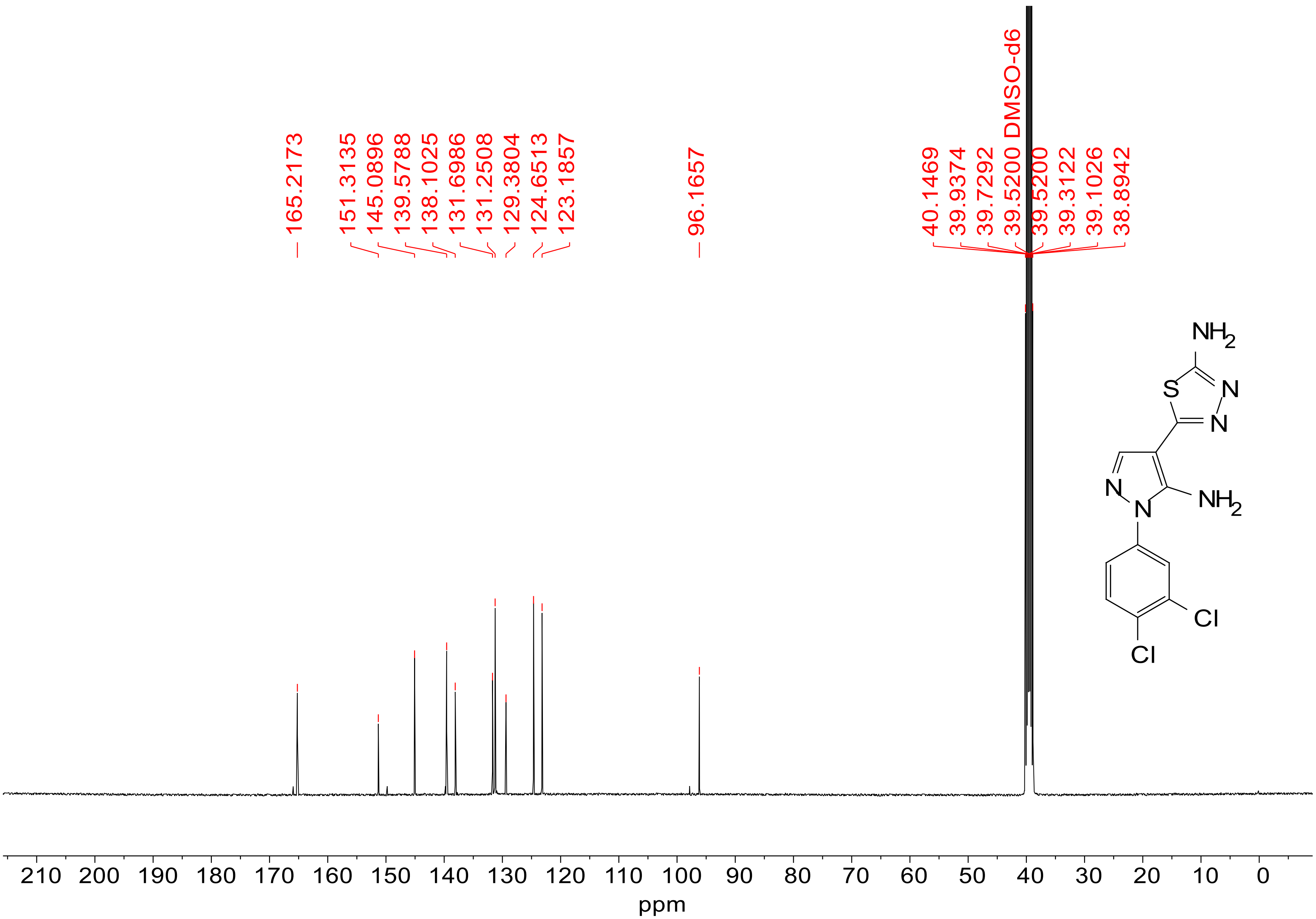

^{1}H NMR of compound **2f**

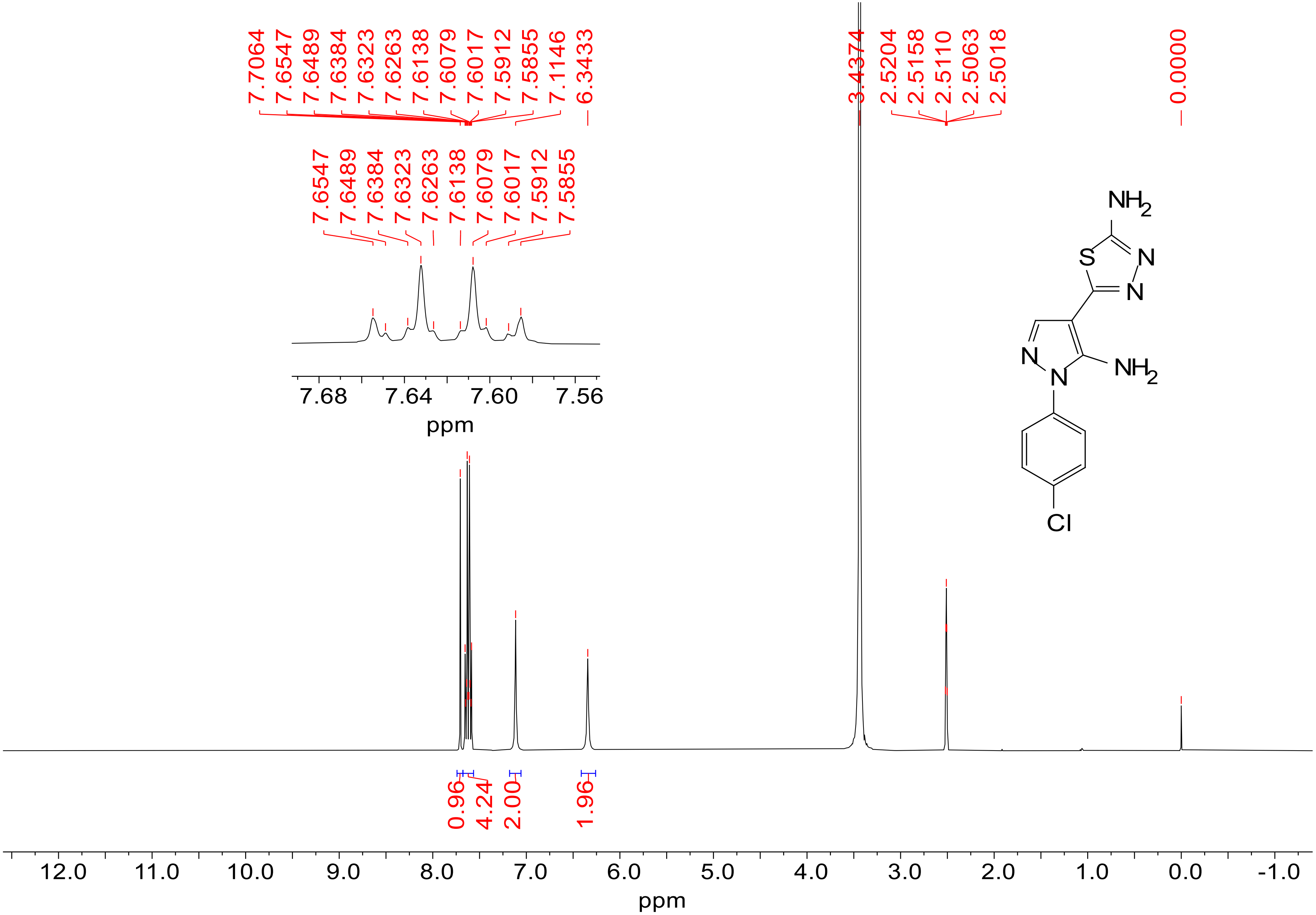

^{13}C NMR of compound **2f**

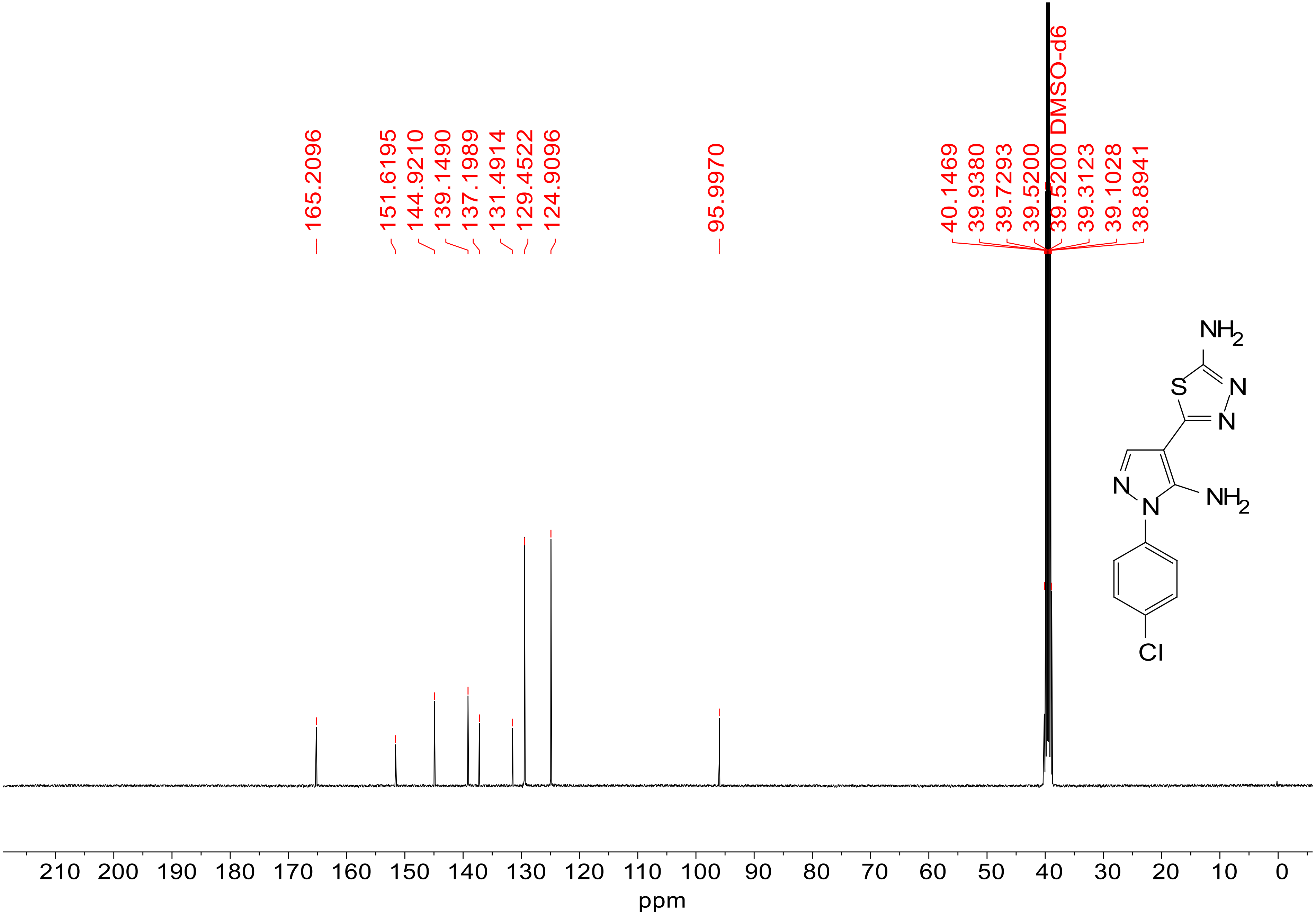

^{1}H NMR of compound **2g**

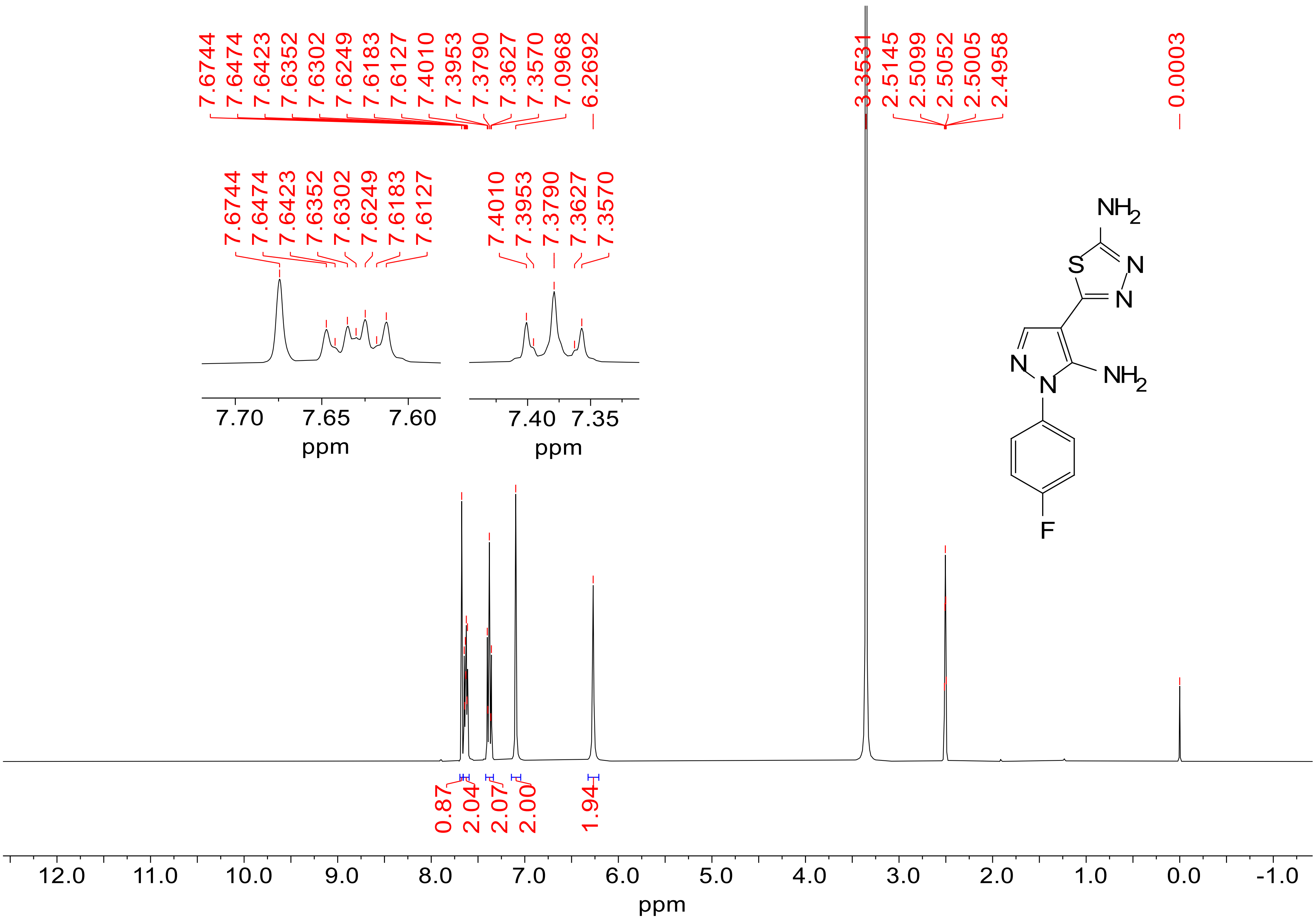

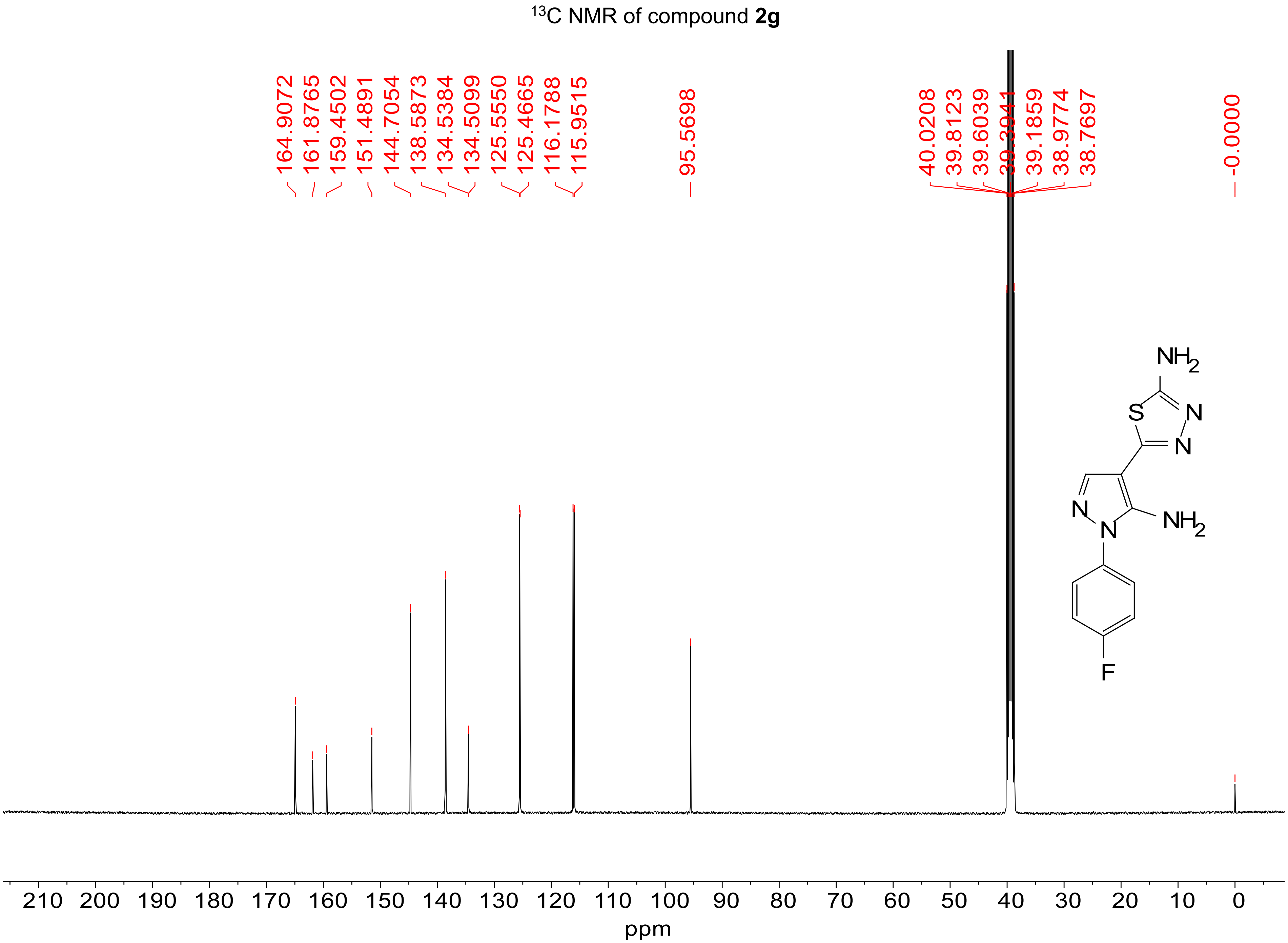
13C NMR of compound 2g
164.9072
161.8765
159.4502
151.4891
144.7054
138.5873
134.5384
134.5099
125.5550
125.4665
116.1788
115.9515
95.5698
40.0208
39.8123
39.6039
39.3941
39.1859
38.9774
38.7697
-0.0000
NH2
S
N
N
N
N
NH2
F
210 200 190 180 170 160 150 140 130 120 110 100 90 80 70 60 50 40 30 20 10 0
ppm

^{1}H NMR of compound **2h**

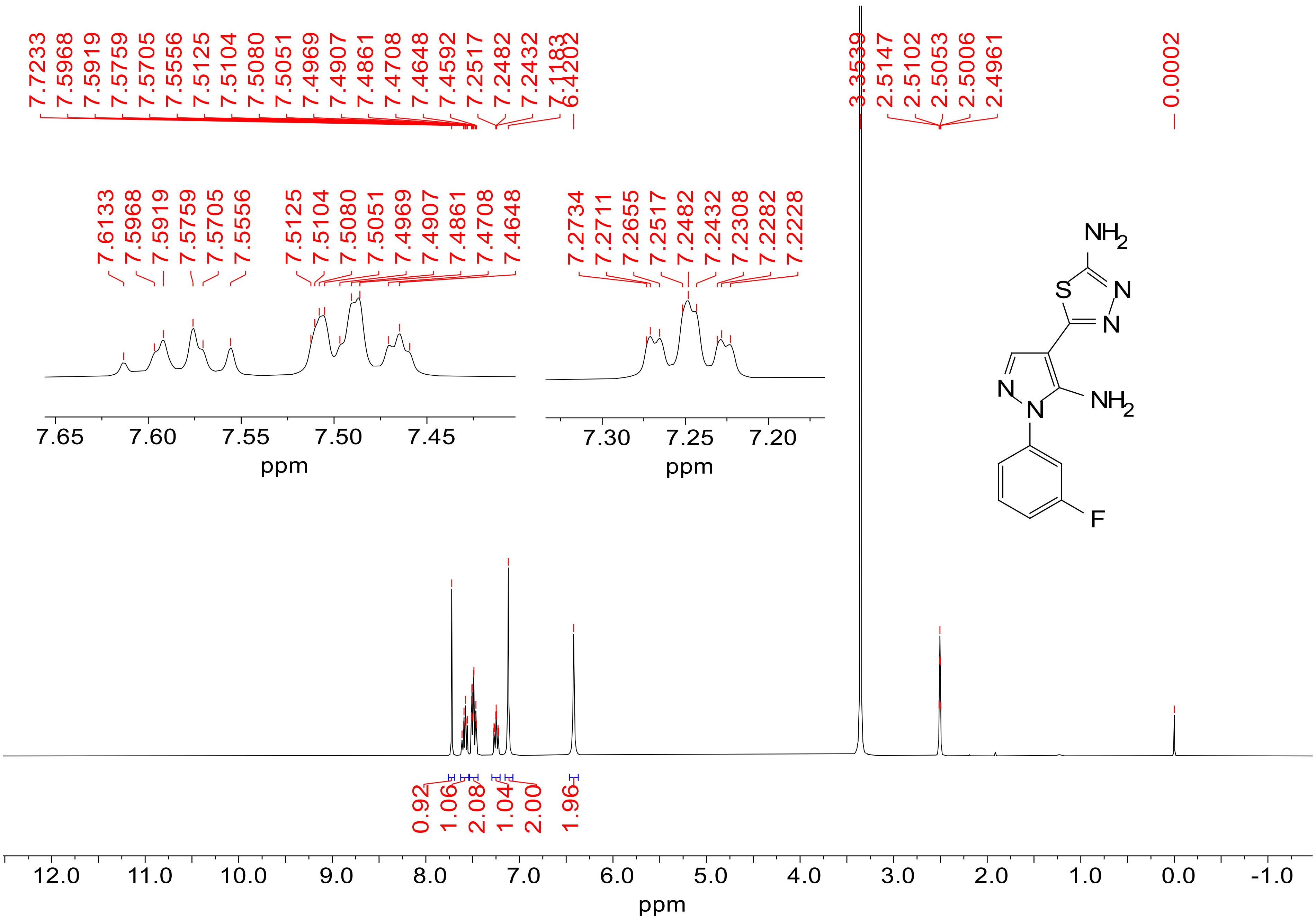

^{13}C NMR of compound **2h**

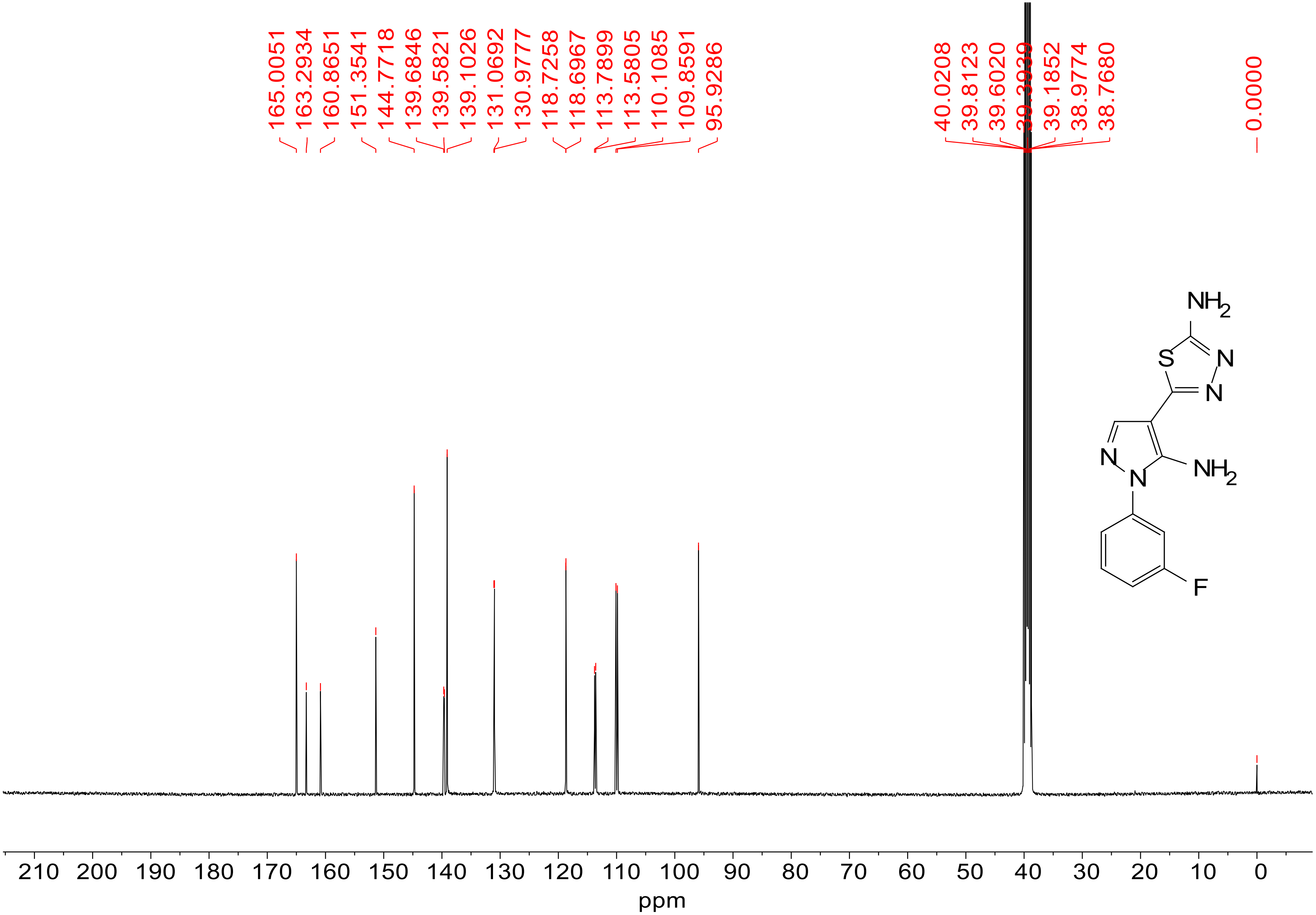

^{1}H NMR of compound **2i**

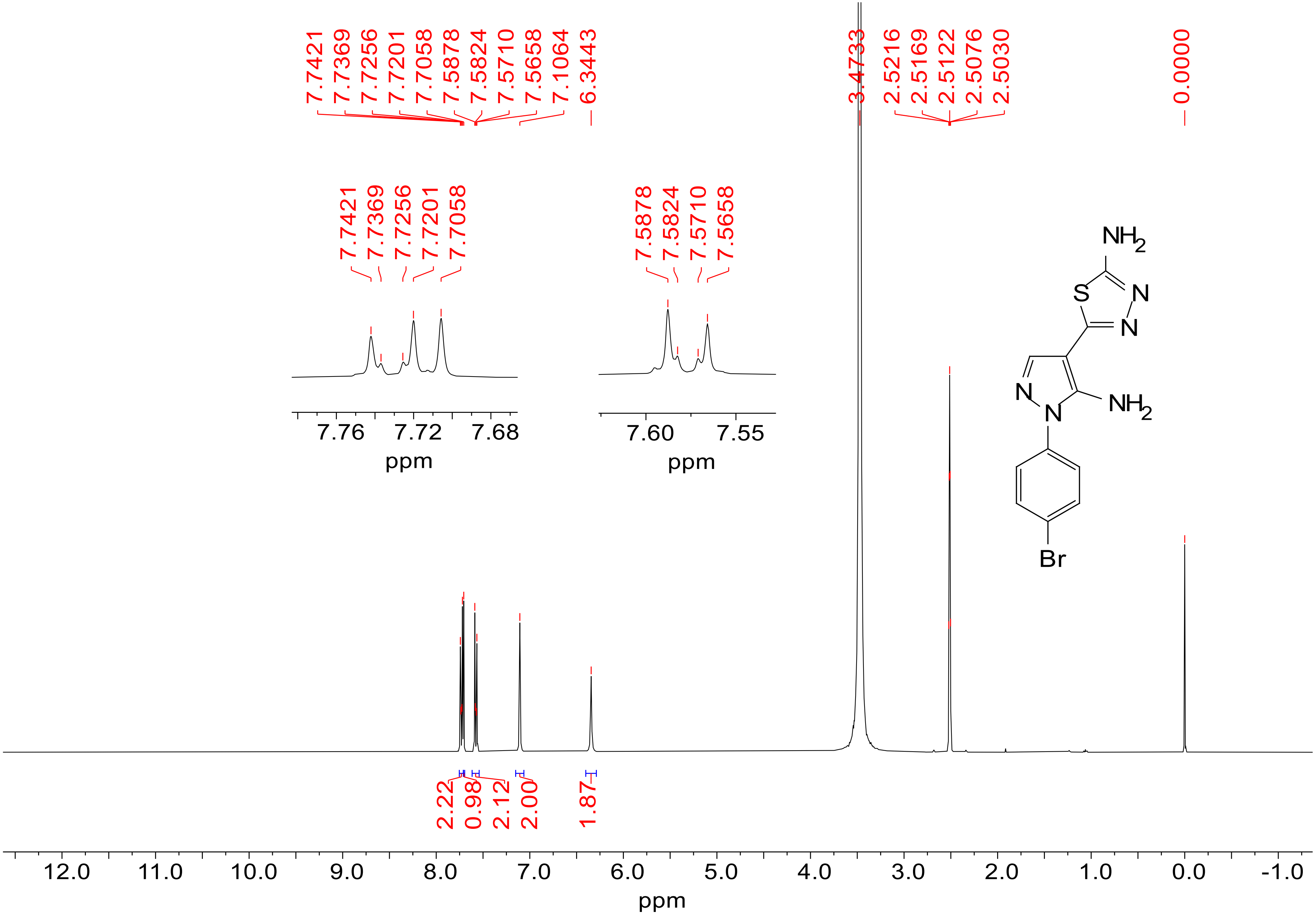

^{13}C NMR of compound **2i**

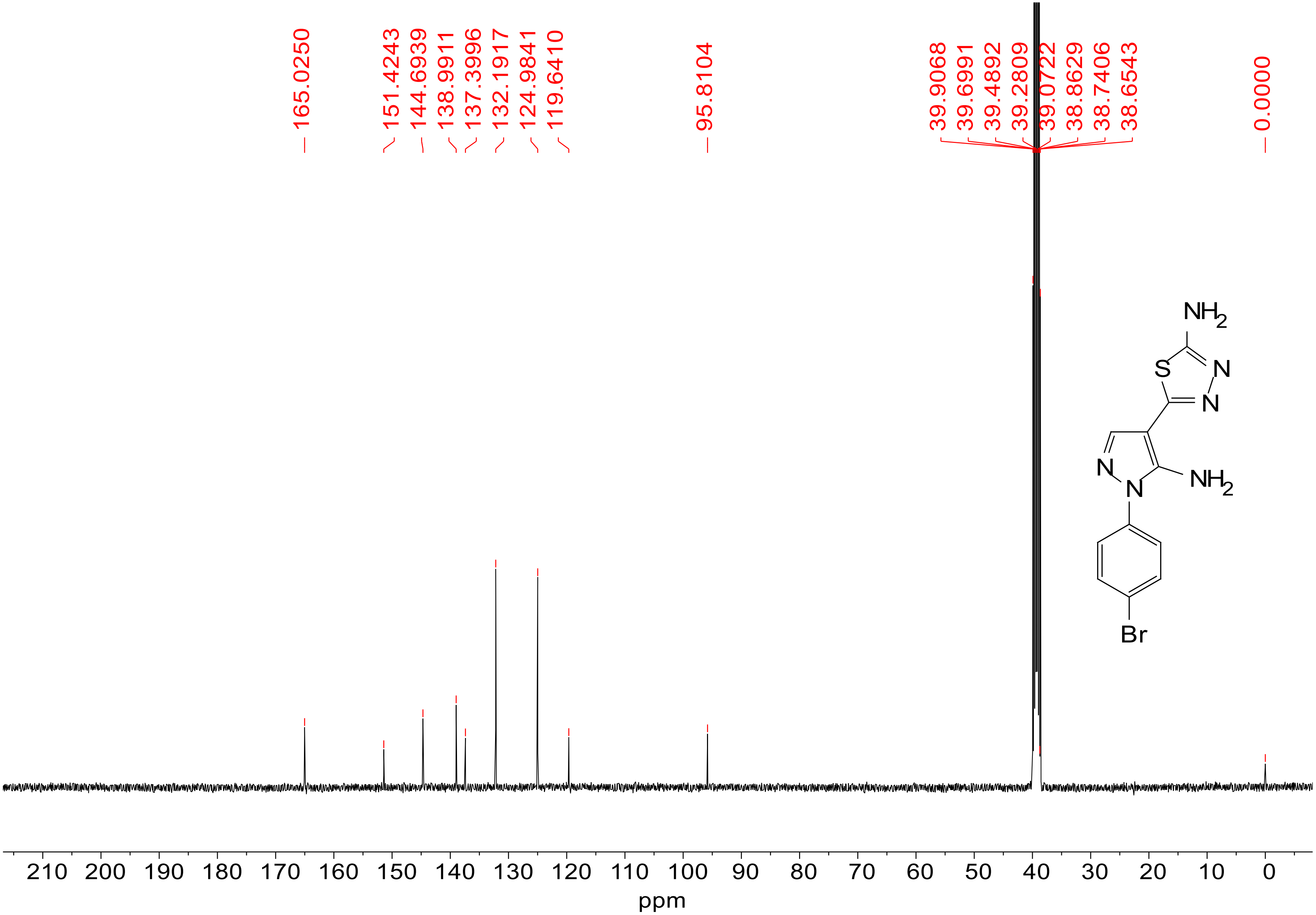

^{1}H NMR of compound **2j**

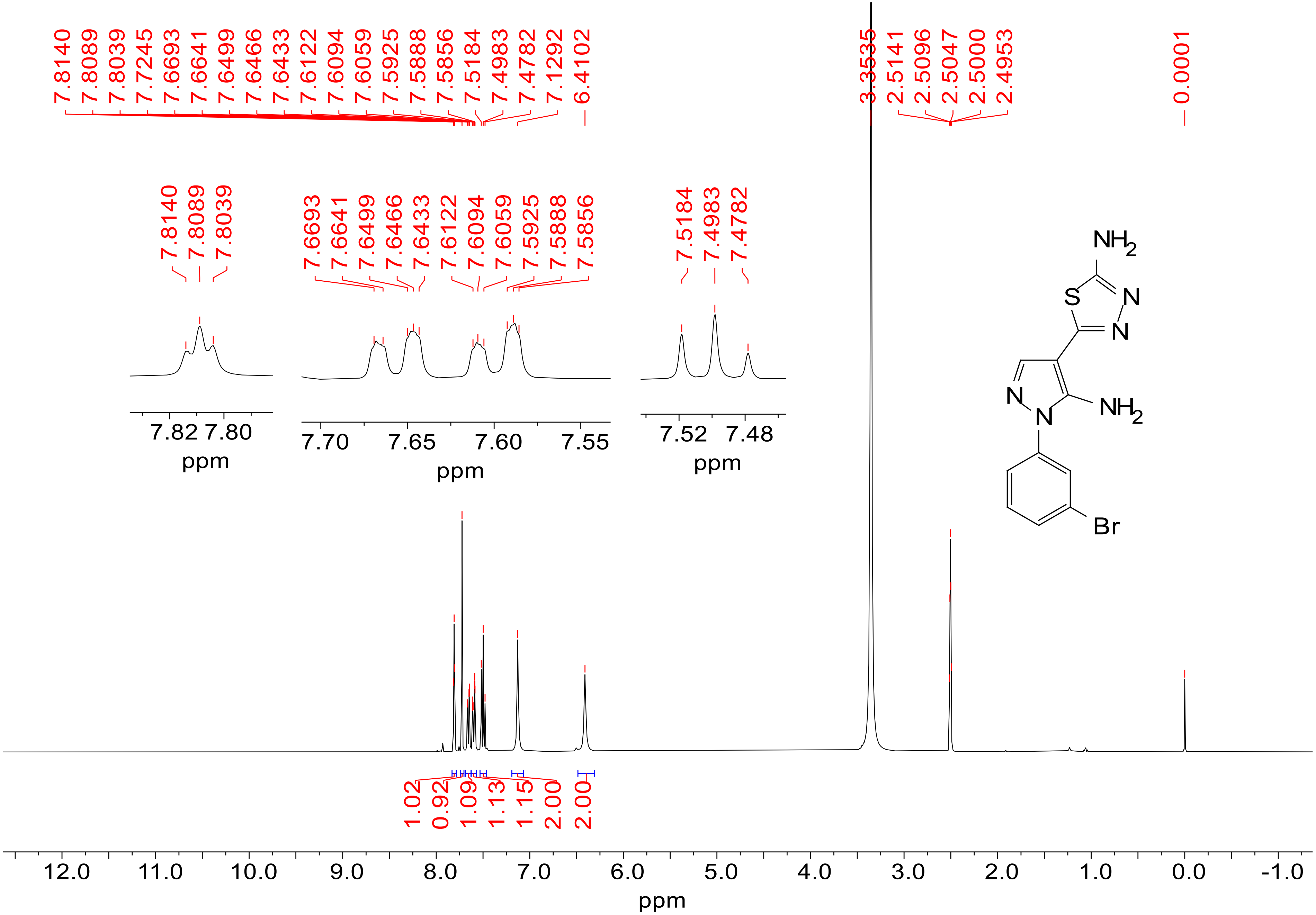

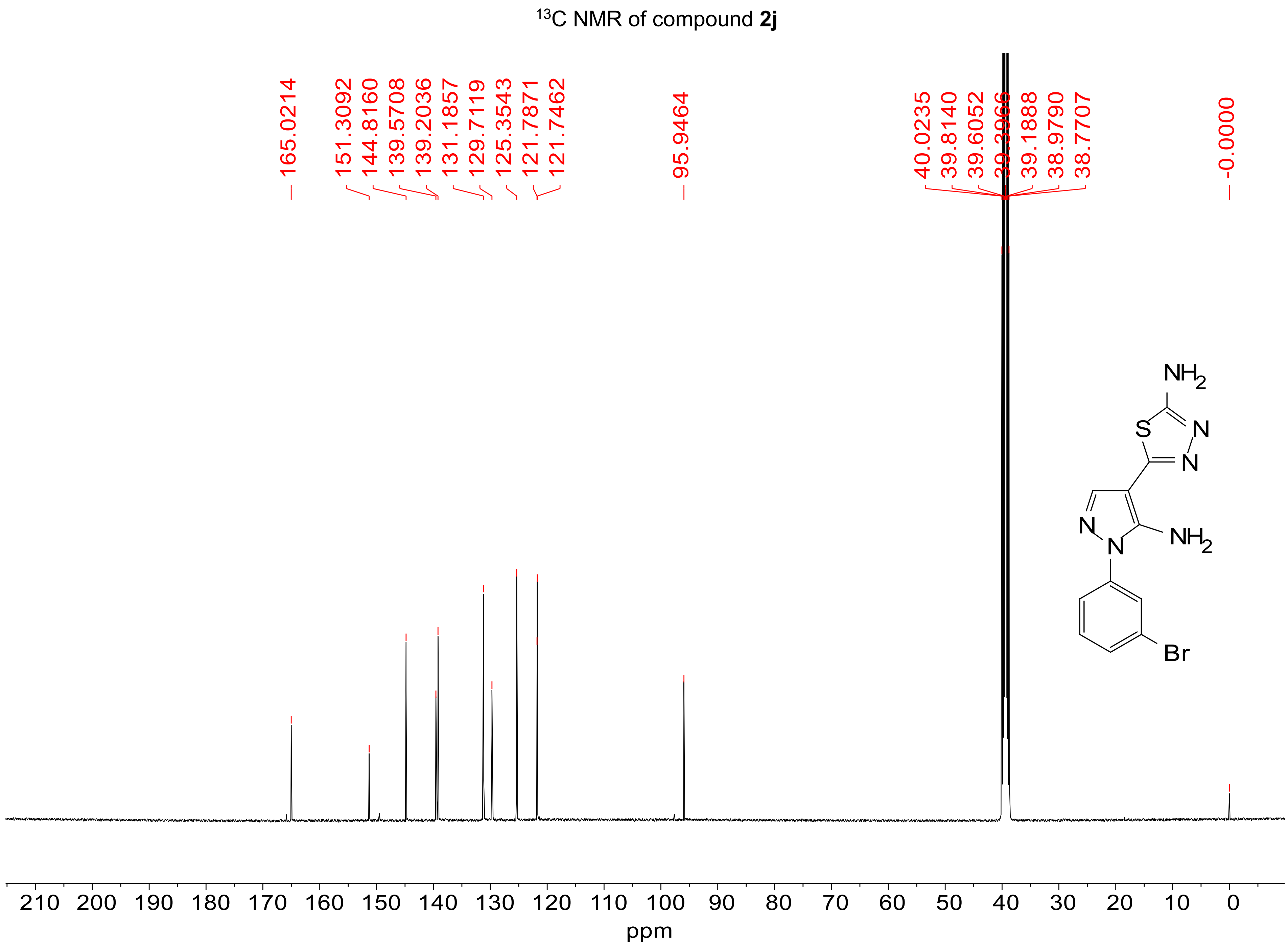

13C NMR of compound 2j
165.0214
151.3092
144.8160
139.5708
139.2036
131.1857
129.7119
125.3543
121.7871
121.7462
95.9464
40.0235
39.8140
39.6052
39.3966
39.1888
38.9790
38.7707
-0.0000
NH2
S
N
N
N
N
NH2
Br
210 200 190 180 170 160 150 140 130 120 110 100 90 80 70 60 50 40 30 20 10 0
ppm

^{1}H NMR of compound **2k**

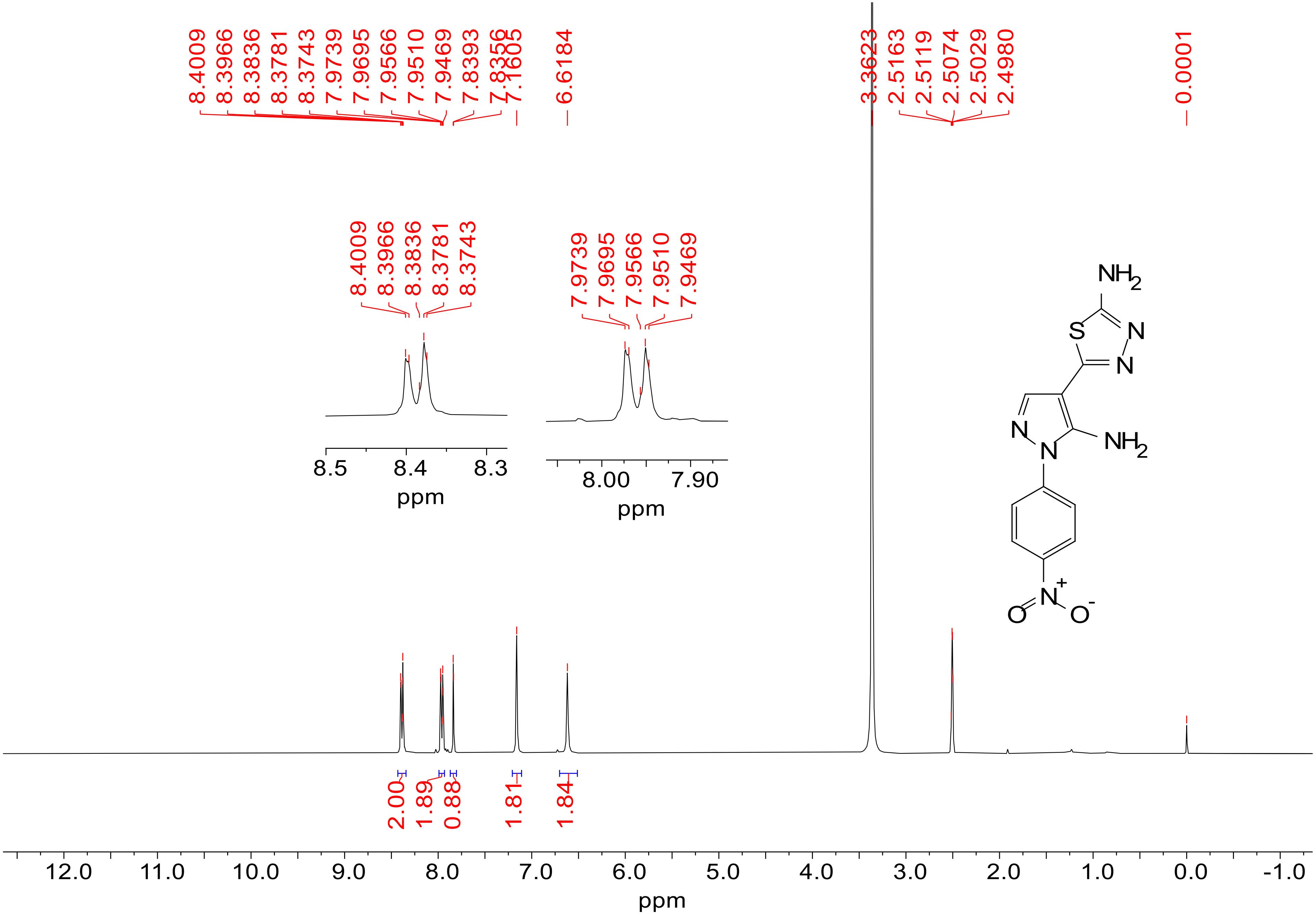

^{13}C NMR of compound **2k**

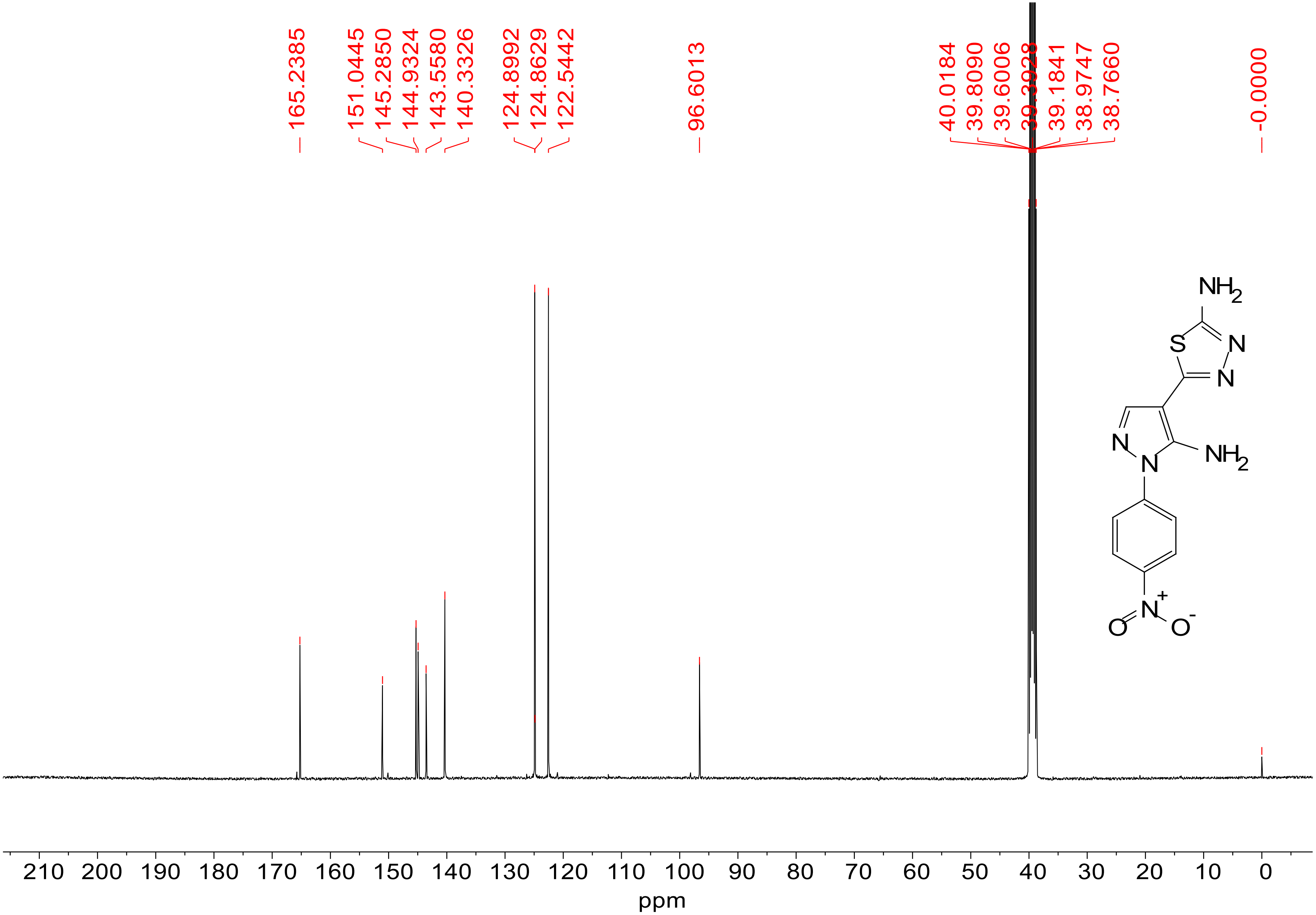

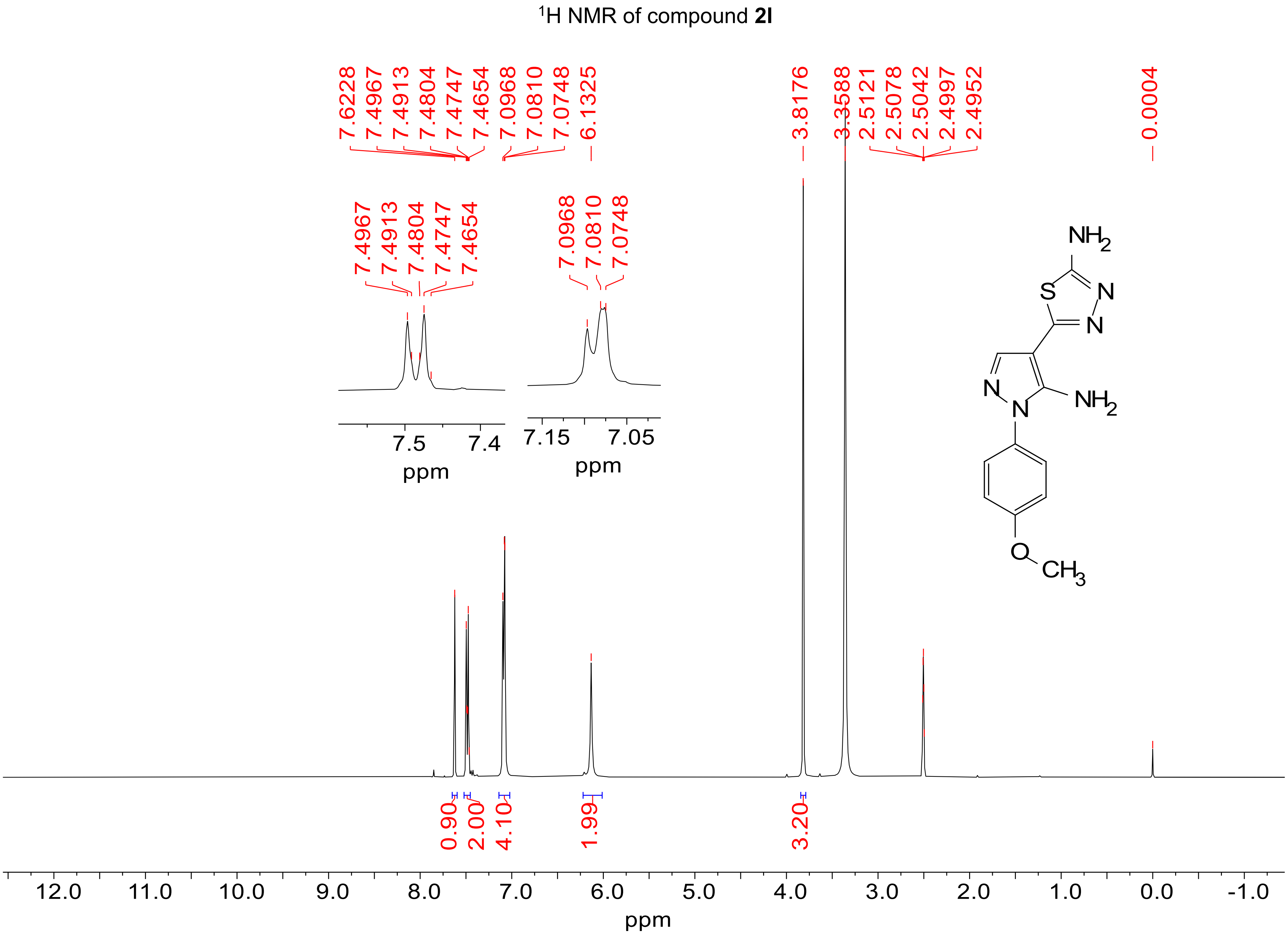

^{1}H NMR of compound **2l**
7.6228
7.4967
7.4913
7.4804
7.4747
7.4654
7.0968
7.0810
7.0748
6.1325
3.8176
3.3588
2.5121
2.5078
2.5042
2.4997
2.4952
0.0004
7.5
7.4
ppm
7.15
7.05
ppm
NH2
S
N
N
N
N
NH2
O
CH3
0.90
2.00
4.10
1.99
3.20
12.0
11.0
10.0
9.0
8.0
7.0
6.0
5.0
4.0
3.0
2.0
1.0
0.0
-1.0
ppm

^{13}C NMR of compound **2l**

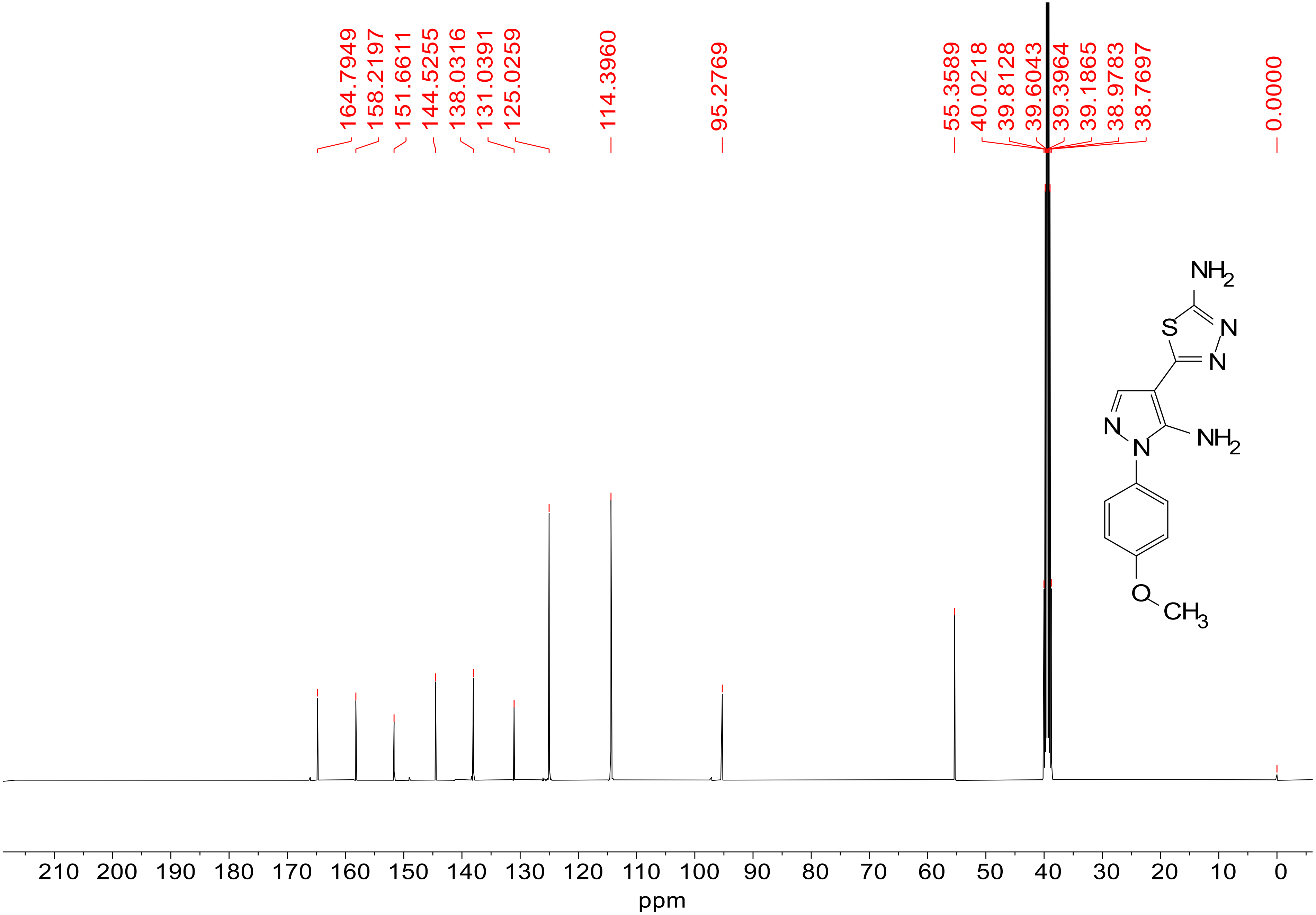

Supplement: Supplementary file 1 [file molecules-29-03544-s001.zip › supplementary material (20.06).pdf]
